# Safety of single low-dose primaquine in glucose-6-phosphate dehydrogenase deficient falciparum-infected African males: Two open-label, randomized, safety trials

**DOI:** 10.1371/journal.pone.0190272

**Published:** 2018-01-11

**Authors:** Guido J. H. Bastiaens, Alfred B. Tiono, Joseph Okebe, Helmi E. Pett, Sam A. Coulibaly, Bronner P. Gonçalves, Muna Affara, Alphonse Ouédraogo, Edith C. Bougouma, Guillaume S. Sanou, Issa Nébié, John Bradley, Kjerstin H. W. Lanke, Mikko Niemi, Sodiomon B. Sirima, Umberto d’Alessandro, Teun Bousema, Chris Drakeley

**Affiliations:** 1 Department of Medical Microbiology, Radboud University Medical Center, Nijmegen, the Netherlands; 2 Department of Biomedical Sciences, Centre National de Recherche et de Formation sur le Paludisme, Ouagadougou, Burkina Faso; 3 Disease Control & Elimination Theme, Medical Research Council Unit, Fajara, The Gambia; 4 Department of Clinical Pharmacology, University of Helsinki and Helsinki University Hospital, Helsinki, Finland; 5 Department of Immunology and Infection, London School of Hygiene and Tropical Medicine, London, United Kingdom; 6 MRC Tropical Epidemiology Group, London School of Hygiene & Tropical Medicine, London, United Kingdom; 7 Department of Disease Control, Faculty of infectious and Tropical Diseases, London School of Hygiene and Tropical Medicine, London, United Kingdom; TNO, NETHERLANDS

## Abstract

**Background:**

Primaquine (PQ) actively clears mature *Plasmodium falciparum* gametocytes but in glucose-6-phosphate dehydrogenase deficient (G6PDd) individuals can cause hemolysis. We assessed the safety of low-dose PQ in combination with artemether-lumefantrine (AL) or dihydroartemisinin-piperaquine (DP) in G6PDd African males with asymptomatic *P*. *falciparum* malaria.

**Methods and findings:**

In Burkina Faso, G6PDd adult males were randomized to treatment with AL alone (n = 10) or with PQ at 0.25 (n = 20) or 0.40 mg/kg (n = 20) dosage; G6PD-normal males received AL plus 0.25 (n = 10) or 0.40 mg/kg (n = 10) PQ. In The Gambia, G6PDd adult males and boys received DP alone (n = 10) or with 0.25 mg/kg PQ (n = 20); G6PD-normal males received DP plus 0.25 (n = 10) or 0.40 mg/kg (n = 10) PQ. The primary study endpoint was change in hemoglobin concentration during the 28-day follow-up. Cytochrome P-450 isoenzyme 2D6 (CYP2D6) metabolizer status, gametocyte carriage, haptoglobin, lactate dehydrogenase levels and reticulocyte counts were also determined.

In Burkina Faso, the mean maximum absolute change in hemoglobin was -2.13 g/dL (95% confidence interval [CI], -2.78, -1.49) in G6PDd individuals randomized to 0.25 PQ mg/kg and -2.29 g/dL (95% CI, -2.79, -1.79) in those receiving 0.40 PQ mg/kg. In The Gambia, the mean maximum absolute change in hemoglobin concentration was -1.83 g/dL (95% CI, -2.19, -1.47) in G6PDd individuals receiving 0.25 PQ mg/kg. After adjustment for baseline concentrations, hemoglobin reductions in G6PDd individuals in Burkina Faso were more pronounced compared to those in G6PD-normal individuals receiving the same PQ doses (*P* = 0.062 and *P* = 0.022, respectively). Hemoglobin levels normalized during follow-up. Abnormal haptoglobin and lactate dehydrogenase levels provided additional evidence of mild transient hemolysis post-PQ.

**Conclusions:**

Single low-dose PQ in combination with AL and DP was associated with mild and transient reductions in hemoglobin. None of the study participants developed moderate or severe anemia; there were no severe adverse events. This indicates that single low-dose PQ is safe in G6PDd African males when used with artemisinin-based combination therapy.

**Trial registration:**

Clinicaltrials.gov NCT02174900

Clinicaltrials.gov NCT02654730

## Background

Substantial reductions in malaria-related morbidity and mortality in the last decade [1] have encouraged *Plasmodium falciparum* elimination initiatives and increased interest in transmission-blocking interventions [2]. Although current first-line artemisinin-based combination therapies (ACTs) rapidly clear asexual parasites and early stage gametocytes, thereby limiting post-treatment transmission [3, 4], ACTs have incomplete activity against mature gametocytes [5, 6], the parasite stage responsible for transmission, and a proportion of patients may transmit malaria to mosquitoes after successful treatment [7, 8].

Primaquine (PQ) is the only available drug with established activity against mature *P*. *falciparum* gametocytes [9, 10] and was recommended at a single dose of 0.75 mg/kg until 2015. Recent studies in Uganda, Burkina Faso, Mali and The Gambia found that lower PQ doses of 0.20–0.40 mg/kg also clear gametocytes that persist after ACT [11–14] and prevent transmission to mosquitoes [12, 13]. These studies also demonstrated variation in transmission potential after different ACTs, with artemether-lumefantrine (AL) being associated with lower post-treatment gametocyte carriage and transmission potential compared to dihydroartemisinin-piperaquine (DP) [12, 13]. Current evidence on PQ efficacy supports the recent World Health Organization (WHO) guideline change to give a single 0.25 mg/kg PQ dose to all patients with confirmed *P*. *falciparum* (except pregnant women, infants aged < 6 months and breastfeeding women of infants aged < 6 months) on the first day of ACT in areas where there is a threat of artemisinin resistance or where *P*. *falciparum* elimination is considered [15].

However, the widespread use of PQ is limited by concerns about dose-dependent hemolysis in glucose-6-phosphate dehydrogenase deficient (G6PDd) individuals [9, 16–18]. Whilst formal safety assessments have been performed for the 14-day PQ regimen used for *Plasmodium vivax* radical cure [19–21], robust data on the safety of single low-dose PQ in G6PDd individuals is unavailable. Current evidence on the safety of a single low-dose PQ in G6PDd individuals is based on trials in which study participants were not or inefficiently screened for G6PD deficiency prior to enrolment. Three such studies in Africa reported mean absolute reductions in hemoglobin concentration 7 days post 0.75 mg/kg PQ of 1.1–2.5 g/dL, and mean relative reductions compared to baseline of 7–20% in G6PDd individuals [16, 22, 23]. Occassionally, moderate or severe transient anaemia was observed after a single PQ-dose of 0.75 mg/kg [16]. More recently, the safety of the 0.25 mg/kg PQ dose was reported as ancillary analysis after a malaria elimination campaign on the Thai-Myanmar border [24], in a gametocyte clearance efficacy study in Tanzania [25], and in a randomized controlled safety trial in Senegal [26]. The first two studies observed mean relative reductions in hemoglobin of 4–8% 5–8 days after 0.25 mg/kg PQ in a subpopulation of G6PDd participants [24, 25]. While all studies suggested the WHO-recommended PQ dose was safe, only the study from Tine *et al*. intensively monitored G6PDd individuals who have a higher risk of hemolysis [26]. Markers of hemolysis, including reticulocyte counts, lactate dehydrogenase (LDH), haptoglobin and bilirubin concentrations were not examined. Moreover, since the implementation of age-based dosing of PQ will inherently result in over- and under-dosing of individuals, it is important to assess PQ safety over a range of doses. Additional studies that intensively monitor changes in hemoglobin following different PQ doses in G6PDd individuals, alongside additional markers of hemolysis, may thus provide additional data on PQ safety that are needed to support policy recommendations [27].

Here, we report two linked randomized trials specifically designed to assess the tolerability and safety of two different doses of PQ in combination with artemether-lumefantrine (AL) or dihydroartemisinin-piperaquine (DP) in G6PDd African males with asymptomatic *P*. *falciparum* malaria.

## Methods

### Study design

Two open-label, randomized, dose-escalation trials were conducted from August 2014 to November 2015 in Banfora, in southwest Burkina Faso and from December 2015 to April 2016 in Basse Santa Su, Upper River Region, The Gambia. The studies received approval of the Interventions Research Ethics Committee of the London School of Hygiene and Tropical Medicine (#6523 for Burkina Faso; #8487 for The Gambia), Comité d’Ethique pour la Recherche en Santé, Ministère de la Santé du Burkina Faso (#2014-02-09) and Comité Technique d’Examen des Demandes d’Autorisation d’Essais Cliniques, Ministère de la Santé du Burkina Faso (#50002520146EC00000) and The Gambia Government/MRC Joint Ethics Committee (SCC 1391, 17 August 2015). Both studies were registered at ClinicalTrials.gov (NCT02174900 and NCT02654730). Written informed consent was obtained from all participants or from their parents or guardians prior to screening and enrolment.

We included G6PDd males to reduce the possibility of incorrect classification of G6PDd in heterozygous females. In Burkina Faso, a first phase of screening in the target population was performed in the community using the CareStart™ G6PD Rapid Diagnostic Test (G6PD RDT; Access Bio, Inc. Somerset, USA). To minimize the risk for trial participants in this first formal safety study on single low-dose PQ in G6PDd individuals, we excluded symptomatic patients and anemic individuals. Potentially eligible individuals were invited at the clinic to enroll males (age 18–45 years) with asymptomatic *P*. *falciparum* malaria, hemoglobin concentration ≥11 g/dL, and confirmed G6PD status by the “Beutler’s” fluorescent spot test [28]. Additional exclusion criteria were: fever (tympanic temperature >37.5°C) or history of fever in the last 24 hours; enrolled in another clinical trial; severe illness or danger signs; active infection other than malaria; history of severe chronic illness; known allergy to study medications; use of antimalarials in the previous 2 weeks; use of PQ in the previous 4 weeks; blood transfusion in the previous 90 days; non-falciparum malaria co-infection; current tuberculosis or anti-retroviral treatment; or current use of sulphonamides, dapsone, nitrofurantoin, nalidixic acid, ciprofloxacin, methylene blue, toluidine blue, phenazopyridine or co-trimoxazole. Enrolled participants were sequentially assigned to two cohorts. The first cohort consisted of one intervention group (G6PDd participants treated with AL and a single dose of 0.25 mg/kg PQ [n = 20]) and three control groups (G6PDd participants treated with AL alone [n = 10], and G6PD-normal participants treated with AL and a single dose of 0.25 mg/kg PQ [n = 10] or 0.40 mg/kg PQ [n = 10]). After the Data Safety Monitoring Board reviewed safety data, enrolment of the second cohort was initiated, consisting of one intervention group (G6PDd participants treated with AL and a single dose of 0.40 mg/kg PQ [n = 20]).

In The Gambia, males (age ≥10 years) with asymptomatic *P*. *falciparum* malaria were screened using identical procedures to Burkina Faso and randomized to one intervention group (G6PDd participants treated with DP and a single dose of 0.25 mg/kg PQ [n = 20]) or one of three control groups (G6PDd participants treated with DP alone [n = 10], or G6PD-normal participants treated with DP and a single dose of 0.25 mg/kg PQ [n = 10] or 0.40 mg/kg PQ [n = 10]). Whilst the Data Safety Monitoring Board review was favorable to escalating the treatment to a single dose of 0.40 mg/kg PQ, this could not be completed for logistical and financial reasons.

### Procedures

All participants in Burkina Faso received 6 doses of 4 tablets of AL twice daily over three days (Coartem [20 mg artemether and 120 mg lumefantrine], Novartis Pharma AG, Basel, Switzerland). In The Gambia, participants received a daily dose of DP (Eurartesim [40mg piperaquine and 320mg dihydroartemisinin], Sigma-Tau IFR S.p.A, Italy) over three days; the dose was calculated based on body weight, on an empty stomach. For groups receiving PQ, doses were prepared as previously described [11]: PQ tablets containing 26.3 mg PQ phosphate USP, equivalent to 15 mg of PQ base (Sanofi, New York, USA) were crushed and dissolved in 15 mL of drinking water to produce a stable 1 mg/mL PQ base solution. We drew up the assigned dose to the nearest 0.5 mL. In Burkina Faso, PQ was administered under supervision together with the first AL dose with biscuits and juice. In The Gambia, PQ was administered under supervision with a glass of orange juice 30 minutes after the first dose of DP.

Participants returned to the study clinic for follow-up on the evening of day 0, twice daily on days 1, 2, and 3 (morning and evening), and once daily on days 4, 5, 7, 10, 14 and 28, and on additional days if they developed symptoms. All adverse events (AEs) were scored based on the Division of Microbiology and Infectious Diseases (DMID) toxicity table and graded as mild/grade 1 (awareness of symptoms that were easily tolerated and did not interfere with usual daily activity), moderate/grade 2 (discomfort that interfered with or limited usual daily activity), or severe/grade 3 (disabling, with inability to perform usual daily activity). Tympanic temperature was measured and recorded as fever grade 1 (37.6–38.0°C), grade 2 (> 38.0–39.0°C), or grade 3 (> 39.0°C). Causality of AEs was classified as unrelated, unlikely, possibly, probably or definitely related to the trial drugs.

Hemoglobin concentration was assessed at each visit by self-calibrating HemoCue 201+ photometers (Hemocue; Ängelholm, Sweden). Blood samples were taken for microscopy on days 0, 1, 2, 3, 7, 10, 14 and 28, and stained with 10% Giemsa for 10 minutes before screening for asexual parasites and gametocytes in 100 microscopic fields and quantifying against 200 and 500 leukocytes, respectively, and translating to parasite counts/μl assuming 8000 leukocytes per μl. Standard hematological and biochemical tests were done twice daily on days 0 and 1, and once daily on days 3, 7, 14 and 28 using for hematology the Cell-Dyn Ruby [Abbott Diagnostics, Wiesbaden, Germany] in Burkina Faso and the Medonic M-Series analyzer [Boule Medical AB, Spånga, Sweden] in The Gambia and for biochemistry the BS-200 analyzer [Shenzhen Mindray Bio-Medical Electronics Co., Ltd, Guangdong Sheng, China] in Burkina Faso and the Vitros 350 analyzer [Ortho Clinical Diagnostics, Raritan, NJ, US] in The Gambia. Assessments include total bilirubin and reticulocyte counts. Gametocyte detection by *Pfs25* mRNA quantitative nucleic acid sequence-based amplification (QT-NASBA, Burkina Faso) or *Pfs25* mRNA quantitative reverse-transcriptase PCR (qRT-PCR, The Gambia) [29] was done on days 0, 3 and 7. Urine dipstick to detect blood/hemoglobin and urobilinogen was done twice daily on days 0, 1, 2 and 3 and once daily on days 4, 5, 7 and 28. Remaining plasma samples from biochemical tests from the study in Burkina Faso were used for haptoglobin and LDH measurements.

Whole blood samples collected on day 0 were used for genotyping the gene for human cytochrome P-450 isoenzyme 2D6 (CYP2D6) to investigate any influence on PQ pharmacokinetics [30–32], and *G6PD* sequence variants 202A and 376G, which define the major African G6PD deficient variant A-. *CYP2D6* and *G6PD* genotyping was performed as described previously [13]. For *G6PD* the two sequence variants (202A and 376G) were genotyped in OpenArray or single assay format (Thermo Fisher). For *CYP2D6* 21 sequence variants were genotyped for detection of major alleles *2, *3, *4, *6, *7, *8, *9, *10, *11, *15, *17, *18, *19, *20, *29, *40, and *41 with Quantstudio 12K Flex OpenArray with TaqMan assays (Thermo Fisher). If no *CYP2D6* mutations were detected, participants were genotyped as *1. Also three copy number variation (CNV) assays targeting introns 2 and 6 as well as exon 9 of the gene were used for copy number analysis, allowing for partial detection of potential *CYP2D6* hybrid alleles with the pseudogene *CYP2D7* [33]. From genotype, Activity Score (AS) for each participant was calculated and metabolizer phenotype was inferred accordingly: an AS of 0.0 = poor metabolizer (PM) phenotype, AS of 0.5 or 1.0 = intermediate metabolizer (IM) phenotype, AS of 1.5 or 2.0 = extensive metabolizer phenotype, and an AS of 2.5 or more = ultra-rapid metabolizer (UM) phenotype [34–36].

### Primary and secondary endpoints

The study objective was to evaluate the tolerability and safety of single doses of PQ at two different dosages when administered with AL or DP to G6PDd males with asymptomatic *P*. *falciparum* infections. The primary study outcome was the hemoglobin concentration during the 28-day follow-up relative to baseline value (safety). Secondary outcomes included frequency and severity of AEs, and changes in hemoglobin concentration > -2.5 g/dL (safety outcomes), and post-treatment gametocyte prevalence (efficacy outcome). Secondary outcomes that were only assessed for Burkina Faso included other hematological (e.g. haptoglobin) and biochemical (e.g. LDH) parameters during follow-up.

### Statistical analysis

Statistical analysis was performed using Stata (v13, StataCorp). Student *t*-test was used to compare hemoglobin concentration (g/dL) at baseline between study groups; other continuous variables were compared by non-parametric Wilcoxon-rank sum. Proportions were compared by chi-square test. Repeated measures mixed models (xtmixed with independent covariance structure) were used for pairwise comparisons of hemoglobin concentrations. Hemoglobin concentration during follow-up relative to baseline value was expressed as absolute and relative changes. To limit multiple comparisons, we restricted comparisons of changes in hemoglobin concentration to days 3, 7, 14 and 28 [16, 22, 23]. None of the *P*-values were adjusted for multiple comparisons. We adjusted all comparisons for baseline hemoglobin concentration using multivariate linear regression; models only contained treatment group and baseline hemoglobin. A drop of >2.5 g/dL at any time-point during follow-up was analyzed as dichotomous variable. Moderate anemia was defined as a hemoglobin concentration <8 g/dL; severe anemia as a hemoglobin concentration <5 g/dL [37]. The difference in AEs among groups was calculated as i) the proportion of individuals developing AEs in each group and ii) the frequency of AEs in each group (absolute number of AEs per participant). This study was not powered to assess PQ gametocytocidal efficacy [11, 12] and analyses of gametocyte prevalence by molecular methods at baseline and during follow-up are descriptive.

We explored whether genetically inferred CYP2D6 metabolizer status was a relevant factor in determining hemolysis after PQ by adding inferred CYP2D6 metabolizer status (poor/intermediate versus extensive/ultra rapid) to multivariate linear regression models with absolute or relative reductions in hemoglobin concentration on day 7 compared to baseline as dependent variable and baseline hemoglobin and phenotypically determined G6PD status as independent variables. This analysis was restricted to PQ-treated study participants.

### Sample size

We did not perform a formal sample size calculation for this safety study. We assumed the probability of the occurrence of at least one serious adverse event (SAE) was 10% for G6PDd individuals. Twenty individuals in each of the 2 intervention groups (G6PDd participants receiving PQ) provide an 88% probability of detecting at least one individual with an SAE and 61% probability of detecting 2 or more individuals with SAEs. The control groups were included to support the interpretation of hemoglobin concentrations following treatment; the size of these control groups was based on expert opinion (Walter Reed Army Institute of Research, Tafenoquine group).

## Results

### Overview of trials

**Fig 1:**
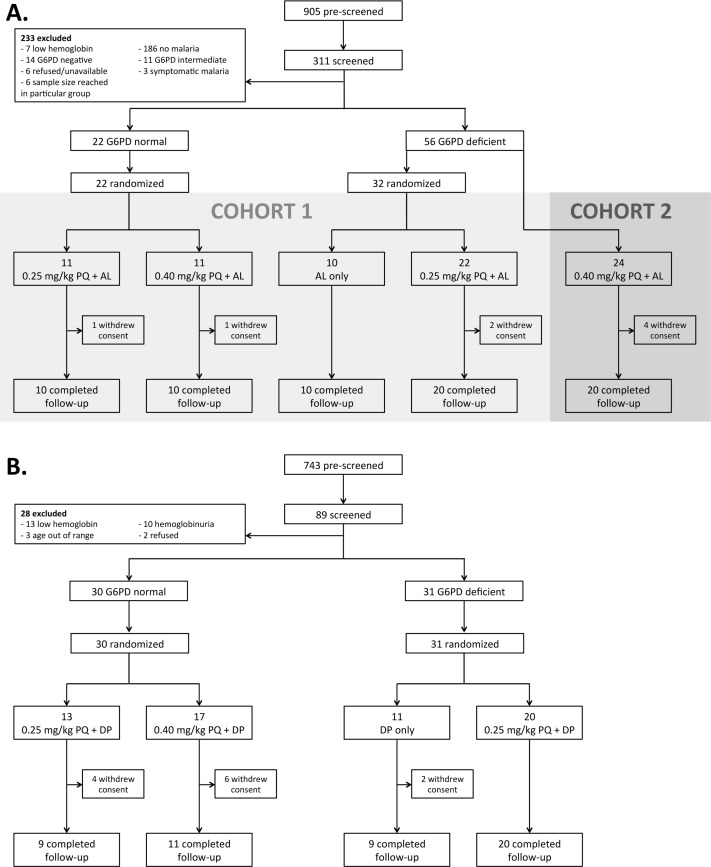
Clinical trial profile for Burkina Faso (A) and The Gambia (B). Abbreviations: AL, artemether-lumefantrine; G6PD, glucose-6-phosphate dehydrogenase; PQ, primaquine; DP, dihydroartemisinin-piperaquine.

Initial G6PD-status screening by G6PD RDT in Burkina Faso involved 905 males; 311 were further screened in the clinic and 78 were enrolled (Fig 1A). Seventy participants completed follow-up, 8 were lost to follow-up prior to day 3 (Fig 1A). In The Gambia 743 males were pre-screened and 61 were enrolled of whom 49 completed follow-up and 12 withdrew within the first week (Fig 1B). Mean hemoglobin concentration at baseline was higher in G6PD-normal compared to G6PDd individuals in Burkina Faso (mean difference 0.84 g/dL; 95% CI, 0.16–1.53; *P* = 0.016) but not in The Gambia (mean difference -0.33 g/dL; 95% CI, -1.09–0.43; *P* = 0.38). Other baseline characteristics were not significantly different between G6PDd and G6PD-normal individuals or between study groups (Table 1). Median asexual parasite density at enrolment was 85.5 (interquartile range [IQR], 43.0–269.0 parasites/μL) in Burkina Faso; gametocyte prevalence was 3.8% (3/78) by microscopy and 89.9% (62/69) by QT-NASBA (Table 1). In The Gambia, only 9.8% (6/61) of study participants had asexual parasites detected by microscopy at enrolment with a median parasite density of 768.0 (IQR, 320.0–1520.0 parasites/μL) in parasite positive individuals. Gametocyte prevalence was 1.6% (1/61) by microscopy and 18.0% (11/61) by qRT-PCR (Table 1).

**Table 1:** Baseline characteristics of enrolled subjects.

	Burkina Faso					
	G6PD Normal		G6PD Deficient			
Characteristic	0.25 mg/kg PQ + AL (n = 11)	0.40 mg/kg PQ + AL (n = 11)	AL only (n = 10)	0.25 mg/kg PQ + AL (n = 22)		0.40 mg/kg PQ + AL (n = 24)
Age, y, median (IQR)	26.0 (22.0–27.0)	31.0 (21.0–40.0)	33.0 (23.0–38.0)	24.5 (21.0–32.0)		30.0 (25.5–36.0)
Hemoglobin, g/dL, mean (SD)	14.5 (1.4)	15.0 (1.6)	13.6 (0.9)	14.0 (1.6)		14.0 (1.2)
Asexual parasite density by microscopy, parasites/μL, median (IQR)	117 (44.0–537.0)	43.0 (32.0–107.0)	140.5 (28.0–276.0)	67.0 (40.0–175.0)		100.5 (54.5–821.5)
Gametocyte prevalence by microscopy, % (n/N)	0.0 (0/11)	0.0 (0/11)	10.0 (1/10)	0.0 (0/22)		8.3 (2/24)
Gametocyte prevalence by QT-NASBA, % (n/N)	100.0 (10/10)	100.0 (10/10)	80.0 (8/10)	85.0 (17/20)		89.5 (17/19)
CYP2D6 inferred poor metabolizer phenotype, % (n/N)	0.0 (0/11)	0.0 (0/11)	0.0 (0/10)	0.0 (0/20)		4.2 (1/24)
CYP2D6 inferred intermediate metabolizer phenotype, % (n/N)	27.3 (3/11)	36.4 (4/11)	30.0 (3/10)	55.0 (11/20)^a^		62.5 (15/24)
	The Gambia					
	G6PD Normal		G6PD Deficient			
Characteristic	0.25 mg/kg PQ + DP (n = 13)	0.40 mg/kg PQ + DP (n = 17)	DP only (n = 11)		0.25 mg/kg PQ + DP (n = 20)	
Age, y, median (IQR)	13.0 (12.0–16.0)	15.0 (11.0–20.0)	13.0 (11.0–29.0)		15.5 (12.5–20.0)	
Hemoglobin, g/dL, mean (SD)	13.1 (1.5)	12.8 (1.4)	12.9 (1.8)		13.5 (1.4)	
Asexual parasite prevalence by microscopy, % (n/N)	15.4 (2/13)	11.8 (2/17)	18.2 (2/11)		0.0 (0/20)	
Asexual parasite density by microscopy, parasites/μL^b^	1520; 2384	288; 320	400; 1136		-	
Gametocyte prevalence by microscopy, % (n/N)	0.0 (0/13)	0.0 (0/17)	9.1 (1/11)		0.0 (0/20)	
Gametocyte prevalence by qRT-PCR, % (n/N)	7.7 (1/13)	29.4 (5/17)	27.3 (3/11)		10.0 (2/20)	
CYP2D6 inferred poor metabolizer phenotype, % (n/N)	0.0 (0/13)	0.0 (0/15)	0.0 (0/11)		5.3 (1/19)	
CYP2D6 inferred intermediate metabolizer phenotype, % (n/N)	30.8 (4/13)	40.0 (6/15)	54.5 (6/11)		26.3 (5/19)	

Abbreviations: G6PD, glucose-6-phosphate dehydrogenase; PQ, primaquine; AL, artemether-lumefantrine; IQR, interquartile range; QT-NASBA, quantitative nucleic acid sequence-based amplification; SD, standard deviation; CYP2D6, cytochrome P-450 isoenzyme 2D6; DP, dihydroartemisinin-piperaquine; qRT-PCR, quantitative reverse transcriptase PCR.

CYP2D6 inferred poor metabolizer phenotype = activity score 0.0; CYP2D6 inferred intermediate metabolizer phenotype = activity score 0.5–1.0.

^a^ Potential *CYP2D7/CYP2D6* hybrid allele in 2 subjects.

^b^ Individual asexual parasite densities for parasite positive individuals only.

Genotyping of the 202A and 376G mutations in the G6PD gene broadly confirmed the phenotypic status of study participants (S1 Table). In Burkina Faso all individuals with the 202A mutation were G6PDd by fluorescent spot test; two lacked the 376G mutation. In The Gambia the 202A mutation was detected in 13/30 individuals who were phenotypically G6PDd whilst 14 had the 376G mutation only and 3 were wild-type for the 202A and 376G mutation (S1 Table). CYP2D6 metabolizer phenotypes were inferred from genotypes for all participants enrolled except for 2 who potentially had a *CYP2D7/2D6* hybrid allele with unknown metabolizer phenotype in Burkina Faso and 3 individuals with missing samples in The Gambia (Table 1, S2 Table). Two participants in the Gambia were also potential hybrid *CYP2D7/2D6* allele carriers, but one was homozygous for the *4-allele, which strongly suggests this person to be a poor metabolizer and was therefore classified as such, the other of the two could not have a CYP2D6 metabolizer phenotype inferred.

All participants from both study sites cleared their asexual parasites by day 2, based on microscopy results. The current trials with limited study population sizes were not designed to determine gametocyte clearance; larger efficacy trials have addressed this recently [11–14]. In Burkina Faso, gametocyte prevalence by QT-NASBA in the AL only group was 80% (8/10) on day 3 and 33.3% (2/6) on day 7, which was numerically higher than in individuals receiving 0.25 mg/kg PQ (69.0% [20/29; *P* = 0.50] on day 3 and 7.7% [2/26; *P* = 0.09] on day 7) or 0.40 mg/kg PQ (68.2% [15/22; *P* = 0.68] on day 3 and 7.1% [1/14; *P* = 0.20] on day 7). In The Gambia, gametocyte prevalence by QT-NASBA in the DP only group was 30.0% (3/10) on day 3 and 22.2% (2/9) on day 7, which was statistically significantly higher than in individuals receiving 0.25 mg/kg PQ (0% [0/20, *P* = 0.03] on day 3 and 0.0% [0/29; *P* = 0.05] on day 7) and non-significantly higher than in individuals receiving 0.40 mg/kg PQ (16.7% [2/12; *P* = 0.21] on day 3 and 0.0% [0/11; *P* = 0.19] on day 7).

### Hematological changes after treatment

**Fig 2:**
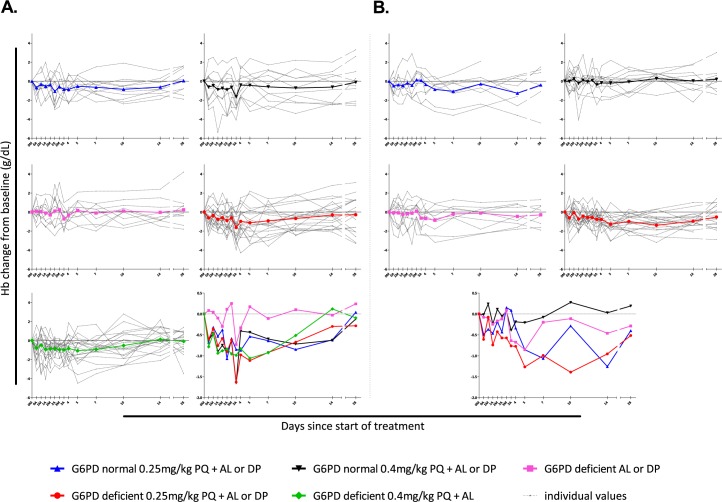
Hemoglobin levels during 28-day follow-up Burkina Faso (A) and The Gambia (B). Hemoglobin concentrations (g/dL) during follow-up are expressed relative to that at enrolment for each individual (grey dotted lines) and for each treatment group (colored lines). Abbreviations: 0M, day 0 morning; 0A, day 0 afternoon, etc.; Hb, hemoglobin; G6PD, glucose-6-phosphate dehydrogenase; PQ, primaquine; AL, artemether-lumefantrine; DP, dihydroartemisinin-piperaquine.

**Fig 3:**
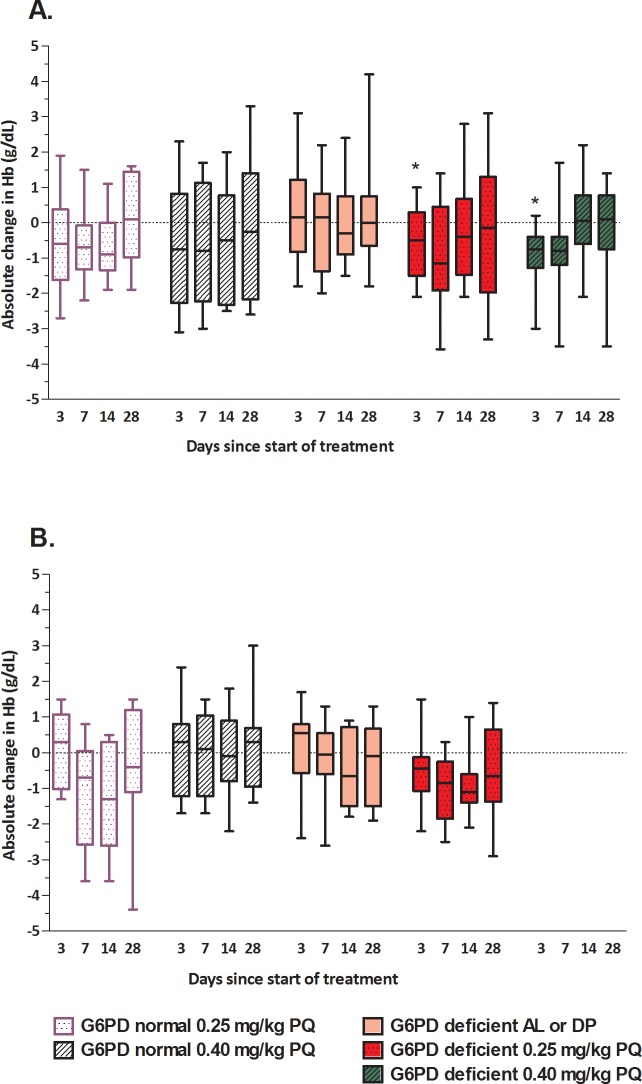
Absolute changes in hemoglobin levels during 28-day follow-up for Burkina Faso (A) and The Gambia (B). Hemoglobin concentrations (g/dL) on days 3, 7, 14 and 28 during follow-up are expressed relative to that at enrolment for each treatment group. Asterisks indicate a statistically significant reduction compared to baseline by repeated measures mixed models. Abbreviations: Hb, hemoglobin; G6PD, glucose-6-phosphate dehydrogenase; PQ, primaquine.

We first describe changes in hemoglobin relative to baseline values (Fig 2) and absolute changes in hemoglobin concentration (Fig 3) for each of the study arms and study settings separately. In all groups receiving PQ, mean hemoglobin concentrations, presented as relative or absolute change, declined in the first week after treatment and returned to baseline values during follow-up (Figs 2 and 3). Similarly, paired analysis of hemoglobin concentrations relative to baseline indicated reductions in G6PDd individuals in the first week after receiving 0.25mg/kg or 0.4mg/kg PQ (Fig 3). These reductions were statistically significant on day 3 after 0.25 mg/kg PQ (coefficient, -0.92; 95% CI, -1.81, -0.024; *P* = 0.044) and 0.40 mg/kg PQ (coefficient, -1.17; 95% CI, -2.06, -0.28; *P* = 0.010) in Burkina Faso. None of the control groups showed meaningful reductions in hemoglobin concentrations during follow-up (Figs 2 and 3). Analyses of hemoglobin as absolute value reflected the same patterns with an absolute decline in hemoglobin levels for G6PDd study participants one week after treatment with PQ, followed by a return to baseline values (Fig 3 and S3 Table).

Whilst mean changes in hemoglobin concentration allow a general comparison between groups, maximum reductions may better reflect severe hemolysis. We thus calculated the maximum reductions at absolute and relative hemoglobin values that were experienced by each trial participant and the proportion of participants experiencing a reduction >2.5 g/dL. The maximum reductions in hemoglobin concentrations, both as absolute and relative change, were strongly influenced by baseline hemoglobin concentrations with larger reductions for individuals with higher baseline concentrations (S1 Fig). Subsequent comparisons, even those on relative hemoglobin concentration, were therefore adjusted for baseline hemoglobin concentration [23]. In Burkina Faso, maximum drops in hemoglobin concentration (the largest reduction in hemoglobin compared to baseline at any time point during follow-up) after 0.25 mg/kg PQ were larger in G6PDd than G6PD-normal participants, both at absolute (mean difference, -0.64 g/dL; 95% CI, -1.31, 0.04; *P* = 0.062) and relative (mean difference, -4.23%; 95% CI, -9.17, 0.70; *P* = 0.09) scales (Fig 2 and S3A Table). Similarly, maximum drops in hemoglobin concentration in Burkina Faso were significantly larger in G6PDd than G6PD-normal participants after 0.40 mg/kg PQ, both at an absolute scale (mean difference, -0.82 g/dL; 95% CI, -1.51, -0.13; *P* = 0.022) and expressed as proportion of baseline hemoglobin concentration (mean difference, -6.23%; 95% CI, -11.22, -1.25; *P* = 0.016) (Fig 2 and S3A Table). In The Gambia, we observed no statistically significant difference in maximum drops in hemoglobin concentration between G6PDd and G6PD-normal participants treated with 0.25 mg/kg PQ at the absolute (*P* = 0.93) or relative scale (*P* = 0.95) (S3B Table). In Burkina Faso, the proportion of participants with a decrease in hemoglobin of >2.5 g/dL was 45.0% (9/20) in the G6PDd group receiving 0.25 mg/kg PQ and 40.0% (4/10) in the G6PD-normal group receiving the same PQ dose (*P* = 0.167). The proportion of participants with a decrease in hemoglobin of >2.5 g/dL was 35.0% (7/20) in the G6PDd group receiving 0.40 mg/kg PQ and 50.0% (5/10) in the G6PD-normal group receiving the same dose (*P* = 0.687). In The Gambia, the proportion of participants with a decrease in hemoglobin of >2.5 g/dL was 20.0% (4/20) in the G6PDd group receiving 0.25 mg/kg PQ and 33.3% (3/9) in the G6PD-normal group receiving the same dose (*P* = 0.34, S3A Table).

**Fig 4:**
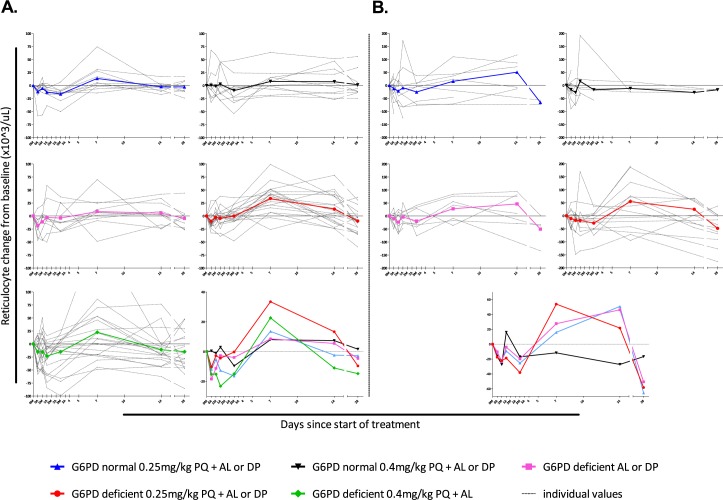
Absolute reticulocyte counts during 28-day follow-up for Burkina Faso (A) and The Gambia (B). Absolute reticulocyte counts (x10^3^/μL) during follow-up are expressed relative to that at enrolment for each individual (grey dotted lines) and for each treatment group (colored lines). The value not displayed in the G6PD-deficient 0.40 mg/kg PQ group was 216 x10^3^/μL. Abbreviations: 0M, day 0 morning; 0A, day 0 afternoon, etc.; G6PD, glucose-6-phosphate dehydrogenase; PQ, primaquine; AL, artemether-lumefantrine; DP, dihydroartemisinin-piperaquine.

**Fig 5:**
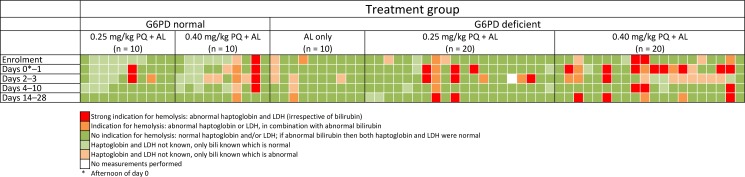
Haptoglobin, lactate dehydrogenase (LDH) and total bilirubin levels as parameters of hemolysis during follow-up in Burkina Faso. Each individual represents one vertical column. Abnormal levels were defined as: haptoglobin <0.3 g/L; LDH ≥250 U/L; total bilirubin ≥20.53 μmol/L. Abbreviations: G6PD, glucose-6-phosphate dehydrogenase; PQ, primaquine; AL, artemether-lumefantrine.

None of the study participants developed moderate or severe anemia. There was a general trend towards an increase in reticulocyte count at day 7 in all treatment arms in both Burkina Faso and The Gambia, particularly in G6PDd trial participants receiving PQ (Fig 4). Haptoglobin and LDH concentrations were determined in all available plasma samples for Burkina Faso only. Haptoglobin concentrations <0.3 g/L in combination with LDH levels >250 U/L were considered indicative of hemolysis [38, 39]. In addition, total bilirubin concentration (abnormal defined as ≥20.53 μmol/L) was used as indicator of hemolysis. Abnormal values for either haptoglobin or LDH, and total bilirubin were strongly correlated (*P* ≤ 0.008). The proportion of individuals with haptoglobin, LDH and/or total bilirubin concentrations indicative of hemolysis was higher in PQ-treated individuals (Fig 5). Genetically inferred CYP2D6 metabolizer status was not associated with absolute and relative reductions in hemoglobin concentration on day 7 (S4 Table).

### Adverse events and clinical assessment of hemolysis

There were 82 AEs recorded for the 70 participants from Burkina Faso who completed follow-up. All reported AEs were graded as mild or moderate (Table 2). In all 10 participants from Burkina Faso with hemoglobinuria, the diagnosis was made between the evening visit of day 0 (8 hours after enrolment and first treatment dose) and the morning visit of day 3 (72 hours after enrolment), with no difference between treatment groups (Table 2). In The Gambia, a total of 26 AEs were recorded in 20 participants and all were classified as mild. Sixteen participants had hemoglobinuria at enrollment (before the first dose of DP) that did not worsen during follow-up. There was no association between hemoglobinuria and randomization group. Microscopic urinalysis confirmed a diagnosis of schistosomiasis and participants were subsequently treated with praziquantel (Table 3). We recorded no SAEs, no cases of blackwater fever, and no red, black, or tea-colored urine. None of the participants required a blood transfusion.

**Table 2:** Adverse events of any severity in the different treatment groups in Burkina Faso.

	Treatment Group														
	G6PD Normal						G6PD Deficient								
	0.25 mg/kg PQ + AL (n = 10)			0.4 mg/kg PQ + AL (n = 10)			AL only (n = 10)			0.25mg/kg PQ + AL (n = 20)			0.4mg/kg PQ + AL (n = 20)		
Adverse Event	No. subjects^a^	No. AEs	Days after PQ	No. subjects	No. AEs	Days after PQ	No. subjects	No. AEs	Days after AL^b^	No. subjects	No. AEs	Days after PQ	No. subjects	No. AEs	Days after PQ
Abdominal pain													2	2	0, 0
Abscess										1	1	1			
Anorexia													1	1	0
Arthralgia	2	2	1, 2										3	4	2, 2, 4, 13
Bronchitis	1	1	1	1	2	4, 26				1	1	12	1	1	7
Cough										1	1	26			
Constipation													1	1	13
Dizziness				1	1	13							4	4	0, 3, 8, 9
Dolor at blood draw site													1	1	14
Drowsiness													1	1	1
Dysuria				2	2	8, 13									
Enteritis										1	1	3			
Fatigue				1	1	13	2	2	1, 1	1	1	27	1	1	3
Fever	1	1	1							1	1	0			
Gastralgia										1	1	12			
Hemoglobinuria^c^	1	1	2	2	2	0, 2	1	1	1	4	4	0, 2, 3, 3	2	4	0, 0, 28, 28
Headache				1	2	2, 13	2	2	2, 20	2	2	1, 13	6	6	1, 2, 2, 4, 5, 13
Hepatitis B													1	1	28
Low back pain													1	1	5
Myalgia	1	1	2										2	2	2, 4
Parasitosis	1	1	9	2	2	1, 13							1	1	6
Pharyngitis				1	1	14									
Pruritus				2	2	0, 13									
Rash				1	1	10									
Rhinitis				2	2	3, 5				2	2	3, 8	3	3	6, 13, 13
Superficial mycosis							1	1	1						
Tooth pain													1	1	4
Wound on thigh										1	1	21			
**Total no. subjects experiencing any AE, %**	**3 (30%)**	**7**		**9 (90%)**	**18**		**4 (40%)**	**6**		**10 (50%)**	**16**		**13 (65%)**	**35**	
**Total no. subjects experiencing possible/probable AEs, %**	**1 (10%)**	**1**		**4 (40%)**	**5**		**2 (20%)**	**3**		**5 (25%)**	**6**		**9 (45%)**	**18**	

Abbreviations: G6PD, glucose-6-phosphate dehydrogenase; PQ, primaquine; AL, artemether-lumefantrine; No., number; AE, adverse event.

^a^ Subjects could have more than one adverse event.

^b^ First dose of artemether-lumefantrine.

^c^ Hemoglobinuria was reported as adverse event in combination with a positive urine dipstick. All cases of hemoglobinuria were probably or possibly related to the trial, except for two cases occurring on day 28 in the G6PD-deficient group receiving 0.40 mg/kg PQ.

**Table 3:** Adverse events of any severity in the different treatment groups in The Gambia.

	Treatment Group											
	G6PD Normal						G6PD Deficient					
	0.25 mg/kg PQ + DP (n = 9)^a^			0.4 mg/kg PQ + DP (n = 11)^a^			DP only (n = 9)^a^			0.25mg/kg PQ + DP (n = 20)^a^		
Adverse Event	No. subjects^b^	No. AEs	Days after PQ	No. subjects	No. AEs	Days after PQ	No. subjects	No. AEs	Days after DP^c^	No. subjects	No. AEs	Days after PQ
Abdominal pain	1	1	9				1	1	5			
Chest pain	1	1	9							1	1	28
Hemoglobinuria^d^	3	3	0	3	3	0	1	1	0	7	7	0
Headache	2	2	2,9				2	2	13,14	2	2	5,8
Trauma				1	1	14						
Vomiting	1	1	9									
**Total no. subjects experiencing any AE, %**	**4 (44%)**	**8**		**3 (27%)**	**4**		**4 (44%)**	**4**		**9 (45%)**	**10**	
**Total no. subjects experiencing possible/probable AEs, %**	**0 (0%)**			**0 (0%)**			**0 (0%)**			**0 (0%)**		

Abbreviations: G6PD, glucose-6-phosphate dehydrogenase; PQ, primaquine; DP, dihydroartemisinin-piperaquine; No., number; AE, adverse event.

^a^ Individuals who completed follow-up

^b^ Subjects could have more than one adverse event.

^c^ First dose of dihydroartemisinin-piperaquine.

^d^ Hemoglobinuria was observed at enrollment (before PQ administration) and microscopic urinalysis confirmed a diagnosis of schistosomiasis. All participants were treated with praziquantel.

## Discussion

We present the results from two linked randomized controlled trials that evaluated the tolerability and safety of a single low-dose PQ in combination with AL or DP in G6PDd African males with asymptomatic *P*. *falciparum* malaria. Following PQ treatment, we observed transient reductions in hemoglobin concentrations in both G6PDd and G6PD-normal individuals. None of the study participants experienced SAEs or developed severe anemia.

Primaquine-associated hemolysis is dose-dependent and related to the degree of G6PD deficiency [23, 40–43]. In the current trials we specifically assessed the safety of single low-dose PQ in G6PDd individuals, with G6PD normal individuals receiving the same PQ dose as controls. In both Burkina Faso and The Gambia, G6PDd individuals had a statistically significant reduction in hemoglobin concentration after PQ treatment [16, 40–42]. Absolute and relative reductions in hemoglobin concentrations were most pronounced in the first week after treatment, and normalized during follow-up. None of the study participants developed moderate or severe anemia, experienced SAEs or required blood transfusion or hospitalization. Contrary to a study in Tanzania that observed no difference in hemoglobin concentrations between G6PDd and G6PD-normal individuals [25], we report a larger reduction in hemoglobin concentration in G6PDd individuals in Burkina Faso following treatment with 0.25 mg/kg and 0.40 mg/kg PQ. Whilst this association between G6PDd and hemoglobin reductions is in line with the majority of studies that measured hemoglobin after single dose PQ [16, 22–24], we did not observe a statistically significant difference between G6PDd and G6PD-normal individuals in The Gambia. In general, we observed considerable individual variation in hemoglobin dynamics during follow-up and a marked reduction in hemoglobin concentration in a proportion of G6PD-normal individuals who received PQ [16, 22–24]. In our study populations the fluctuations in hemoglobin concentrations during follow-up are probably influenced by malaria-associated hemolysis [44]. We excluded symptomatic malaria patients and imposed a conservative minimum hemoglobin concentration prior to treatment. Since symptomatic malaria patients and anemic individuals are likely to display different patterns in hemoglobin concentrations following treatment, our findings cannot be extrapolated to this population. We observed a strong association between baseline hemoglobin concentration and absolute or relative reductions in hemoglobin during follow up [23], probably reflecting the influence of the age distribution of the red blood cell population on the severity of drug-induced hemolysis [45].

Large inter- and intra-individual variation was also observed in reticulocyte counts without consistent differences between PQ-treated and non-treated individuals or between G6PDd and G6PD-normal individuals. Reticulocytosis is potentially an important marker for acute hemolysis and reticulocyte counts were elevated following various doses of PQ in an in vivo drug screening model using G6PDd mice [46]. We aimed to obtain further insights in hemolysis after PQ by measuring LDH, released from hemolysed red blood cells, and haptoglobin, which binds to hemoglobin released during intravascular or extravascular hemolysis. Whilst we performed these analyses post-hoc and only for individuals with remaining plasma samples from Burkina Faso, we noticed a trend towards more abnormal values on days 1–3 in G6PDd individuals, particularly in those receiving 0.40 mg/kg PQ. Although an elevated LDH is a non-specific marker of tissue damage and can be found in many conditions other than hemolysis, the combination of an increased serum LDH and a reduced haptoglobin is highly specific for diagnosing hemolysis [38, 39]. Our sparse observations on reticulocytes, LDH and haptoglobin support indications of transient hemolysis in the first week following PQ. Our findings suggest minimal safety concerns related to PQ at 0.25 mg/kg in G6PDd individuals, which is in line with other recent studies that used this PQ-dose [24, 25]. However, it is unclear how these findings can be extrapolated to individuals with lower starting hemoglobin concentrations, particularly children. Moreover, individuals with more severe forms of G6PD deficiency than the African A- variant may also experience more severe hemolysis following PQ [41, 47] and warrant safety studies in these populations. Severe hemolytic events may be rare [16, 43] and unlikely to be detected in small safety or efficacy studies. Incorporation of pharmacovigilance and *G6PD* genotyping into larger community based studies with PQ will help establish a solid base for broader implementation of single low-dose PQ.

Our study enrolled G6PDd individuals based on the phenotypic “Beutler’s” fluorescent spot test. The correlation between G6PD genotyping and phenotyping is typically mixed [48]. Compared to our study population in Burkina Faso, a large fraction of G6PDd study participants in The Gambia lacked the 202A mutation. This may be explained by the fact that G6PD deficiency alleles other than the 202A/376G G6PD A- allele are relatively common in The Gambia; alternative alleles include the 542T/376G Santamaria and the 968C/376G G6PD Betica-Selma allele [49]. There is increasing evidence for a role of CYP2D6 metabolizer status in determining the efficacy of PQ in preventing *P*. *vivax* relapses [30, 36]. CYP2D6 activity is hypothesized to be a rate-limiting step in the formation of active metabolite(s) of PQ for *P*. *vivax*. It is currently unclear whether CYP2D6 activity is also associated with PQ efficacy for *P*. *falciparum* gametocyte clearance or PQ-associated hemolysis. Our study was underpowered to detect such effects; our exploratory analysis showed no effect of genetically inferred CYP2D6 metabolizer status on PQ-associated reductions in hemoglobin concentrations.

Our findings on PQ safety fill an important gap in considerations on PQ implementation [27] and need to be considered in combination with evidence on PQ efficacy. The rationale for PQ treatment in *P*. *falciparum* is that it results in a community benefit in terms of reducing malaria transmission that outweighs potential individual-level risks associated with PQ use. Efficacy trials indeed demonstrate that the addition of PQ to ACTs results in a substantially reduced duration of gametocyte carriage [11–14]. The actual implications of PQ-associated gametocyte clearance in terms of reductions in the number of infected mosquitoes strongly depend on the study population and potentially on the type of ACT that is used in combination with PQ. In *P*. *falciparum* gametocyte carriers with high gametocyte densities and high transmission potential prior to treatment, 0.25 mg/kg PQ in combination with DP reduced transmission to mosquitoes by >90% within 48 hours after administration whilst a considerable proportion of DP-treated individuals remained infectious to mosquitoes for at least one week after treatment [13]. In asymptomatic parasite carriers with lower gametocyte densities prior to treatment, residual transmission after treatment with DP [14] and AL [12] may be considerably smaller and the added benefit of PQ may therefore be more modest [12, 14]. Decisions on the benefit of PQ for *P*. *falciparum* malaria control and elimination have to consider target populations and take into account that the proportion of infected individuals that can be covered with efficacious antimalarials is a major determinant of impact [50, 51]. Our findings on PQ safety form a relevant addition to these considerations and indicate that single low-dose PQ is associated with hemolysis post treatment but that this hemolysis is not severe and is self-limiting. Evidence is accumulating that single low-dose PQ can be used in Africa without prior G6PD screening.

## Supporting information

S1 Table G6PD phenotype in relation to genotype. (DOCX)

S2 Table Cytochrome P-450 isoenzyme 2D6 allele frequencies for successfully genotyped participants. (DOCX)

S3 Table Hemoglobin levels following treatment for individuals who completed follow-up in Burkina Faso and The Gambia. (DOCX)

S4 Table Exploratory analysis of the association between genetically inferred CYP2D6 metabolizer status and changes in hemoglobin concentration on day 7 after primaquine treatment. (DOCX)

S1 Fig Association between baseline hemoglobin concentration and changes in hemoglobin concentration during follow-up. The figure shows the association between baseline hemoglobin concentration and changes in the maximum absolute change in hemoglobin concentration during follow-up (left panel: Pearson *r* = -0.69; *P* < 0.0001); and changes in the maximum relative change in hemoglobin concentration during follow-up expressed as % of baseline values (Pearson *r* = -0.59; *P* < .0001).
(TIF)

S1 File Protocol—Burkina Faso. (PDF)

S2 File Protocol—The Gambia. (PDF)

S3 File Consort checklist. (DOC)

## Abbreviations

ACT: artemisinin-based combination therapy
AE: adverse event
AL: artemether-lumefantrine
AS: Activity Score
CI: confidence interval
CNV: copy number variation
CYP2D6: Cytochrome P-450 isoenzyme 2D6
DMID: Division of Microbiology and Infectious Diseases
DP: dihydroartemisinin-piperaquine
G6PD(d): glucose-6-phosphate dehydrogenase (deficient)
IM: intermediate metabolizer
LDH: lactate dehydrogenase
PM: poor metabolizer
PQ: primaquine
qRT-PCR: quantitative reverse-transcriptase polymerase chain reaction
QT-NASBA: quantitative nucleic acid sequence-based amplification
RDT: rapid diagnostic test
SAE: serious adverse event
SD: standard deviation
UM: ultra-rapid metabolizer
WHO: World Health Organization
