# Supplementary material for: Safety of single low-dose primaquine in glucose-6-phosphate dehydrogenase deficient falciparum-infected African males: Two open-label, randomized, safety trials
Source: PLoS One. 2018 Jan 11;13(1):e0190272. doi: 10.1371/journal.pone.0190272 (PMC5764271; doi:10.1371/journal.pone.0190272)
Supplement: S1 Table — (DOCX) [file pone.0190272.s001.docx]

## S1 Table. G6PD Phenotype in Relation to Genotype

|  | Burkina Faso Treatment Group^a^ | | | | |
| --- | --- | --- | --- | --- | --- |
|  | G6PD Normal Phenotype | | G6PD Deficient Phenotype | | |
| G6PD Genotype | 0.25 mg/kg PQ + AL  (n = 11) | 0.40 mg/kg PQ + AL  (n = 11) | AL only  (n = 10) | 0.25 mg/kg PQ + AL (n = 22) | 0.40 mg/kg PQ + AL  (n = 24) |
| A- (202A and 376G mutation) | 0 | 0 | 8 | 21 | 21 |
| A+ (376G mutation only) | 3 | 4 | 1 | 0 | 2 |
| Asahi (202A mutation only) | 0 | 0 | 1 | 1 | 0 |
| B (wild type) | 8 | 7 | 0 | 0 | 1 |
|  | The Gambia Treatment Group^a^ | | | | |
|  | G6PD Normal Phenotype | | G6PD Deficient Phenotype | | |
| G6PD Genotype | 0.25 mg/kg PQ + DP  (n = 13) | 0.40 mg/kg PQ + DP  (n = 15) | DP only  (n = 11) | 0.25 mg/kg PQ + DP (n = 19) |  |
| A- (202A and 376G mutation) | 0 | 0 | 4 | 9 |  |
| A+ (376G mutation only) | 4 | 5 | 5 | 9 |  |
| Asahi (202A mutation only) | 0 | 0 | 0 | 0 |  |
| B (wild type) | 9 | 10 | 2 | 1 |  |

Abbreviations: G6PD, glucose-6-phosphate dehydrogenase; PQ, primaquine; AL, artemether-lumefantrine; DP, dihydroartemisinin-piperaquine.

^a^ Based on fluorescent spot test.
