# Supplementary material for: Safety of single low-dose primaquine in glucose-6-phosphate dehydrogenase deficient falciparum-infected African males: Two open-label, randomized, safety trials
Source: PLoS One. 2018 Jan 11;13(1):e0190272. doi: 10.1371/journal.pone.0190272 (PMC5764271; doi:10.1371/journal.pone.0190272)
Supplement: S2 Table — (DOCX) [file pone.0190272.s002.docx]

## S2 Table. Cytochrome P-450 Isoenzyme 2D6 Allele Frequencies for Successfully Genotyped Participants

|  | | | | | | | | | | | | The Gambia (n = 56) | | | Burkina Faso (n = 76) | | |
| --- | --- | --- | --- | --- | --- | --- | --- | --- | --- | --- | --- | --- | --- | --- | --- | --- | --- |
| Haplotype | 100 | 1023 | 1846 | 1863-64 | 2850 | 2988 | 3183 | 4180 | Dup^a^ | Exon 9 | Enzyme  Activity | Het^b^ | Hom^c^ | Freq^d^ | Het^b^ | Hom^c^ | Freq^d^ |
| *1 | C | C | G | - | C | G | G | G | NO | YES | Normal | 17 | 3 | 0.21 | 25 | 3 | 0.20 |
| *1x2^b^ | C | C | G | - | C | G | G | G | YES | YES | Increased | 0 | 0 | 0.00 | 1 | 0 | 0.01 |
| *2 | C | C | G | - | T | G | G | C | NO | YES | Normal | 16 | 4 | 0.21 | 20 | 0 | 0.13 |
| *2x2^e^ | C | C | G | - | T | G | G | C | YES | YES | Increased | 6 | 0 | 0.05 | 1 | 0 | 0.01 |
| *4 | T | C | A | - | C | G | G | C | NO | YES | None | 3 | 0 | 0.03 | 2 | 0 | 0.01 |
| *4x2^e^ | T | C | A | - | C | G | G | C | YES | YES | None | 0 | 0 | 0.00 | 1 | 0 | 0.01 |
| *5 | - | - | - | - | - | - | - | - | NO | NO | None | 9 | 0 | 0.08 | 12 | 0 | 0.08 |
| *10 | T | C | G | - | C | G | G | C | NO | YES | Decreased | 5 | 0 | 0.04 | 4 | 0 | 0.03 |
| *17 | C | T | G | - | T | G | G | C | NO | YES | Decreased | 17 | 0 | 0.15 | 33 | 5 | 0.28 |
| *17x2^e^ | C | T | G | - | T | G | G | C | YES | YES | ? | 0 | 0 | 0.00 | 2 | 0 | 0.01 |
| *29 | C | C | G | - | T | G | A | C | NO | YES | Decreased | 19 | 1 | 0.19 | 24 | 1 | 0.17 |
| *29x2^e^ | C | C | G | - | T | G | A | C | YES | YES | ? | 1 | 0 | 0.01 | 0 | 0 | 0.00 |
| *36 | T | C | G | - | C | G | G | C | NO | NO | Negligible | 1 | 0 | 0.01 | 1 | 0 | 0.01 |
| *40 | C | T | G | TTTCGCCCCx2 | T | G | G | C | NO | YES | None | 1 | 0 | 0.01 | 8 | 0 | 0.05 |
| *41 | C | C | G | - | T | A | G | C | NO | YES | Decreased | 1 | 0 | 0.01 | 0 | 0 | 0.00 |

^a^ Duplication of *CYP2D6* gene allele; ^b^ Heterozygotes; ^c^ Homozygotes; ^d^ Allele frequency; ^e^ For duplications of alleles amplification curves were used to estimate which allele might be duplicated, but this method is approximate (1). Two participants in each population with potential hybrid alleles are not included.

## References

1. Fang H, Liu X, Ramirez J, Choudhury N, Kubo M, Im HK, et al. Establishment of CYP2D6 reference samples by multiple validated genotyping platforms. Pharmacogenomics J. 2014;14(6):564-72.
