# Supplementary material for: Safety of single low-dose primaquine in glucose-6-phosphate dehydrogenase deficient falciparum-infected African males: Two open-label, randomized, safety trials
Source: PLoS One. 2018 Jan 11;13(1):e0190272. doi: 10.1371/journal.pone.0190272 (PMC5764271; doi:10.1371/journal.pone.0190272)
Supplement: S3 Table — (DOCX) [file pone.0190272.s003.docx]

## S3A Table. Hemoglobin Levels Following Treatment for Individuals Who Completed Follow-Up in Burkina Faso

|  | Treatment Group | | | | |
| --- | --- | --- | --- | --- | --- |
|  | G6PD Normal | | G6PD Deficient | | |
|  | 0.25 mg/kg PQ + AL  (n = 11) | 0.40 mg/kg PQ + AL  (n = 11) | AL only  (n = 10) | 0.25 mg/kg PQ + AL (n = 22) | 0.40 mg/kg PQ + AL  (n = 24) |
| **Hemoglobin absolute change, g/dL** |  |  |  |  |  |
| Hemoglobin maximum reduction during follow-up, g/dL, mean (SD) | -2.02 (0.83) | -2.23 (1.94) | -1.28 (1.04) | -2.13 (1.37) | -2.29 (1.07) |
| Compared to G6PD normal receiving same PQ dose, g/dL, mean difference (95% CI) |  |  |  | -0.64 (-1.31–0.04) | -0.82 (-1.51– -0.13) |
| *P* Value |  |  |  | 0.062 | 0.022 |
| Day 3, hemoglobin change, g/dL, mean (SD) | -0.59 (1.36) | -0.65 (1.83) | 0.25 (1.39) | -0.62 (0.97) | -0.96 (0.84) |
| Compared to G6PD normal receiving same PQ dose, g/dL, mean difference (95% CI) |  |  |  | -0.46 (-1.15–0.23) | -0.91 (-1.63– -0.19) |
| *P* Value |  |  |  | 0.187 | 0.015 |
| Day 7, hemoglobin change, g/dL, mean (SD) | -0.64 (1.01) | -0.60 (1.70) | -0.11 (1.29) | -0.92 (1.43) | -0.93 (1.20) |
| Compared to G6PD normal receiving same PQ dose, g/dL, mean difference (95% CI) |  |  |  | -0.65 (-1.61–0.30) | -0.94 (-1.86– -0.02) |
| *P* Value |  |  |  | 0.171 | 0.045 |
| Day 14, hemoglobin change, g/dL, mean (SD) | -0.62 (0.91) | -0.63 (1.67) | -0.03 (1.20) | -0.30 (1.35) | 0.12 (1.09) |
| Compared to G6PD normal receiving same PQ dose, g/dL, mean difference (95% CI) |  |  |  | -0.16 (-0.90–0.58) | 0.31 (-0.64–1.26) |
| *P* Value |  |  |  | 0.655 | 0.511 |
| Day 28, hemoglobin change, g/dL, mean (SD) | 0.04 (1.27) | -0.12 (2.01) | 0.24 (1.64) | -0.29 (1.83) | -0.10 (1.13) |
| Compared to G6PD normal receiving same PQ dose, g/dL, mean difference (95% CI) |  |  |  | -0.83 (-2.02–0.36) | -0.65 (-1.55–0.26) |
| *P* Value |  |  |  | 0.17 | 0.16 |
| **Hemoglobin relative change, %** |  |  |  |  |  |
| Hemoglobin maximum reduction during follow-up, %, mean (SD) | -13.46 (4.86) | -13.91 (11.89) | -9.08 (7.55) | -14.71 (8.96) | -15.96 (6.54) |
| Compared to G6PD normal receiving same PQ dose, %, mean difference (95% CI) |  |  |  | -4.23 (-9.17–0.70) | -6.23 (-11.22– -1.25) |
| *P* Value |  |  |  | 0.090 | 0.016 |
| Day 3, hemoglobin change, %, mean (SD) | -3.42 (9.12) | -3.31 (12.14) | 2.29 (10.62) | -4.08 (6.88) | -6.58 (5.51) |
| Compared to G6PD normal receiving same PQ dose, g/dL, mean difference (95% CI) |  |  |  | -3.43 (-8.40–1.53) | -7.01 (-12.10– -1.92) |
| *P* Value |  |  |  | 0.167 | 0.009 |
| Day 7, hemoglobin change, %, mean (SD) | -4.05 (6.89) | -3.11 (11.15) | -0.35 (9.68) | -6.15 (10.34) | -6.37 (7.75) |
| Compared to G6PD normal receiving same PQ dose, g/dL, mean difference (95% CI) |  |  |  | -4.56 (-11.53–2.42) | -6.98 (-13.24– -0.72) |
| *P* Value |  |  |  | 0.192 | 0.030 |
| Day 14, hemoglobin change, %, mean (SD) | -3.87 (6.06) | -3.27 (10.99) | 0.24 (9.26) | -1.51 (10.30) | 0.96 (7.39) |
| Compared to G6PD normal receiving same PQ dose, g/dL, mean difference (95% CI) |  |  |  | -1.07 (-6.84–4.70) | 1.25 (-5.08–7.58) |
| *P* Value |  |  |  | 0.707 | 0.689 |
| Day 28, hemoglobin change, %, mean (SD) | 0.69 (8.68) | 0.07 (13.94) | 2.32 (12.78) | -1.40 (13.45) | -0.22 (7.51) |
| Compared to G6PD normal receiving same PQ dose, g/dL, mean difference (95% CI) |  |  |  | -5.71 (-14.37–2.94) | -4.79 (-11.06–1.48) |
| *P* Value |  |  |  | 0.187 | 0.128 |
| **Decrease in hemoglobin >2.5 g/dL, % (n/N)** | 40.0 (4/10) | 50.0 (5/10) | 0.0 (0/10) | 45.0 (9/20) | 35.0 (7/20) |
| Compared to G6PD normal receiving same PQ dose, *P* Value |  |  |  | 0.167 | 0.687 |

Abbreviations: G6PD, glucose-6-phosphate dehydrogenase; PQ, primaquine; AL, artemether-lumefantrine; SD, standard deviation.

## S3B Table. Hemoglobin Levels Following Treatment for Individuals Who Completed Follow-Up in The Gambia

|  | Treatment Group | | | |
| --- | --- | --- | --- | --- |
|  | G6PD Normal | | G6PD Deficient | |
|  | 0.25 mg/kg PQ + DP  (n = 9) | 0.40 mg/kg PQ +DP  (n = 11) | DP only  (n = 9) | 0.25 mg/kg PQ + DP (n = 20) |
| **Hemoglobin absolute change, g/dL** |  |  |  |  |
| Hemoglobin maximum reduction during follow-up, g/dL, mean (SD) | -1.77 (1.48) | -1.21 (0.74) | -1.22 (1.30) | -1.83 (0.78) |
| Compared to G6PD normal receiving same PQ dose, g/dL, mean difference (95% CI) |  |  |  | 0.031 (-0.71–0.77) |
| *P* Value |  |  |  | 0.93 |
| Day 3, hemoglobin change, g/dL, mean (SD) | 0.15 (1.09) | 0.07 (1.28) | 0.09 (1.16) | -0.58 (0.87) |
| Compared to G6PD normal receiving same PQ dose, g/dL, mean difference (95% CI) |  |  |  | -0.65 (-1.43–1.23) |
| *P* Value |  |  |  | 0.096 |
| Day 7, hemoglobin change, g/dL, mean (SD) | -1.06 (1.55) | -0.08 (1.17) | -0.20 (1.17) | -0.99 (0.85) |
| Compared to G6PD normal receiving same PQ dose, g/dL, mean difference (95% CI) |  |  |  | 0.097 (-0.92–1.11) |
| *P* Value |  |  |  | 0.85 |
| Day 14, hemoglobin change, g/dL, mean (SD) | -1.26 (1.50) | 0.027 (1.18) | -0.46 (1.08) | -0.96 (0.74) |
| Compared to G6PD normal receiving same PQ dose, g/dL, mean difference (95% CI) |  |  |  | 0.055 (-0.81–0.92) |
| *P* Value |  |  |  | 0.90 |
| Day 28, hemoglobin change, g/dL, mean (SD) | -0.40 (2.01) | 1.19 (1.31) | -0.29 (1.18) | -0.52 (1.18) |
| Compared to G6PD normal receiving same PQ dose, g/dL, mean difference (95% CI) |  |  |  | -0.16 (-1.37–1.05) |
| *P* Value |  |  |  | 0.79 |
| **Hemoglobin relative change, %** |  |  |  |  |
| Hemoglobin maximum reduction during follow-up, %, mean (SD) | -12.77 (9.94) | -9.19 (6.01) | -8.48 (9.18) | -13.41 (5.07) |
| Compared to G6PD normal receiving same PQ dose, %, mean difference (95% CI) |  |  |  | 0.15 (-5.41–5.11) |
| *P* Value |  |  |  | 0.95 |
| Day 3, hemoglobin change, %, mean (SD) | 1.69 (8.51) | 0.99 (9.83) | 1.34 (9.44) | -4.14 (6.46) |
| Compared to G6PD normal receiving same PQ dose, g/dL, mean difference (95% CI) |  |  |  | -5.29 (-11.17–0.58) |
| *P* Value |  |  |  |  |
| Day 7, hemoglobin change, %, mean (SD) | -7.45 (11.04) | -0.58 (9.33) | -1.15 (9.63) | -7.49 (6.14) |
| Compared to G6PD normal receiving same PQ dose, g/dL, mean difference (95% CI) |  |  |  | 0.10 (-7.24–7.45) |
| *P* Value |  |  |  | 0.98 |
| Day 14, hemoglobin change, %, mean (SD) | -8.69 (10.37) | 0.26 (9.46) | -2.96 (8.24) | -7.06 (5.49) |
| Compared to G6PD normal receiving same PQ dose, g/dL, mean difference (95% CI) |  |  |  | 0.15 (-6.30–6.60) |
| *P* Value |  |  |  | 0.96 |
| Day 28, hemoglobin change, %, mean (SD) | -2.24 (13.60) | 2.26 (10.96) | -1.38 (9.05) | -3.50 (8.87) |
| Compared to G6PD normal receiving same PQ dose, g/dL, mean difference (95% CI) |  |  |  | -1.57 (-10.23–7.09) |
| *P* Value |  |  |  | 0.71 |
| **Decrease in hemoglobin >2.5 g/dL, % (n/N)** | 33.3 (3/9) | 0.0 (0/11) | 22.2 (2/9) | 20.0 (4/20) |
| Compared to G6PD normal receiving same PQ dose, *P* Value |  |  |  | 0.34 |

Abbreviations: G6PD, glucose-6-phosphate dehydrogenase; PQ, primaquine; DP, dihydroartemisinin-piperaquine; SD, standard deviation.
