# Supplementary material for: Safety of single low-dose primaquine in glucose-6-phosphate dehydrogenase deficient falciparum-infected African males: Two open-label, randomized, safety trials
Source: PLoS One. 2018 Jan 11;13(1):e0190272. doi: 10.1371/journal.pone.0190272 (PMC5764271; doi:10.1371/journal.pone.0190272)
Supplement: S4 Table — (DOCX) [file pone.0190272.s004.docx]

## S4 Table. Exploratory Analysis of the Association Between Genetically Inferred CYP2D6 Metabolizer Status and Changes in Hemoglobin Concentration on Day 7 After Primaquine Treatment.

|  | Burkina Faso | |
| --- | --- | --- |
|  | Absolute change in hemoglobin concentration on day 7 after PQ treatment relative to baseline (g/dL) | Relative change in hemoglobin concentration on day 7 after PQ treatment relative to baseline (%) |
|  | Β (se β), *P* value | Β (se β), *P* value |
| G6PDd^a^ | -0.82 (33.3), *P* = 0.017 | -6.01 (2.37), *P* = 0.014 |
| Baseline hemoglobin concentration, g/dL | -0.57 (0.11), *P* < 0.001 | -3.6 (0.81), *P* < 0.001 |
| CYP2D6 poor/intermediate^b^ | 0.013 (0.30), *P* = 0.97 | 0.22 (2.14), *P* = 0.92 |
|  | The Gambia | |
|  | Absolute change in hemoglobin concentration on day 7 after PQ treatment relative to baseline (g/dL) | Relative change in hemoglobin concentration on day 7 after PQ treatment relative to baseline (%) |
|  | Β (se β), *P* value | Β (se β), *P* value |
| G6PDd^a^ | -0.47 (0.43), *P* = 0.29 | -3.97 (3.2), *P* = 0.23 |
| Baseline hemoglobin concentration, g/dL | -0.12 (0.14), *P* = 0.39 | -0.74 (1.06), *P* = 0.49 |
| CYP2D6 poor/intermediate^b^ | -0.24 (0.48), *P* = 0.62 | -2.13 (3.56), *P* = 0.62 |

Abbreviations: PQ, primaquine; CYP2D6, Cytochrome P-450 isoenzyme 2D6.

^a^ Phenotypically determined G6PD deficiency; G6PD-normal individuals serve as reference group.

^b^ Genetically inferred CYP2D6 metabolizer status; extensive/ultra rapid metabolizers serve as reference group.
