# Supplementary figures and images for: Safety of single low-dose primaquine in glucose-6-phosphate dehydrogenase deficient falciparum-infected African males: Two open-label, randomized, safety trials
Source: PLoS One. 2018 Jan 11;13(1):e0190272. doi: 10.1371/journal.pone.0190272 (PMC5764271; doi:10.1371/journal.pone.0190272)

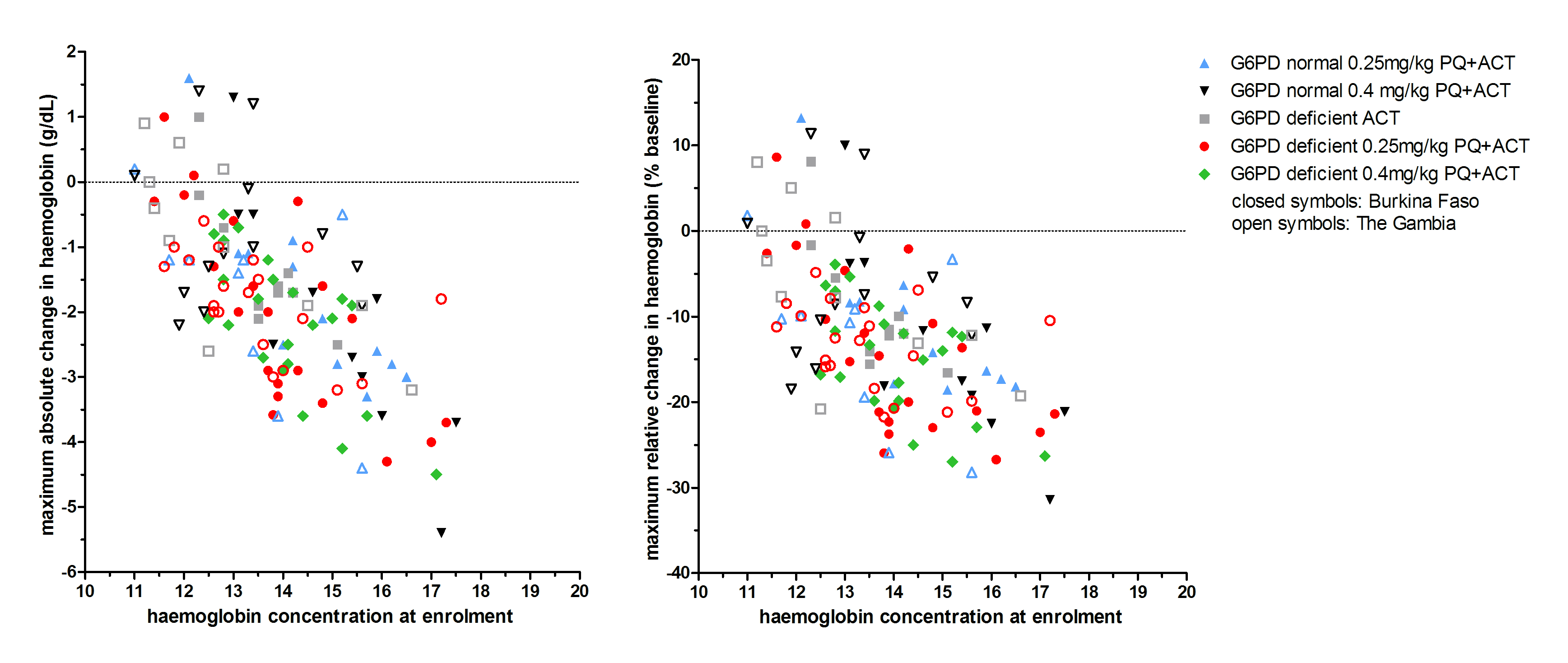

Supplement: S1 Fig — The figure shows the association between baseline hemoglobin concentration and changes in the maximum absolute change in hemoglobin concentration during follow-up (left panel: Pearson r = -0.69; P < 0.0001); and changes in the maximum relative change in hemoglobin concentration during follow-up expressed as % of baseline values (Pearson r = -0.59; P < .0001). (TIF) [file pone.0190272.s005.tif]
