# Supplementary material for: Safety of single low-dose primaquine in glucose-6-phosphate dehydrogenase deficient falciparum-infected African males: Two open-label, randomized, safety trials
Source: PLoS One. 2018 Jan 11;13(1):e0190272. doi: 10.1371/journal.pone.0190272 (PMC5764271; doi:10.1371/journal.pone.0190272)
Supplement: S1 File — (PDF) [file pone.0190272.s006.pdf]

1. TITLE OF THE PROJECT

Evaluation of the safety of primaquine in combination with artemether-lumefantrine in G6PD deficient males with an asymptomatic malaria infection in Burkina Faso (SAFEPRIM)

CONTENTS

1. TITLE OF THE PROJECT 1
2. SPONSOR AND REVIEWING ETHICS COMMITTEES 3
3. INVESTIGATORS AND INSTITUTIONAL AFFILIATIONS 4
4. ABSTRACT 5
5. LIST & DEFINITIONS OF ABBREVIATIONS, ACRONYMS 6
6. INTRODUCTION/BACKGROUND 7
6.1. History of primaquine use and safety in G6PD deficiency 8
6.2. G6PD deficiency worldwide and in Burkina Faso 9
6.3. Side effects of primaquine in G6PD deficient individuals 11
6.4. Assessing a safe and effective dose of PQ – data from Uganda 12
7. JUSTIFICATION OF THE STUDY 14
8. MAIN OBJECTIVE 14
9. SPECIFIC OBJECTIVES AND HYPOTHESES TO BE TESTED 14
10. DESIGN AND METHODOLOGY 15
10.1. General study design 15
10.2. Outcome measures 16
10.3. Study site and population 17
10.4. Participant screening and recruitment 17
10.5. Enrolment 18
10.6. Dose escalation protocol 18
10.7. Treatment and administration procedures 20
10.8. Follow-up and samples taken 20
10.9. Parasite detection 21
10.10. Haematology and biochemistry 22
10.11. G6PD deficiency testing 22
10.12 Pharmacological analysis 22
11. SAMPLE SIZE CONSIDERATIONS 25
12. DATA ANALYSIS PLAN 25

13. ETHICAL CONSIDERATIONS 25
13.1. Confidentiality 26
13.2. Participant reimbursement 26
13.3. Good clinical practice 26
14. SAFETY MANAGEMENT 27
14.1. Safety management 27
14.1.1. Withdrawal of individual subjects 27
14.1.2. Premature termination of the study 27
14.2. Management of haemolysis 27
14.2.1. Measures of haemolysis 28
14.3. Safety reporting 30
14.3.1. Adverse events 30
14.3.2. Serious adverse events 31
14.3.3. Adverse Event Data Collection 31
14.3.4. Assessment of causality 32
14.4. Safety follow-up 33
13.4.1 Follow-up of subjects that voluntarily withdraw participation 33
14.4.2. Follow-up of adverse events 33
14.4.3. Follow-up of serious adverse events 34
14.5. LSM and Data Safety Monitoring Board (DSMB) 34
14.6. Data monitoring 35
15. TIME FRAME AND DURATION OF THE PROJECT 35
16. REFERENCES 36
17. ROLES INVESTIGATORS 38

2. SPONSOR AND REVIEWING ETHICS COMMITTEES

Sponsor:

London School of Hygiene and Tropical Medicine

Keppel Street, London WC1E 7HT

Tel +44 (0) 20 7927 2626

Fax +44 (0) 20 75805636

e-mail: patricia.henley@lshtm.ac.uk

Reviewing ethics committees:

- London School of Hygiene and Tropical Medicine
  Keppel Street, London WC1E 7HT
  Tel +44 (0) 20 7927 2626
  Fax +44 (0) 20 75805636
  e-mail: ethics@lshtm.ac.uk

- Comité d'Ethique pour la Recherche en Santé:
  Ministère de la Santé du Burkina Faso
  03 BP: 7009 Ouagadougou 03
  Tel : +22650326284

- Comité Institutionnel de Bioéthique
  Centre National de Recherche et de Formation
  Sur le Paludisme (CNRFP)
  01 BP: 2208 Ouagadougou 01
  Tel : +22650324695

3. INVESTIGATORS AND INSTITUTIONAL AFFILIATIONS

Sodiomon Sirima, MD PhD[1] Local Principal Investigator

Guido Bastiaens, MD[2]

Chi Eziefula, MD[3]

Alfred Tiono, MD PhD[1]

Bronner Gonçalves, MD[3]

Teun Bousema, PhD[2,3] International Principal Investigator

Chris Drakeley, PhD[3] International Principal Investigator

[1] Centre National de Recherche et de Formation sur le Paludisme, Ouagadougou, Burkina Faso

[2] Radboud University Nijmegen Medical Centre, Nijmegen, the Netherlands

[3] London School of Hygiene and Tropical Medicine, London, UK

4. ABSTRACT

Primaquine (PQ) is currently the only available drug that can clear mature transmission stages of *P. falciparum* parasites. PQ was previously shown to clear gametocytes that persist after artemisinin-combination therapy (ACT) and it may therefore play a role in malaria elimination campaigns by preventing malaria transmission. However, there are safety concerns about the use of the previously World Health Organization (WHO)-recommended dose of 0.75 mg/kg in individuals who are glucose-6-phosphate dehydrogenase (G6PD) deficient. This has led to a new WHO recommendation of 0.25mg/kg. PQ at 0.75mg/kg may cause transient haemolysis in G6PD deficient individuals; this side effect is dose dependent. Recently, we have shown that a 0.4 mg/kg PQ dose may as effectively reduce gametocyte carriage as the WHO previously recommended dose of 0.75 mg/kg. The new low dose recommendation of 0.25mg/kg is thought to maintain efficacy and have a significantly reduced haemolytic effect.

In the current study we aim to assess safety of PQ, in particular the risk of acute haemolysis, when given together with artemether-lumefantrine (AL) in G6PD deficient individuals. Forty male participants with asymptomatic malaria, normal haemoglobin levels (≥11g/dL), reduced G6PD enzyme function, and aged between 18 and 45 years will be sequentially enrolled into two dosing cohorts consisting of 20 individuals and doses of 0.25 and 0.4 mg/kg PQ in combination with a full three-day course of AL. Participants will first be assigned to the lowest open cohort; enrolment in the next cohort is initiated after tolerability and short-term safety is demonstrated at the preceding lower dose. Furthermore, we will include 3 control groups into the first cohort: i) 10 male participants aged 18-45 years with asymptomatic malaria, normal haemoglobin levels (≥11g/dL) and a reduced G6PD enzyme function will receive AL only, ii) 10 male participants with asymptomatic malaria, normal haemoglobin levels (≥11g/dL) and normal G6PD enzyme function will receive 0.25 mg/kg PQ in combination with a full three-day course of AL, and iii) 10 male participants with asymptomatic malaria, normal haemoglobin levels (≥11g/dL) and normal G6PD enzyme function will receive 0.4 mg/kg PQ with AL. Clinical follow-up of participants and sampling will be done twice daily for the first 4 days (days 0, 1, 2 and 3) and once daily on days 4, 5, 7, 10, 14 and 28. At each time-point laboratory safety parameters are measured, including haematology, biochemistry, urine dipstick for haemoglobinuria/urobilinogen and parasite density. Five individuals from each intervention group (total of 10) will also be asked to provide seven venous blood samples on days 0, 1 and 2 to study pharmokinetics of PQ in G6PD deficient individuals.

5. LIST & DEFINITIONS OF ABBREVIATIONS, ACRONYMS

ACT	artemisinin-based combination therapy
AE	adverse event
AL	artemether-lumefantrine
C_{max}	peak plasma drug concentration
CI	confidence interval
CNRFP	Centre National de Recherche et Formation sur le Paludisme
CRF	case record form
D0, D1, D2	day 0, day 1, day 2 of study medication administration
DSMB	data safety and monitoring board
EIR	entomological inoculation rate
G6PD	glucose-6-phosphate dehydrogenase
GCP	good clinical practice
GCT	gametocyte clearance time
GMR	geometric mean ratio
Hb	haemoglobin
IRB	institutional review board
MDA	mass drug administration
LSHTM	London School of Hygiene and Tropical Medicine, London, UK
LSM	local safety monitor
PQ	primaquine
PQ1, PQ2	test doses of primaquine
QT-NASBA	real-time quantitative nucleic acid sequence-based amplification
RUNMC	Radboud University Nijmegen Medical Centre, Nijmegen, the Netherlands
SAE	serious adverse event
SD	standard deviation
SP	sulphadoxine-pyrimethamine
T_{max}	time to reach peak plasma drug concentration
WHO	World Health Organization

6. INTRODUCTION/BACKGROUND

A new global effort is underway to step up malaria control and push towards the elimination of malaria as a public health problem [1]. Some substantial successes have been achieved in shrinking the global distribution of malaria. In Africa, effective elimination programmes have been initiated in Zanzibar and South Africa. There are now several African countries with a commitment to malaria elimination. This call for elimination has created a drive for the deployment of tools to reduce malaria transmission. One such tool is primaquine (PQ). It is a drug which can efficiently block the transmission of *Plasmodium falciparum* malaria from humans to mosquitoes by clearing the parasite's transmission stages, gametocytes that persist after artemisinin-combination therapy (ACT) [2, 3]. A study conducted in Tanzania in symptomatic parasitized children demonstrated a dramatic reduction of gametocyte circulation time from 28.6 days with ACT alone to 6.3 days with ACT-PQ [4]. A drawback of using PQ is, however, that is has been shown to cause haemolysis in glucose-6-phosphate dehydrogenase (G6PD) deficient individuals [5-7]. G6PD is an essential enzyme linked to the pentose phosphate pathway; in erythrocytes, defence against oxidative stress is dependent on G6PD since they do not contain mitochondria. Its X-linked deficiency is one of world's most common genetic polymorphisms affecting 400 million people worldwide, mostly in *P. falciparum* malaria endemic areas [8].

A recent study in G6PD-sufficient individuals in Uganda found that lower doses of PQ than the then WHO standard dose of 0.75 mg/kg, retain efficacy for gametocyte clearance [Eziefula *et al,* under review]. In 2012, the WHO recommended that "A single 0.25 mg base/kg primaquine dose should be given to all patients with parasitologically-confirmed *P. falciparum* malaria on the first day of treatment in addition to an ACT, except for pregnant women and infants <1 year of age" (from Malaria Policy Advisory Committee Meeting 2012)[9]. However, detailed data about the lowest effective PQ dose and robust data regarding the safety of various PQ dosages in glucose-6-phosphate dehydrogenase (G6PD) deficient individuals are lacking [10]. This is remarkable, since all parasitologically-confirmed *P. falciparum* malaria patients in malaria elimination areas will now receive a single dose of PQ on the first day of treatment with ACT. This includes G6PD deficient individuals, who can account up to 30% of the population in some malaria-endemic areas in Africa with a mean prevalence of approximately 10% [8, 11]. Determining the safety profile of PQ in G6PD deficient individuals has become crucial and high quality clinical studies in G6PD deficient individuals are urgently needed.

6.1. History of primaquine use and safety in G6PD deficiency

Over the last fifty years many people have received PQ. Particularly in the 1960s when malaria eradication seemed feasible, entire regions and millions of people were exposed to repeated mass drug administration (MDA) with regimens containing PQ without individual G6PD deficiency screening. In 1973, the WHO recommended a single dose of PQ (0.75 mg/kg) for malaria transmission-blocking and considered prior screening for G6PD deficiency unnecessary [12]. Although little detailed information is available on the safety of these practices [13, 14], there are no reports of major toxic effects when given weekly or in single doses (usually 0.75 mg base/kg PQ) [14-18].

Safety data for PQ use in Africa or African Americans are limited. Burgess and Bray comment that PQ was “well-tolerated” [19]. In a large scale MDA conducted by Clyde in 1962, PQ was paired with amodiaquine (AQ) in a hyperendemic area of Tanzania. More than 15,000 subjects were studied in three clusters: weekly administration, fortnightly administration, and monthly administration. Clyde reported no safety data and it is unclear who was excluded from treatment [16]. In a series of studies in G6PD deficient African-American volunteers, Alving *et al.* showed that, in three individuals, haemolysis occurred with daily administration of 30mg (approximately 0.5 mg/kg) of PQ. But, after three weeks, the haematocrit levels recovered and lower doses of PQ resulted in less haemolysis. Eight weekly doses of 60 mg and 45 mg were not associated with haemolysis [20, 21]. Daily administration of 30mg of PQ to African Americans resulted in significant haemolysis in 1%, compared to no severe haemolysis when 15 mg was administered [22]. Tolerance in a pregnant woman (28 weeks gestation) has only been reported by Burgess and Bray, but there was no documentation of birth outcomes [19]. In a more recent study, Kenyan school children were randomized to receive 15mg PQ daily or three times a week as a malaria prophylactic. It is not clear whether G6PD deficient individuals were included and haemoglobin (Hb) levels are not reported but again the authors note simply that “PQ was remarkably well tolerated in our studies” [23]. In nearly 1500 patients studied prospectively in trials of single dose PQ given as a gametocytocide in Africa, Asia and South-America, no severe haemolysis requiring blood transfusion was reported [3, 9, 24-29].

Based on these previously mentioned observations, the risk of haemolysis with single-dose PQ was regarded as acceptable except in areas where severe G6PD deficiency is prevalent. Shekalaghe *et al.* showed, however, that administration of a single dose of 0.75 mg/kg PQ with sulphadoxine-pyrimethamine (SP) plus artesunate (AS) treatment in 1110 individuals older than 1 year caused moderate anaemia haemolysis in 40% (6/15) of G6PD deficient individuals (A- genotype, Class III variant, also prevalent in Burkina Faso, see paragraph 6.2) with mean change in Hb compared to

baseline of -2.5 g/dL, but in only 4.5% (18/399) of non-deficient individuals (mean change in Hb compared to baseline of -0.5 g/dL). There was no clinical compromise due to anaemia in any of the children, except in one child in the PQ arm, whose Hb dropped from 8.3 g/dL to 4.8 g/dL. In all cases haemolysis was transient, recovering by day 14 after treatment [26].

These recent findings illustrate the lack of robust data regarding the safety of PQ administration in G6PD deficient individuals. The current WHO recommendation for PQ (single dose of 0.25 mg base/kg) is based on safety concerns that were reported in most detail by a single study using 0.75mg/kg [26] and efficacy results of lower doses of PQ in historical studies that do not all meet current standards for randomized clinical trials [9, 31]. A recent dose-finding trial concluded that a reduced dose of 0.4mg/kg (but not of 0.1mg/kg) is likely to be as efficacious as the 0.75mg/kg dose [Eziefula *et al.*, Lancet Infect Dis *in press*] but there is no robust efficacy data on the newly recommended 0.25mg/kg dose.

6.2. G6PD deficiency worldwide and in Burkina Faso

Haemolysis after PQ is strongly associated with G6PD deficiency, which is expressed in males carrying a variant gene that may result in sufficient enzyme deficiency to lead to symptoms. The mean red blood cell enzyme activity in heterozygous females may be normal, moderately reduced, or grossly deficient depending upon the degree of lyonization and the degree to which the abnormal G6PD variant is expressed [32]. A heterozygous female with 50 percent normal G6PD activity, due to inactivation of one X chromosome in each cell via lyonization, has 50 percent normal red cells and 50 percent G6PD-deficient red cells. The deficient cells are as vulnerable to haemolysis as the enzyme-deficient red blood cells in males.

G6PD enzyme function varies widely in different regions as a consequence of different mutations underlying G6PD deficiency. The range of mutant alleles (over 140 have been characterized) result in varying degrees of deficiency of this enzyme. This reduction in enzyme function leaves red cells with lower amounts of NADPH (reduced nicotinamide adenine dinucleotide phosphate) with the result that they are susceptible to oxidative stress and consequently to haemolysis. PQ exposure leads to transient, dose-dependent intravascular haemolysis in individuals carrying mutant alleles. The severity of the haemolysis depends on the degree of enzyme deficiency and PQ dosing [9].

The WHO has classified different G6PD variants according to the magnitude of the enzyme deficiency and the severity of haemolysis [11]. Classes IV and V are of no clinical significance.

- Class I variants have severe enzyme deficiency (less than 10 percent of normal enzyme activity) and have chronic (nonspherocytic) hemolytic anemia.

- Class II variants, such as G6PD Mediterranean, also have severe enzyme deficiency, but there are usually only intermittent episodes of acute haemolysis associated with infection, drugs, or chemicals.
- Class III variants, such as G6PD A-, have mild to moderate enzyme deficiency (10 to 60 percent of normal enzyme activity) with intermittent episodes of acute haemolysis usually associated with infection, drugs, or chemicals.
- Class IV variants have no enzyme deficiency or haemolysis.
- Class V variants have increased enzyme activity.

The most common G6PD variant in Burkina Faso, and Africa as a whole, is the Class III A- variant [33, 34]. In 352 samples from clinical malaria patients from around Ouagadougou, all G6PD A- mutations involved the A376G/G202A mutation [34]. The G6PD A- variant encoded by these mutations has up to 80% enzyme function compared to wild type. This variant is associated with mild to moderate haemolysis in the presence of stimuli such as PQ. By contrast, some Southeast Asian and Mediterranean variants are Class I and II variants, being associated with severe deficiency, and PQ may provoke a severe haemolysis in these G6PD deficient individuals.

Table 1. G6PD variants, geography and broad risk of haemolysis

G6PD variant	Geographic region	Risk/ severity of haemolysis
B (Wild type)	Worldwide	None
A	Africa	Mild
A-	Africa<br>South America	Mild-moderate
Mediterranean	Middle East, Europe, South Asia	Severe
Viangchan, Mahidol, Vanua Lava, Canton, Alant, Kaiping	Southeast Asia, Australasia	Mild-moderate-severe
Seattle	Mediterranean, Western Europe, North Africa	Mild-moderate
Union	Mediterranean, Western Europe, North Africa, China, Pacific Islands	Moderate-severe

6.3. Side effects of primaquine in G6PD deficient individuals

Given its widespread use over the last fifty years, side effects of PQ are well known. The main side effects are as follows:

i) Gastro-intestinal symptoms if not given with food
ii) Methaemoglobinaemia (although there are no reports in the literature of individuals who required treatment for methaemoglobinemia due to PQ [35])
iii) Transient haemolysis in individuals with a predisposition, such as G6PD deficiency

Haemolysis occurs mostly in senescent erythrocytes as these cells become increasingly G6PD deficient, and therefore, more depleted in reduced glutathione, which is their principal antioxidant defence [8]. Therefore, reticulocytosis (proliferation of young red blood cells) in acute symptomatic malaria affords some protection, as the population of red cells is relatively younger.
The haemolytic side effect of PQ is dose-related and in proportion to the degree of G6PD deficiency (WHO Classification, paragraph 6.2) [8, 11, 36]. In individuals with mild or moderate deficiency, such as the A- G6PD variant (Class III), maximum rates of red-cell destruction are usually reached within 1–2 days and clinically detectable haemolysis and jaundice can typically arise within 24-72 hours of drug dosing [8, 9]. In these less-severe cases, haemolysis stops in about 7 days, because haematocrit values start to rise due to replacement of old G6PD-deficient erythrocytes with younger, more PQ-resistant red cells [11]. Furthermore, PQ is eliminated rapidly from the body (PQ's plasma half life is between 4 and 9 hours [35]) and so haemolysis is a self-limiting process provided no further drug is taken. In severe cases, such as those associated with G6PD Mediterranean variant (Class I and II), individuals may experience nausea, vomiting, abdominal or loin pain, and passage of dark urine due to haemoglobinuria [8, 9]. Anaemia develops rapidly and patients become jaundiced. This is associated with an abrupt fall in haemoglobin concentration of 3-4 g/dL, during which time the peripheral blood smear reveals red cell fragments, microspherocytes, and eccentrocytes or "bite" cells. Special stains document the production of Heinz bodies, which are collections of denatured globin chains often attached to the red cell membrane. Haemolysis is both extravascular and intravascular [8]. Rarely, haemoglobinuric renal failure occurs. In patients with this severe G6PD deficiency, continuation of PQ results in further haemolysis and life-threatening anaemia.
Haemolysis is more commonly observed after prolonged PQ treatment and 13 deaths from unsupervised prolonged PQ use have been reported: four in Sri Lanka, five in Turkey, two in Brazil, and one each in the UK and the USA [5]. No deaths are known to have followed single-dose administration [9], but haemolysis has also been observed in African populations following a single

dose of PQ [2, 26]. This haemolysis was self-limiting, largely restricted to G6PD deficient individuals and did not lead to clinical symptoms.

6.4. Assessing a safe and effective dose of PQ – data from Uganda

Since haemolysis is dose-dependent, side effects are expected to be less or absent when PQ is administered as a single low dose. If a lower dose of PQ would be efficacious in clearing gametocytes, this would make it a safe and powerful tool for community-wide malaria elimination strategies. Two studies in Thailand have suggested that lower doses of PQ than previously recommended by WHO (0.75 mg/kg) can be equally efficacious in clearing gametocytes. Bunnag compared the effect of 15 mg daily for 5 days, 30 mg single dose and 45 mg single dose in Thai adults and found no significant difference in gametocyte clearance between doses [37]. Pukrittayakamee compared 0.25 mg/kg and 0.5 mg/kg PQ in adults and found both to have shorter gametocyte clearance times (GCT) than non-PQ-containing regimens, with no significant difference in outcomes between the two doses of PQ [38]. A mass drug administration programme in Cambodia used a 9 mg stat dose of PQ (approximately 0.15 mg/kg) every ten days, with a significant reduction in microscopic gametocyte carriage from 13.1% to 0.8% after 3 years [39]. These studies have shortcomings in their safety and efficacy assessments. None of the studies included complete safety assessments and microscopy was used as gametocyte detection tool, which is notoriously insensitive for detecting low density gametocytes [40]. Furthermore, none of these studies determined the transmissibility of gametocytes persisting after PQ. These studies support the hypothesis that a lower, safer but efficacious dose of PQ may be found.

A more detailed study to provide evidence on efficacy of low dose PQ was recently completed in Jinja, Uganda by three of the researchers on this proposal. This trial was registered online at http://clinicaltrials.gov/ct2/show/NCT01365598 and completed in January 2013. In this study, 468 children (aged 1-10 years) with normal G6PD enzyme function were randomized to receive AL alone or with 0.75, 0.4 or 0.1 mg/kg PQ. None of the children suffered from episodes of acute haemolysis, defined in that study as a drop of 2g/dL or more in haemoglobin levels, clinical compromise, with signs of black/ tea-coloured urine or jaundice and haemoglobinuria, and no difference between AL and AL+PQ in changes in Hb concentration were seen [Eziefula *et al.,* Lancet Infect Dis *in press*]. The dose of 0.4 mg/kg was equally efficacious as the dose of 0.75 mg/kg in clearing submicroscopic gametocytes; the dose of 0.1 mg/kg showed inferior gametocytocidal activity. Because the study included individuals with normal G6PD function only, as defined by the fluorescent spot test, no conclusive evidence on safety in G6PD-unscreened populations was provided. Genotypically defined G6PD deficiency was determined after completion of the trial. The A376G/G202A mutation [34] was

detected in 17 male hemozygotes and 38 female heterozygotes who passed the fluorescent spot test (i.e. had normal enzyme function) and received PQ at 0.1-0.75mg/kg. In these genotypically G6PD-deficient individuals without severely affected enzyme function, there was no evidence of haemolysis. Acute malaria infection is associated with an expected transient fall in haemoglobin. In those with the A376G/G202A mutation, only those who received the highest dose of PQ (0.75mg/kg) had a greater fall in haemoglobin compared to placebo (AL alone).

In the current study, we propose to assess the risk of haemolysis associated with low doses of PQ (0.25 mg/kg and 0.40 mg/kg) in individuals with G6PD deficiency.

7. JUSTIFICATION OF THE STUDY

The previously recommended dosage for a single dose of PQ, 0.75 mg/kg, dates back to the 1940s and the current recommendation of 0.25 mg/kg PQ is based on limited safety and efficacy data. With regards to data from our recent study in Uganda [Eziefula 2013, under review], we believe there is sufficient evidence that 0.4 mg/kg and 0.75 mg/kg PQ are equally efficacious in clearing submicroscopic gametocytes. In the era of malaria elimination, an efficacious dose of PQ should be given to parasitaemic individuals with normal G6PD activity as well as G6PD deficient individuals, who have an increased risk of PQ-induced haemolysis. Therefore, using a dose-escalation design, the current study will assess safety of PQ when given together with an ACT (in this case AL) in asymptomatic parasitaemic G6PD deficient individuals. The current WHO-recommended dose (0.25 mg/kg) and the lowest efficacious dose (0.4 mg/kg), as defined by the recent trial, will be tested.

8. MAIN OBJECTIVE

To evaluate the tolerability and safety of increasing doses of PQ in combination with AL in G6PD deficient males with an asymptomatic *P. falciparum* malaria infection.

9. SPECIFIC OBJECTIVES AND HYPOTHESES TO BE TESTED

1. To evaluate the tolerability and safety of increasing doses of PQ when administered with AL in G6PD deficient, asymptomatic *P. falciparum* infected, males compared to control groups (G6PDd + AL only; G6PD normal + 0.25 mg/kg PQ; G6PD normal + 0.40 mg/kg PQ) as measured by:
    a. frequency and magnitude of adverse events
    b. change in Hb per individual and change in mean Hb in and between cohorts and treatment groups
    c. prevalence of severe anaemia (Hb <5g/dL)
    d. evidence of black urine (haemoglobinuria; dipstick positive)
    e. requirement for blood transfusion
2. To assess prevalence of different G6PD type mutations and safety of PQ in individuals with these different G6PD type mutations
3. To obtain basic pharmacokinetic parameters for PQ in a G6PD deficient population

10. DESIGN AND METHODOLOGY

10.1. General study design

The study is an open-label, dose-escalation Phase I trial. A minimum of 70 males between 18 and 45 years will be sequentially enrolled into five treatment groups. Two out of five treatment groups are intervention groups and 3 are control groups. The two intervention groups consist of 20 individuals each; doses of 0.25 and 0.4 mg/kg PQ + AL will be given, respectively. The 3 control groups consist of 30 individuals: 10 male participants with G6PD deficiency will receive AL only, 10 males with normal G6PD enzyme function will receive 0.25 mg/kg PQ + AL, and 10 males with normal G6PD enzyme function will receive 0.4 mg/kg PQ + AL.

There are two dosing cohorts. The first cohort will consist of:

i) the intervention group with G6PD deficiency receiving 0.25 mg/kg PQ + AL (n=20)
ii) the control group with G6PD deficiency receiving AL only (n=10)
iii) the control group with normal G6PD enzyme function receiving 0.25 mg/kg PQ + AL (n=10)
iv) the control group with normal G6PD enzyme function receiving 0.4 mg/kg PQ + AL (n=10)

The second cohort will consist of:

i) the intervention group with G6PD deficiency receiving 0.4 mg/kg PQ + AL (n=20)

The trial will be stopped according to criteria stated in section 10.5. Male participants will be recruited from Banfora health district, in the southwest of Burkina Faso, using village census lists and house-to-house visits of study teams. Screening lists are prepared based on census lists. Individuals aged 18-45 years who indicate a willingness to participate in the trial and sign an informed consent form for the screening procedure, will be tested for the presence of malaria parasites by microscopy and for haemoglobin levels by HemoCue; if parasites are present and Hb >11g/dL, G6PD deficiency will be tested by Beutler Fluorescent Spot test [41] (paragraph 10.11). Clinical screening will take place if malaria parasites are detected at any density in the absence of a history of fever and haemoglobin > 11g/dL. Study clinicians will assess eligibility for each individual to enter the study by i) taking medical history (including inclusion and exclusion criteria) and laboratory testing and ii) physical examination.

If individuals are eligible for enrolment, they will be enrolled into the lowest open cohort of the trial. Treatment allocation in cohort 1 will be randomised, using sealed envelopes. If G6PD deficiency test

indicates reduced enzyme function (<30% of normal function [41]), individuals will be enrolled in the intervention group (receiving 0.25 mg/kg PQ + AL) or control group (receiving AL only), or - if the first cohort has been completed – they will be enrolled without randomization in the intervention group receiving 0.4 mg/kg PQ + AL (details in table 2). If G6PD activity is normal, individuals will be enrolled in one of the other two control groups in cohort 1 (0.25 mg/kg PQ or 0.40 mg/kg PQ; details in table 2).

All enrolled individuals will receive a full three-day course of AL and - depending on treatment arm - PQ with the first dose of AL (day 0). All doses of AL and PQ will be directly observed. Participants will be reimbursed for travel to and from the clinic for all scheduled and non-scheduled visits during the time they are enrolled in the study. During follow up and beyond the last scheduled visit at day 28, all participants will be encouraged to attend the clinic for any medical concerns and the cost of travel to the clinic will be reimbursed.

Sampling will be as follows: all individuals will be sampled on T0, T12 (D0); T24, T36 (D1); T48, T60 (D2); T72, T84 (D3); D4, D5, D7, D10, D14 and D28. At these time-points, various measurements including full haematology, biochemistry and urine dipstick will be performed and samples will be tested for the presence of malaria parasites (asexual and sexual) by microscopy and molecular methods (for details: table 3 *Sampling schedule*). Five individuals in each intervention group with G6PD deficiency will be invited to participate in pharmacokinetic analysis, which will involve taking a total of 7 venous blood samples of less than 1 ml each on days 0, 1 and 2.

10.2. Outcome measures

Primary

1. haemoglobin concentration relative to baseline values (safety)

Secondary

2. haematology abnormalities during follow-up (safety): haptoglobin, MCV, RDW and leucocytes
3. biochemistry abnormalities during follow-up (safety): bilirubin, LDH, creatinine and potassium
4. frequency and severity of adverse events (safety)
5. gametocyte clearance time (efficacy)

10.3. Study site and population

The study will be carried out in the clinical trial field site of Banfora, Comoé province, 90 kilometres southwest of Bobo-Dioulasso. This site is being used for malaria vaccine trials and has an excellent infrastructure for clinical trials, including a well equipped clinical laboratory and staffed by skilled medical personnel.

Inclusion criteria:

1. Male gender
2. Age ≥18 years and ≤45 years
3. BMI ≥16
4. *P. falciparum* parasitaemia at any density
5. G6PD deficiency by Beutler Fluorescent Spot test for intervention groups and control group receiving AL only (N=50)
6. G6PD normal activity by Beutler Fluorescent Spot test for control groups (N=20)
7. Informed consent by participant

Exclusion criteria:

1. Enrolled in another clinical trial
2. Fever >37.5°C (tympanic) or history of fever in the last 24 hours
3. Evidence of severe illness / danger signs or active infection other than malaria
4. Known allergy to study medications
5. Hb <11 g/dL
6. Antimalarials taken within the last 2 weeks
7. PQ taken within the last 4 weeks and blood transfusion within the last 90 days
8. Non-falciparum malaria co-infection
9. Current use of tuberculosis or anti-retroviral medication, sulphonamides, dapsone, nitrofurantoin, , nalidixic acid, ciprofloxacin, , methylene blue, toluidine blue phenazopyridine and co-trimoxazole.
10. History of severe chronic illness

10.4. Participant screening and recruitment

Study subjects will be recruited from villages surrounding the Banfora clinical trial centre. Census lists will be prepared to screen individuals in the proposed age-range who will have a blood slide and hemoglobin levels measured by HemoCue. Screening blood smears will be read and counted by the

outpatient laboratory technicians; parasitemic individuals with normal Hb levels (Hb>11g/dL) will be tested for G6PD enzyme function by Beutler test [41].
Upon referral to the study clinic, a standardized screening interview and physical examination will be conducted by study clinicians. This interview will go through the initial screening selection criteria that are given above. All individuals with fever or other symptoms suggestive of infection are excluded from study enrolment since infection (symptomatic malaria, pneumonia, hepatitis, typhoid fever, among others) itself might precipitate G6PD-related haemolysis; these individuals will be referred back to the standard outpatient clinic for diagnosis and treatment of their acute clinical condition.

10.5. Enrolment

Individuals who agree to participate for screening and meet all inclusion criteria will be invited for enrolment. During enrolment procedures, they will again be informed about the objectives and practical consequences of participation and asked to sign an informed consent form. The possibility of withdrawal from the study, at any time and without any declaration of the reason will again be pointed out. Participants will then be invited to sign the written consent form adjoining the written study information and approved by the IRBs to participate in this clinical trial; participants will also be invited to sign consent for the future use of biological specimens obtained during the course of the study. If the participant is unable to write, their fingerprint will be used in substitute for a signature, and a signature from an impartial witness to the informed consent discussion will be obtained. Two copies of the consent form must be signed. The participant / impartial witness will sign / fingerprint one copy for the study staff and one copy to keep for themselves.
Following the informed consent discussion, participants will be given their copy of the form to keep which includes the study information, the signed consent form and contact names and telephone numbers to use if they have further questions regarding the study or follow up procedures.

10.6. Dose escalation protocol

After enrolment, participants will be assigned to the lowest-dose open cohort, with enrolment in the second cohort initiated after tolerability and short-term safety is demonstrated at the preceding lower dose (this enrolment to the second cohort accounts for G6PD deficient participants only). Within each cohort, the first 2 participants of the intervention group are treated and monitored for 6 days for immediate side-effects and haematological abnormalities before the rest of the participants of that particular intervention group are enrolled and treated. Once safety of these first 2

participants is confirmed, the next 4 subjects are enrolled and treatment for the next 4 subjects is initiated on day 2 of the last treated participant of the preceding 4 subjects. The last two groups for each intervention group comprise 5 individuals, making a total of 20. After inclusion of the intervention group of the first cohort (n=20) is completed (follow-up day 14 of last participant in that group), a 10-day safety observation period is installed before enrolment of the intervention group of the second cohort is initiated.

Table 2: Study cohorts and dose escalation schedule

Cohort 1:

	**INTERVENTION 0.25 mg/kg PQ + AL G6PD def.**	**CONTROL AL G6PD def.**	**CONTROL 0.25 mg/kg PQ+AL G6PD sufficient**	**CONTROL 0.4 mg/kg PQ+AL G6PD sufficient**
	Randomization = 2:1		Randomization = 1:1	
**Total participants**	20	10	10	10

Cohort 2:

	**INTERVENTION 0.4 mg/kg PQ + AL G6PD def.**
**Total participants**	20

The following scenarios will prompt urgent DSMB review (< 24hours) to consider whether it is appropriate to stop the trial:

1. There is any drop in Hb below 5 g/dL
2. There is any need for blood transfusion
3. 3 or more participants have a drop in Hb >4 g/dL
4. There is any requirement for haemodialysis
5. Any severe adverse event defined as any untoward medical occurence or effect that at any dose: results in death, is life threatening, requires hospitalization (attributable to the study drug), results in persistent or significant disability or incapacity.

If an enrolled participant discontinues the study for reasons unrelated to treatment before completing the final visit on day 28, an additional participant will be enrolled in the same group. This

process will be repeated until either both cohorts have their follow-up completed or enrolment to a subsequent cohort is suspended because stopping criteria are met.

10.7. Treatment and administration procedures

All participants will be treated with artemether-lumefantrine (AL; Coartem®; Novartis Pharma) administered as 4 tablets (20 mg of artemether and 120 mg of lumefantrine) in a 6-dose regimen (at enrolment and 8, 24, 36, 48 and 60 h [±90 min] after initiation of treatment). For all groups except the G6PD deficient group receiving only AL (n=10), the first dose is given together with 0.25 or 0.4 mg/kg PQ.

All treatment is administered under supervision with fatty food to ensure adequate absorption and minimize the risk of gastro-intestinal side effects. All doses are given at the clinic under supervision of the study nurse. For each dose the study nurse will document whether the treatment has been given or not, the number of tablets given and whether or not the dose was vomited or repeated.

For participants who develop anaemia (Hb <11 g/dL) during the study, we will follow international and Burkina Faso national treatment guidelines.

10.8. Follow-up and samples taken

After initiation of treatment, participants are asked to return to the clinic on the evening of day 0, twice daily on days 1, 2, and 3, and once daily on days 4, 5, 7, 10, 14 and 28. On days 0, 1 and 2 study drugs are administered twice daily. On all visits, participants will be examined clinically (except for day 28) and a structured questionnaire is used to determine the occurrence of side effects. During screening, twice daily on days 0, 1, 2, 3, and once daily on days 4, 5, 7, 10, 14 and 28 after initiation of treatment, participants will have a Hb sample taken by finger prick. Hb concentration is determined by HemoCue photometer [Angelholm, Sweden].

A blood slide will be taken once daily on all days - except day 4 and 5 - to determine whether asexual parasites and gametocytes have been cleared. A longer follow-up is not needed since currently, there are no indications that PQ influences the clinical efficacy of AL and the study is purely designed to determine safety in the first four weeks after initiation of treatment in G6PD deficient individuals.

An RNA sample for gametocyte detection by quantitative nucleic acid sequence based amplification (QT-NASBA) will be collected on days 0, 2, 3, 7, 10 and 14 [2]. Biochemistry (ASAT, ALAT, total Bilurubin, Creatinin) and full blood count (including reticulocytes) are done for all individuals twice daily on days 0 (enrolment) and 1, and once daily on days 3, 7, 14 and 28. Filter paper blood samples will be collected at screening and on day 14 for quantification of G6PD activity. Dried blood spots will

also be used to determine G6PD genotype and mutations in the CYP450 genes retrospectively, which may influence PQ metabolism and therefore haemolysis.

Urine dipstick will be done twice daily on days 0, 1, 2 and 3 and once daily on days 4, 5, 7 and 28.

Table 3. Sampling schedule

	Scree-ning	Outpatient visits														Unscheduled
**Day of follow-up**		**0 M.**	**0 E.**	**1 M.**	**1 E.**	**2 M.**	**2 E.**	**3 M.**	**3 E.**	**4**	**5**	**7**	**10**	**14**	**28**	
**CLINICAL:**																
History		X	X	X	X	X	X	X	X	X	X	X	X	X	X	X
Tympanic temperature		X	X	X	X	X	X	X	X	X	X	X	X	X	X	X
Physical examination		X	X	X	X	X	X	X	X	X	X	X	X	X		X
Assessment for adverse events		X	X	X	X	X	X	X	X	X	X	X	X	X	X	X
Complete case record form		X	X	X	X	X	X	X	X	X	X	X	X	X	X	X
**TREATMENT:**																
AL		X (1)	X (2)	X (3)	X (4)	X (5)	X (6)									
PQ		X (1)														
**LAB TESTING:**																
Blood smear	X	X		X		X		X				X	X	X	X	X‡
RNA sample (QT-NASBA)		X				X		X				X	X	X		
Haemoglobin (Hemocue®)	X	X	X	X	X	X	X	X	X	X	X	X	X	X	X	X
Biochemistry / full blood c.		X	X	X	X			X				X		X	X	X‡
Urine dipstick		X	X	X	X	X	X	X	X	X	X	X			X	X‡
G6PD screen	X													X		

‡on indication

10.9. Parasite detection

Samples for parasite detection (blood smear and RNA samples) will be collected from finger prick (~200 µL). Blood smears are stained with 10% Giemsa for 10 minutes and then screened for asexual parasites and gametocytes at screening and on days 0, 1, 2, 3, 7, 10, 14 and 28 after initiation of treatment. Slides are double-read and read separately for asexual parasites and gametocytes in 100 microscopic fields. Parasite detection by Pfs25 QT-NASBA will be done using a NucliSens EasyQ analyser (bio-Mérieux) as described elsewhere for Pfs25 mRNA [43, 44]. Nucleic acids are extracted by automated extraction [Roche MagNapure] using commercial kits from 50µL blood samples. The

Pfs25 QT-NASBA technique is gametocyte specific and has an approximate detection limit of 0.1 gametocytes/µL in the blood volume taken. NucliSens Basic kits are used for amplification in accordance with the manufacturer's instructions. A standard dilution series of mature, *in vitro* cultured NF54 gametocytes [44] is included in each run. Gametocyte detection by *Pfs25* QT-NASBA is done for all samples on days 0, 2, 3, 7, 10 and 14 after initiation of treatment. Day 28 is excluded from this analysis because gametocyte prevalence on this day may be due to re-infections [45].

10.10. Haematology and biochemistry

On all time points Hb will be assessed using a HemoCue® photometer (Ängelholm, Sweden). This is done in order to avoid discrepancies between HemoCue® and full blood count Hb results. The HemoCue® produces a point-of-care result. A venipuncture sample will be taken twice daily (1.5 mL per sample for haematology; 2.5-3 mL per sample for biochemistry) on days 0 (enrolment) and 1, and once daily on days 3, 7, 14 and 28 for a full blood count (e.g. haemoglobin, white blood cell count, reticulocytes, platelets and haematocrit) and biochemistry (renal and liver function tests). On any day or time when the clinician feels this is required for the investigation of adverse events, additional samples will be taken. These samples will be used for a full blood count and biochemistry including, for example, renal and liver function testing and urinalysis for haemolysis.

10.11. G6PD deficiency testing

An EDTA tube or heparinized haematocrit capillary tube will be used to acquire blood for glucose-6-phosphate-dehydrogenase semi-quantitative fluorescent spot test for initial screening in the clinic site. This will require approximately 0.5 ml of blood. A reagent solution containing Glucose-6-P + NADP+ is mixed with whole blood. Samples obtained from normal or slightly reduced G6PD activity will show strong fluoresce. Failure to fluoresce after 10-minutes of incubation suggests a total or marked deficiency of G6PD. This test may fluoresce falsely if the study participant has had a blood transfusion within the last 90 days hence, these persons will be excluded from the study. In addition, dried blood spots obtained on filter paper on screening and day 14 will be stored for quantitative G6PD testing. This will be performed using the G-6-PD OSMMR 2000 kit [R&D Diagnostics]. The dried blood spots will also be utilized for G6PD &CYP450 genotyping.

10.12 Pharmacological analysis

Pharmacokinetic evaluations will be obtained on approximately one quarter of the participants in intervention groups with G6PD deficiency, a maximum of 10 G6PD deficient participants.

Information on pharmacokinetic analysis is included in the study information sheet and procedures will be explained to participants on day 0; those who are selected and agree to participate will be asked to come for sampling on days 0 to 2. The sampling on day 0 will happen whilst they are at the clinic for their first day of AL and the study dose of PQ.

The pharmacokinetic sampling will involve taking a total of 7 venous blood samples of less than 1mls. The total amount sampled, being approximately 7 mL in 3 days. The first sample is just prior to the PQ dose (a baseline sample) and the subsequent six doses are at intervals up to 72 hours after the dose of PQ. The blood samples will be taken at fixed times between 8am to 5pm. The first 5 samples are taken on day 0 and they will be taken through a venflon, placed when the baseline pharmacokinetic sample is taken. If a venflon is not placed successfully, a butterfly needle may be used. The last two samples (one on day 1 and one on day 2) will be taken by individual blood draws (venipuncture). The participant will be asked to stay in the clinic between sampling times on day 0.

In order to minimize the total number of blood draws per participant, the sampling timeframe has been randomized so that over the total population of participants, a population pharmacokinetic model can be constructed for analysis. Six randomized sample times will be allocated to sequential consenting participants in opaque envelopes. Each sample time is within a window so that there are 5 samples on day 0 and one each on days 1 and 2. Pharmacokinetic samples will be analysed in Professor Niklas Lindegardh's laboratory in Mahidol University, Bangkok, Thailand, where the randomized sampling framework was generated.

Selection for pharmacokinetic analysis

On day 0, pharmacokinetic procedures will be explained and consent obtained (in Clinical study informed consent form); every 4th G6PD deficient adult who was enrolled in an intervention group will be invited to participate in pharmacokinetic sampling. If this study participant declines inclusion in the pharmacokinetic study, the next consecutive participant will be invited. If a participant is included and vomits within 30 minutes of primaquine dose, the next participant will be invited. The Clinical Study consent interview will be conducted by study clinicians. . All participants undergoing pharmacokinetic sampling must satisfy the following criteria in addition to the study's inclusion criteria:

Pharmacokinetic analysis inclusion criteria:

1. Siting of secure blood sampling access feasible (venflon/ butterfly needle) on day 0
2. Willing and able to attend study clinic by 7.30am on days 0-2
3. Willing to stay on study clinic premises between 8am to 5pm on day 0

Study subjects included in the pharmacokinetic analysis will be seen by a study clinician who will select the next available pharmacokinetic opaque envelope. This will contain a sheet with the sampling times for the participant. The clinician will label this sheet with the participant's study number. The clinician will be responsible for adhering to the sampling times.

The clinician will fix venous access (using a venflon or butterfly) and take the baseline sample. All blood will be taken into heparinised tubes. The subsequent 4 samples will be taken at the allocated sampling times (Table 4). Then the fixed venous access will be removed. The participant will be informed as to what time they need to present by on days 1 and 2 for the last two samples. On these days, the sample will be taken by phlebotomy with no fixed access.

Table 4 Sampling framework for participants recruited to pharmacokinetic studies

**Day of follow up**	0	1	2
PQ pharmacokinetic sampling windows:*<br><br>(baseline plus x 6 windows)<br><br>Serum samples	0<br><br>0-2<br><br>2-3<br><br>3-6<br><br>6-9	24-33	48-72

*each participant has one sample within each sampling window

11. SAMPLE SIZE CONSIDERATIONS

For each of the 2 intervention groups (PQ in G6PD deficients) 20 individuals will be enrolled. This number of individuals per group will give us a 88% probability of detecting at least one severe event and ~61% probability of detecting at least 2 severe adverse events if we assume that the probability of a serious adverse event is 10% for each individual. The control groups are included to support the interpretation of haemoglobin concentrations following treatment (i.e. a reference group where a reduction in Hb is not due to primaquine in combination with low G6PD enzyme activity); the size of these control groups was based on expert opinion (WRAIR, Tafenoquine group) and not based on sample size calculations.

12. DATA ANALYSIS PLAN

The primary safety outcome, risk of haemolysis, defined as the proportion of individuals in each group that has a drop of >2.5 g/dL in haemoglobin levels, will be calculated. Mean (± SD) maximal fall (±) in Hb (haemoglobin, g/dl) from enrolment to day 28 of follow-up, follow-up day of Hb nadir, maximal percentage fall in Hb level compared to enrolment value, and evidence of black urine (urine dipstick positive for Hb) will be determined. The incidence of serious adverse events by sign, symptom, laboratory parameter and relationship to taking study drug will also be determined.

13. ETHICAL CONSIDERATIONS

This study includes G6PD deficient individuals who are at an increased risk of haemolytic side-effects of PQ as well as individuals with normal G6PD function. Within the G6PD deficient population, safety of the AL-PQ combination will be studied thoroughly. This information is essential since all parasitologically-confirmed *P. falciparum* malaria patients in elimination programmes will now receive a single dose of PQ on the first day of treatment with ACT according to new WHO policy guidelines. This includes G6PD deficient individuals who account for up to 30% of the population in some malaria-endemic areas [8, 11].

The individual participant will benefit from treatment for their malaria infection. The treatment given, AL-PQ, is recommended by the WHO for elimination settings although the PQ component provides no immediate benefit to the individual. At a population level, treatment with PQ can reduce malaria transmission [9] and lead to a lower risk of infection experienced by individual community members [46]. This study is an essential requirement before transmission-reducing interventions with community benefit can be implemented.

The current study will restrict enrolment to asymptomatically infected males with normal Hb values. Careful monitoring during 28 days will minimize the risk of participation for individuals. Clinical staff will receive training on standardised protocols for the detection and management of haemolysis. Blood sampling will be restricted to the absolute minimum to answer the study questions, but may be increased in some individuals to monitor safety parameters. The total blood volume that is taken per participant during their 28-day period of participation will be ~25mL. Participants will be compensated for their participation.

13.1. Confidentiality

All participant records are to be used only for the purpose of this research project. Names will not appear on labels on laboratory specimens or in any report resulting from the study.
Identifying information will be kept in a metal cabinet that is locked and only accessible to the study clinician or his clinical representative in case he is absent. Paper forms are only accessible to senior research staff (i.e. clinician, nurse and scientific personnel but not field workers or laboratory assistants); names and addresses will be removed from digital files which will be password protected. All materials collected in this study will be labelled with a study identification number that cannot be directly linked to identifying information.

13.2. Participant reimbursement

Participants will receive a small parcel with items such as sugar, rice and flour on a daily basis to compensate them for the time lost during the study. This is common practice for all clinical trials at CNRFP.

13.3. Good clinical practice

The study will be conducted in accordance with the latest South Africa revision of the Declaration of Helsinki and local regulatory requirements, and study staff will receive a local workshop in Good Clinical Practice before the study starts. AL is the first line antimalarial drug in Burkina Faso; PQ is a registered drug that has been extensively used over the last 50 years and is recommended by the WHO. A data safety and monitoring board will be installed to ensure the trial is completed according to GCP standards.

14. SAFETY MANAGEMENT

14.1. Safety management

14.1.1. Withdrawal of individual subjects

Subjects can leave the study at any time for any reason if they wish to do so without any consequences. The principal investigators and trial clinicians can decide to withdraw a subject from the study for urgent medical reasons.
Subjects can be withdrawn from the study procedures for the following reasons:

- Any serious adverse event (SAE)
- Any AE that, according to clinical judgment of the trial clinicians, is considered as a definite contra-indication to proceeding with the study procedures
- Withdrawal of informed consent by subject
- Failure to comply with the prescribed study protocol or complete loss to follow-up
- Repeated vomiting within 30 minutes after administration of study drug

14.1.2. Premature termination of the study

The study may be discontinued for the following reasons:

- On advice of the local safety monitor (LSM)
- On advice of the data safety monitoring board (DSMB)
- On advice of the principal investigators and trial clinicians
- On advice of the ethics committee

The LSM, DSMB, principal investigators, trial clinicians or ethics committee may decide to put the study on hold based on adverse events, pending discussion with the LSM / DSMB / principal investigators / trial clinicians / ethics committee. Following discussion, it may be decided to terminate the study.

14.2. Management of haemolysis

In previous studies PQ has never been exclusively administered to G6PD deficient individuals. However, we know that in nearly 1500 patients studied prospectively in trials of single dose PQ given as a gametocytocide in Africa, Asia and South-America, no severe haemolysis requiring blood transfusion was reported [3, 9, 24-29]. Furthermore, in Tanzania [2], none of the participants (who all had a normal G6PD enzyme function) experienced symptoms of anaemia and no child required a

blood transfusion. In the second study in Tanzania [26], one G6PD deficient child who received PQ 0.75mg/kg had severe anaemia, but did not require a blood transfusion and recovered with haematinic drug treatment. In Sudan [25], there were no severe or serious or adverse events and severe anaemia was not reported. In Uganda [Eziefula, under review], a subanalysis by G6PD genotype revealed that individuals with A- genotype had a reduced fall in haemoglobin with 0.4mg/kg and 0.1mg/kg, compared to the fall with 0.75 mg/kg and there were no episodes of severe haemolysis or requirement for blood transfusion.

Asymptomatic malaria infection is expected to precipitate a small degree of haemolysis, due to erythrocive parasitisation. However, given that we test lower doses of PQ than those previously associated with haemolysis and we administer PQ at a single time-point, we do not expect significant haemolysis to occur at any point during the study. Furthermore, the dose-escalation schedule and halting criteria allow us to conduct this trial safely and will minimise the risk of haemolysis.

For the purposes of systematic and responsible safety monitoring, the following detailed protocols have been developed for the management of participants in whom haemolysis is suspected.

14.2.1. Measures of haemolysis

Haemolysis will be diagnosed according to criteria in a study SOP, detailing the size of haemoglobin fall (measured by Hemocue®) after PQ treatment and the absolute haemoglobin value. In addition, any participant presenting with or complaining of tea-coloured or black urine will be assessed for haemolysis. If haemolysis is suspected, a venipuncture sample will be taken for a full blood count, biochemistry and Hb by Hemocue, a blood film will be prepared and analysed for abnormal red blood cells, urine dipsticks and urine microscopy will be taken and a clinical examination will be performed.

Anaemia

Participants with haemoglobin below 11 g/dL at screening will not be included and treated with haematinic drugs and de-worming according national guidelines.

Haemolysis

Participants with evidence of mild haemolysis will be observed and monitored and haematinic drugs will be considered according to national guidelines. Haemoglobin testing will be repeated according to the participant's clinical progress. Any participant reporting black urine will be assessed by a study clinician. Black urine is defined as any dark-coloured urine with brown or black pigments (not orange). A full clinical exam and history will be obtained, a urine sample sent for analysis (evidence

of haematuria, proteinuria) and a venous blood sample drawn for assessment of full blood count, biochemistry and renal function (including bicarbonate). A blood film will be assessed for abnormal red blood cells (schistocytes). Requirement for blood transfusion will be assessed by trial clinicians, considering the size and rate of Hb drop and signs of clinical compromise, according to national guidelines.

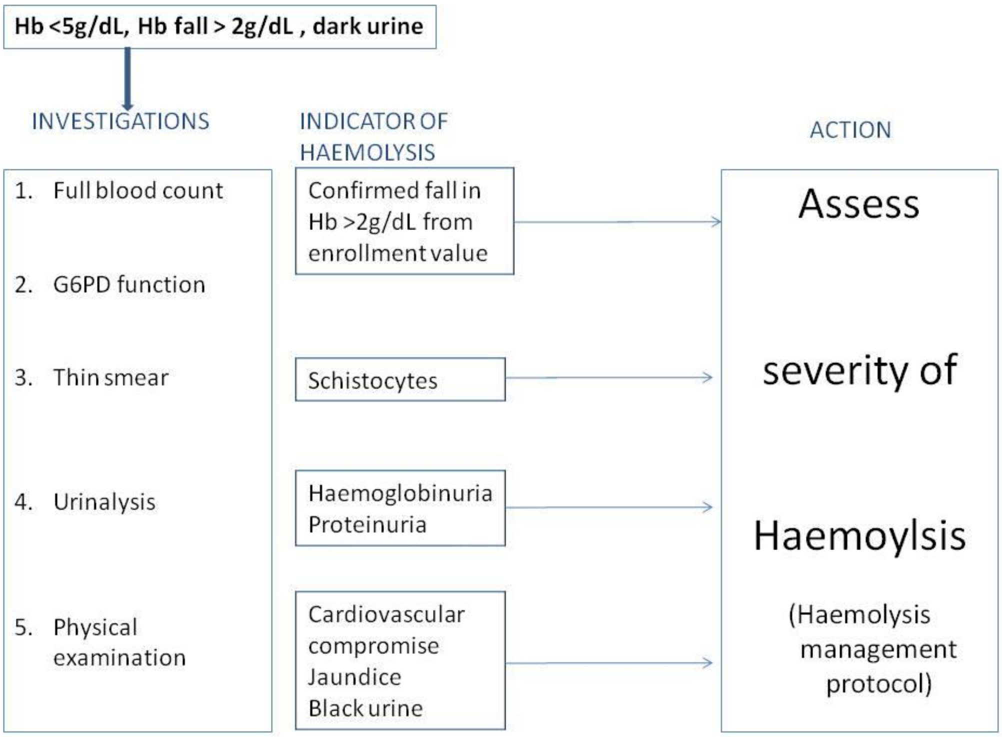

Figure 1. Investigation of haemolysis

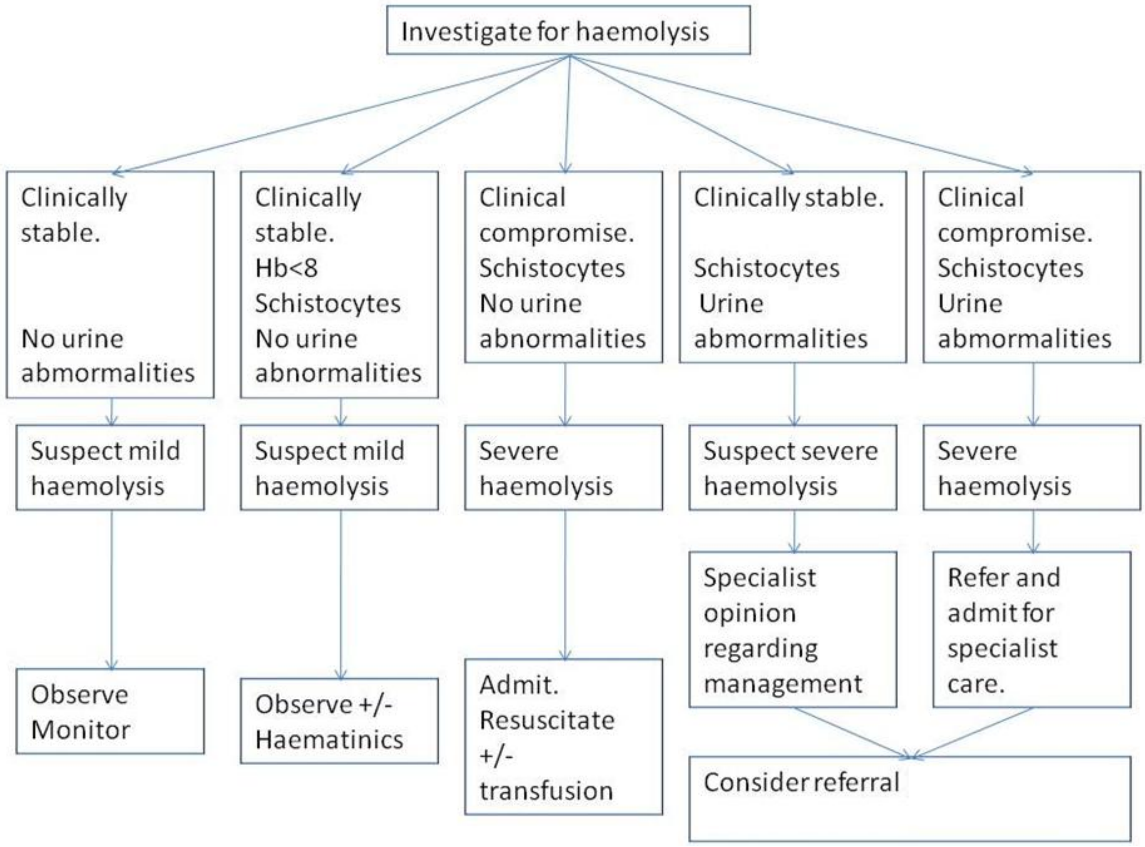

Figure2. management of haemolysis

14.3. Safety reporting

14.3.1. Adverse events

Adverse events are defined as any undesirable experience occurring to a subject during the study, whether or not considered related to trial. An AE can therefore be any unfavourable and unintended sign (including an abnormal laboratory finding), symptom, or disease (new or exacerbated) temporally associated with a study intervention. AEs may include events that occur as a result of protocol-mandated procedures (i.e. invasive procedures). All AEs reported spontaneously by the subject or observed by the trial clinicians or their staff will be recorded.

Abnormal laboratory findings or other abnormal assessments that are judged by the trial clinicians to be clinically significant will be recorded as AEs or serious adverse events (SAEs) if they meet the definition. The principal investigators and trial clinicians will exercise their medical and scientific judgement in deciding whether an abnormal laboratory finding or other abnormal assessment is clinically significant.

14.3.2. Serious adverse events

A serious adverse event is any untoward medical occurrence or effect that at any dose:

- Results in death
- Is life threatening (at the time of the event)
- Results in persistent or significant disability or incapacity
- Is a new event of the trial likely to affect the safety of the subjects, such as an unexpected outcome of an adverse reaction or a major safety finding from a newly completed animal study etc.

All SAEs will be reported immediately to the principal investigators. The latter will subsequently report to the Sponsor (London School of Hygiene and Tropical Medicine) within 24hrs of awareness and to the chairpersons of the DSMB and Ethics Committee. (for more information: sections 13.3.3 and 13.4)

14.3.3. Adverse Event Data Collection

Safety assessments will be performed, and recorded by the trial clinicians. All AEs/reactions, observed by the trial clinicians or by the subject, will be accurately documented in the case report form. For each event/reaction the following details will be recorded:

1. Description of the event(s)/reaction(s)
2. Date and time of occurrence
3. Duration
4. Intensity
5. Relationship with the intervention
6. Action taken, including treatment
7. Outcome

In addition, symptoms will be ranked as (1) mild, (2) moderate, or (3) severe, depending on their intensity. All AEs except fever will be judged for their intensity according to the following scale:

- Mild (grade 1): awareness of symptoms that are easily tolerated and do not interfere with usual daily activity
- Moderate (grade 2): discomfort that interferes with or limits usual daily activity
- Severe (grade 3): disabling, with subsequent inability to perform usual daily activity, resulting in absence or required bed rest

- Life-threatening (grade 4): extreme limitation in activity, significant assistance required; significant medical intervention/therapy required, hospitalization required

For details regarding the grading of symptoms we refer to the NIH Division of Microbiology and Infectious Diseases Adult Toxicity Table:
http://www.niaid.nih.gov/LabsAndResources/resources/DMIDClinRsrch/pages/toxtables.aspx

For fever, the following scale will be used:

- Mild (grade 1): 37.5 - 38.0°C
- Moderate (grade 2): > 38.0 - 39.0°C
- Severe (grade 3): > 39.0°C

If an AE changes in frequency or intensity during the specified reporting period, the previous description of the AE will be corrected.
When an AE/SAE occurs, it is the responsibility of the principal investigators and trial clinicians to review all documentation (e.g. hospital progress notes, laboratory, and diagnostics reports) related to the event. The principal investigators and trial clinicians will then record all relevant information regarding an AE/SAE on the CRF or SAE Report Form, respectively. Furthermore, the trial clinicians will attempt to establish a diagnosis of the event based on signs, symptoms, and/or other clinical information. In such cases, the diagnosis should be documented as the AE/SAE and not the individual signs/symptoms.

14.3.4. Assessment of causality

The principal investigators and trial clinicians are obligated to assess the relationship between study procedures and the occurrence of each AE/SAE. The clinicians will use clinical judgment to determine the relationship. Alternative causes, such as natural history of the underlying diseases, concomitant therapy, other risk factors and the temporal relationship of the event to the study procedures will be considered and investigated. The relationship of the AE with the study procedures will be categorized as:

Definite	An AE that follows a clear cut temporal association with a positive re-challenge test of laboratory confirmation
Probable	An AE that follows a reasonable temporal sequence from the study procedures and cannot be reasonably explained by the known characteristics of the subject's clinical

	state.
Possible	An AE for which insufficient information exists to indicate a high improbability that the event is related to the study procedures.
Not related	An event for which sufficient information exists to indicate that the etiology is unrelated either because of the temporal sequence of events or because of the subject's clinical state or other therapies.

14.4. Safety follow-up

13.4.1 Follow-up of subjects that voluntarily withdraw participation

If a subject fails to appear for follow-up, effort will be undertaken to locate or recall him/her or at least to determine his/her health status. These efforts will be documented in the subject's CRF and source documents.

In the event that a subject discontinues the study for any reason, the trial clinicians will conclude appropriate safety assessments for the subject and, in the case of an (S)AE, which has not yet resolved, they will schedule follow-up visits until the AE resolves or stabilizes.

14.4.2. Follow-up of adverse events

All AEs will be followed until they have abated, or until a stable situation has been reached. Depending on the event, follow-up may require additional tests or medical procedures as indicated, and/or referral to other specialists as the trial clinicians see fit.

The trial clinicians will follow-up subjects:

- with SAEs or those withdrawn from the study as a result of an AE, until the event has resolved, subsided, stabilized, disappeared, the event is otherwise explained, or the subject is lost to follow-up.
- with other non-serious AEs, until the subject has completed the study or is lost to follow-up.

Clinically significant laboratory abnormalities will be followed up until they have normalized, or until an alternative explanation, that is not related to the study has been provided. Additional information (including but not limited to laboratory results) relative to the subsequent course of such an abnormality will be made available to the LSM.

AEs reported during the trial will be assessed and classified as:

1. Recovered/resolved
2. Not recovered/not resolved
3. Recovering/resolving
4. Recovered with sequelae/resolved with sequelae
5. Fatal

14.4.3. Follow-up of serious adverse events

In the case of a suspected SAE, the nurses or trial clinicians will immediately report this occurrence to the principal investigators (PIs). The latter will subsequently report to the Sponsor (London School of Hygiene and Tropical Medicine) within 24hrs of awareness and to the chairpersons of the DSMB and Ethics Committee. In their report, the PIs will provide as much details as possible about the suspected SAE. At the same time the PIs, in consultation with the trial clinicians will stop any administration of study drugs, if the SAE is thought to be as a result of the study drug. The trial clinicians and consultant clinicians will immediately institute appropriate medical treatment to the subject who develops an SAE until the event has resolved, subsided, stabilized, disappeared, or the event is otherwise explained.

All findings of investigations and treatments administered will be recorded on the subject's CRF and also on the SAE Report Form. The trial clinicians may also refer the subject to other specialists as they see fit. The PIs will forward the completed SAE form to the LSM to assess whether the SAE could have been a result of the study procedures, as per standard criteria. Findings of this assessment will be forwarded to the sponsor (LSHTM) and to the chairpersons of the DSMB and Ethics Committee.

14.5. LSM and Data Safety Monitoring Board (DSMB)

For this study, a LSM is appointed, who is based in Banfora and will be involved in the review of SAEs and subject safety. He is an experienced clinician and independent of the investigators team. His main responsibility will be the assessment of the events and recommendation regarding halting further study procedures.

Furthermore, an independent DSMB has been appointed, which consists of three skilled professionals constituted according to sponsor procedures that respect international standards. These experts will monitor the progress of the study with particular interest in the safety of trial subjects.

The DSMB will be responsible for:

- Reviewing safety and follow-up
- Reviewing reports on SAEs when they occur
- Making recommendations to the sponsor on the safety balance between comparison groups
- Making recommendations regarding continuing, amending or termination of the study for safety reasons

The LSM and DSMB will receive progress reports by email after the first 10 individuals completed day 14, then after the second 10 individuals completed day 14, etc. This will result in a total 7 safety reports at the end of the trial. These reports will be prepared by the PIs and trial clinicians and include a list of all reported AEs and any safety laboratory values (e.g. Hb, ALAT/ASAT) outside the normal range.

All SAEs will be reported by the PIs to the Sponsor (LSHTM), the LSM, the chairpersons of the DSMB and Ethics Committee within 24 hours of awareness. The DSMB is empowered to suspend the enrolment to the trial pending review of potential safety issues.

14.6. Data monitoring

A local independent data monitor from CNRFP will undertake data monitoring at site (e.g. source data verification). In addition, there will be central monitoring by a monitor that will be appointed in consultation with the sponsor. This central monitoring will undertake statistical monitoring of inliers and outliers, data management review etc.

15. TIME FRAME AND DURATION OF THE PROJECT

Data collection will take place in 2014. The enrolment is envisaged to take 8 weeks; the clinical follow-up 4 weeks per individual. Community sensitization is expected to start in February 2014, the data collection will take place between March 2014 - May 2014. The project is followed by a community meeting to present some of the preliminary findings. Data analysis will take place in the second half of 2014.

16. REFERENCES

1. World Health Organization: **Malaria elimination: a field manual for low and moderate endemic countries.** 2007.
2. Shekalaghe S, Drakeley C, Gosling R, Ndaro A, van Meegeren M, Enevold A, Alifrangis M, Mosha F, Sauerwein R, Bousema T: **Primaquine clears submicroscopic Plasmodium falciparum gametocytes that persist after treatment with sulphadoxine-pyrimethamine and artesunate.** *PLoS One* 2007, **2:**e1023.
3. Smithuis F, Kyaw MK, Phe O, Win T, Aung PP, Oo AP, Naing AL, Nyo MY, Myint NZ, Imwong M, et al: **Effectiveness of five artemisinin combination regimens with or without primaquine in uncomplicated falciparum malaria: an open-label randomised trial.** *Lancet Infect Dis* 2010, **10:**673-681.
4. Bousema T, Okell L, Shekalaghe S, Griffin JT, Omar S, Sawa P, Sutherland C, Sauerwein R, Ghani AC, Drakeley C: **Revisiting the circulation time of Plasmodium falciparum gametocytes: molecular detection methods to estimate the duration of gametocyte carriage and the effect of gametocytocidal drugs.** *Malar J* 2010, **9:**136.
5. Abeyaratne KP, Halpe NL: **Sensitivity to primaquine in Ceylonese children due to deficiency of erythrocytic glucose-6-phosphate dehydrogenase.** *Ceylon Med J* 1968, **13:**134-138.
6. Reeve PA, Toaliu H, Kaneko A, Hall JJ, Ganczakowski M: **Acute intravascular haemolysis in Vanuatu following a single dose of primaquine in individuals with glucose-6-phosphate dehydrogenase deficiency.** *J Trop Med Hyg* 1992, **95:**349-351.
7. Khoo KK: **The treatment of malaria in glucose-6-phosphate dehydrogenase deficient patients in Sabah.** *Ann Trop Med Parasitol* 1981, **75:**591-595.
8. Cappellini MD, Fiorelli G: **Glucose-6-phosphate dehydrogenase deficiency.** *Lancet* 2008, **371:**64-74.
9. White NJ: **Primaquine to prevent transmission of falciparum malaria.** *Lancet Infect Dis* 2013, **13:**175-181.
10. Eziefula AC, Gosling R, Hwang J, Hsiang MS, Bousema T, von Seidlein L, Drakeley C, Primaquine in Africa Discussion G: **Rationale for short course primaquine in Africa to interrupt malaria transmission.** *Malar J* 2012, **11:**360.
11. Beutler E: **G6PD deficiency.** *Blood* 1994, **84:**3613-3636.
12. **Chemotherapy of malaria and resistance to antimalarials. Report of a WHO scientific group.** *World Health Organ Tech Rep Ser* 1973, **529:**1-121.
13. Bruce-Chwatt LJ: **Malaria research and eradication in the USSR. A review of Soviet achievements in the field of malariology.** *Bull World Health Organ* 1959, **21:**737-772.
14. Thimasarn K: **Current measures of containment of multi-drug resistant falciparum malaria in Thailand.** *Southeast Asian J Trop Med Public Health* 1992, **23 Suppl 4:**139-142.
15. Clyde DF: **Clinical problems associated with the use of primaquine as a tissue schizontocidal and gametocytocidal drug.** *Bull World Health Organ* 1981, **59:**391-395.
16. Clyde DF: **Mass administration of an antimalarial drug combining 4-aminoquinoline and 8-aminoquinoline in Tanganyika.** *Bull World Health Organ* 1962, **27:**203-212.
17. Yip K: **Antimalarial work in China: a historical perspective.** *Parassitologia* 1998, **40:**29-38.
18. Kondrashin AV, Baranova AM, Sergiev VP: **[Widespread use of primaquine for control of Plasmodium vivax epidemics in a population with varying degrees of G6PD deficiency].** *Med Parazitol (Mosk)* 2010:24-28.
19. Burgess RW, Bray RS: **The effect of a single dose of primaquine on the gametocytes, gametogony and sporogony of Laverania falciparum.** *Bull World Health Organ* 1961, **24:**451-456.
20. Brewer GJ, Tarlov AR, Alving AS: **Methaemoglobin reduction test: a new, simple, in vitro test for identifying primaquine-sensitivity.** *Bull World Health Organ* 1960, **22:**633-640.
21. Alving AS, Johnson CF, Tarlov AR, Brewer GJ, Kellermeyer RW, Carson PE: **Mitigation of the haemolytic effect of primaquine and enhancement of its action against exoerythrocytic forms of the Chesson strain of Piasmodium vivax by intermittent regimens of drug administration: a preliminary report.** *Bull World Health Organ* 1960, **22:**621-631.
22. Hockwald RS, Arnold J, Clayman CB, Alving AS: **Toxicity of primaquine in Negroes.** *J Am Med Assoc* 1952, **149:**1568-1570.
23. Weiss WR, Oloo AJ, Johnson A, Koech D, Hoffman SL: **Daily primaquine is effective for prophylaxis against falciparum malaria in Kenya: comparison with mefloquine, doxycycline, and chloroquine plus proguanil.** *J Infect Dis* 1995, **171:**1569-1575.
24. Vasquez AM, Sanin F, Alvarez LG, Tobon A, Rios A, Blair S: **[Therapeutic efficacy of a regimen of artesunate-mefloquine-primaquine treatment for Plasmodium falciparum malaria and treatment effects on gametocytic development].** *Biomedica* 2009, **29:**307-319.
25. El-Sayed B, El-Zaki SE, Babiker H, Gadalla N, Ageep T, Mansour F, Baraka O, Milligan P, Babiker A: **A randomized open-label trial of artesunate- sulfadoxine-pyrimethamine with or without primaquine for elimination of sub-microscopic P. falciparum parasitaemia and gametocyte carriage in eastern Sudan.** *PLoS One* 2007, **2:**e1311.
26. Shekalaghe SA, ter Braak R, Daou M, Kavishe R, van den Bijllaardt W, van den Bosch S, Koenderink JB, Luty AJ, Whitty CJ, Drakeley C, et al: **In Tanzania, hemolysis after a single dose of primaquine coadministered with an artemisinin is not restricted to glucose-6-phosphate dehydrogenase-deficient (G6PD A-) individuals.** *Antimicrob Agents Chemother* 2010, **54:**1762-1768.

27. Giao PT, de Vries PJ, Hung le Q, Binh TQ, Nam NV, Kager PA: **CV8, a new combination of dihydroartemisinin, piperaquine, trimethoprim and primaquine, compared with atovaquone-proguanil against falciparum malaria in Vietnam.** *Trop Med Int Health* 2004, **9:**209-216.
28. Kamtekar KD, Gogtay NJ, Dalvi SS, Karnad DR, Chogle AR, Aigal U, Kshirsagar NA: **A prospective study evaluating the efficacy of a single, 45-mg dose of primaquine, as a gametocytocidal agent, in patients with Plasmodium falciparum malaria in Mumbai, India.** *Ann Trop Med Parasitol* 2004, **98:**453-458.
29. Suputtamongkol Y, Chindarat S, Silpasakorn S, Chaikachonpatd S, Lim K, Chanthapakajee K, Kaewkaukul N, Thamlikitkul V: **The efficacy of combined mefloquine-artesunate versus mefloquine-primaquine on subsequent development of Plasmodium falciparum gametocytemia.** *Am J Trop Med Hyg* 2003, **68:**620-623.
30. White NJ: **Primaquine to prevent transmission of falciparum malaria.** *Lancet Infect Dis* 2012.
31. Eziefula AC, Staedke SG, Yeung S, Webb E, Kamya M, White NJ, Bousema T, Drakeley C: **Study protocol for a randomised controlled double-blinded trial of the dose-dependent efficacy and safety of primaquine for clearance of gametocytes in children with uncomplicated falciparum malaria in Uganda.** *BMJ Open* 2013, **3**.
32. Hsia YE, Miyakawa F, Baltazar J, Ching NS, Yuen J, Westwood B, Beutler E: **Frequency of glucose-6-phosphate dehydrogenase (G6PD) mutations in Chinese, Filipinos, and Laotians from Hawaii.** *Hum Genet* 1993, **92:**470-476.
33. Tiono AB, Dicko A, Ndububa DA, Agbenyega T, Pitmang S, Awobusuyi J, Pamba A, Duparc S, Goh LE, Harrell E, et al: **Chlorproguanil-dapsone-artesunate versus chlorproguanil-dapsone: a randomized, double-blind, phase III trial in African children, adolescents, and adults with uncomplicated Plasmodium falciparum malaria.** *Am J Trop Med Hyg* 2009, **81:**969-978.
34. Carter N, Pamba A, Duparc S, Waitumbi JN: **Frequency of glucose-6-phosphate dehydrogenase deficiency in malaria patients from six African countries enrolled in two randomized anti-malarial clinical trials.** *Malar J* 2011, **10:**241.
35. Baird JK, Hoffman SL: **Primaquine therapy for malaria.** *Clin Infect Dis* 2004, **39:**1336-1345.
36. Dern RJ, Beutler E, Alving AS: **The hemolytic effect of primaquine. V. Primaquine sensitivity as a manifestation of a multiple drug sensitivity.** *J Lab Clin Med* 1955, **45:**30-39.
37. Bunnag D, Harinasuta T, Pinichpongse S, Suntharasami P: **Effect of primaquine on gametocytes of Plasmodium falciparum in Thailand.** *Lancet* 1980, **2:**91.
38. Pukrittayakamee S, Chotivanich K, Chantra A, Clemens R, Looareesuwan S, White NJ: **Activities of artesunate and primaquine against asexual- and sexual-stage parasites in falciparum malaria.** *Antimicrob Agents Chemother* 2004, **48:**1329-1334.
39. Song J, Socheat D, Tan B, Dara P, Deng C, Sokunthea S, Seila S, Ou F, Jian H, Li G: **Rapid and effective malaria control in Cambodia through mass administration of artemisinin-piperaquine.** *Malar J* 2010, **9:**57.
40. Bousema T, Drakeley C: **Epidemiology and infectivity of Plasmodium falciparum and Plasmodium vivax gametocytes in relation to malaria control and elimination.** *Clin Microbiol Rev* 2011, **24:**377-410.
41. Beutler E, Mitchell M: **Special modifications of the fluorescent screening method for glucose-6-phosphate dehydrogenase deficiency.** *Blood* 1968, **32:**816-818.
42. **Overview of the management of acute kidney injury (acute renal failure)** [http://www.uptodate.com/contents/overview-of-the-management-of-acute-kidney-injury-acute-renal-failure?detectedLanguage=en&source=search_result&search=renal+failure&selectedTitle=2%7E150&provider=noProvider]
43. Ouedraogo AL, Bousema T, Schneider P, de Vlas SJ, Ilboudo-Sanogo E, Cuzin-Ouattara N, Nebie I, Roeffen W, Verhave JP, Luty AJ, Sauerwein R: **Substantial contribution of submicroscopical Plasmodium falciparum gametocyte carriage to the infectious reservoir in an area of seasonal transmission.** *PLoS One* 2009, **4:**e8410.
44. Schneider P, Schoone G, Schallig H, Verhage D, Telgt D, Eling W, Sauerwein R: **Quantification of Plasmodium falciparum gametocytes in differential stages of development by quantitative nucleic acid sequence-based amplification.** *Mol Biochem Parasitol* 2004, **137:**35-41.
45. Shekalaghe S, Drakeley C, Gosling R, Ndaro A, van Meegeren M, Enevold A, Alifrangis M, Mosha F, Sauerwein R, Bousema T: **Primaquine Clears Submicroscopic Plasmodium falciparum Gametocytes that Persist after Treatment with Sulphadoxine-Pyrimethamine and Artesunate.** *PLoSONE* 2007, **2:**e1023.
46. Okell LC, Griffin J, Kleinschmidt I, Hollingsworth TD, Churcher T, White M, Bousema T, Drakeley CJ, Ghani A: **The potential contribution of mass treatment to the control of Plasmodium falciparum malaria.** *PLoS One* 2011, **6:**e20179.

17. ROLES INVESTIGATORS

Sodiomon Sirima, MD PhD is a clinician and senior researcher and the local PI of this project. He has coordinated several clinical trials in Burkina Faso, including multiple trials with antimalarial drugs and a recent malaria vaccine trial.

Guido Bastiaens, MD is a trial clinician. He was the trial physician for several studies involving experimental malaria infections in human volunteers at RUNMC and a trial at the CNRFP. He will support data enrolment and clinical management.

Chi Eziefula, MD is a trial clinician who conducted the sister project in Uganda. She will support protocol development and advise on clinical management.

Alfred Tiono, MD, PhD is an experienced trial clinician who coordinated several clinical trials at CNRFP including a clinical trial with AL-ivermectin to interrupt malaria transmission.

Teun Bousema, PhD is the international principal investigator. He is a senior epidemiologist at RUNMC and LSHTM and has coordinated clinical trials with antimalarial drugs and malaria transmission endpoints in Kenya (2003-2004) and Tanzania (2004-2008). He will coordinate data collection and analysis.

Bronner Gonçalves, MD is a junior epidemiologist at LSHTM and has been involved in studies on gametocyte epidemiology and infectivity in Mali.

Chris Drakeley, PhD is a field epidemiologist with a long-standing track record on the assessment of transmission potential after antimalarial drug combinations.
