# Supplementary material for: Safety of single low-dose primaquine in glucose-6-phosphate dehydrogenase deficient falciparum-infected African males: Two open-label, randomized, safety trials
Source: PLoS One. 2018 Jan 11;13(1):e0190272. doi: 10.1371/journal.pone.0190272 (PMC5764271; doi:10.1371/journal.pone.0190272)

CLINICAL TRIAL PROTOCOL

Title

Evaluation of the safety of primaquine in combination with dihydroartemisinin-piperaquine in G6PD deficient males in The Gambia (SAFEPRIM-II)

Protocol No:

**SCC No:**	**1391**
**Brief Title**	SAFEPRIM- II
**Other Number(s)**	
**Protocol Version – Date**	Version 4.0 23rd May, 2016
**Sponsor**	London School of Hygiene and Tropical Medicine
	Keppel Street, London WC1E 7HT
	Tel +44 (0) 20 7927 2626
	Fax +44 (0) 20 75805636
**Chief Investigator**	Professor Chris Drakeley

Signature page

The clinical trial will be carried out in accordance with the protocol, the principles of Good Clinical Practice and in accordance to local legal and regulatory requirements.

**Chief Investigator:**	**Signature:**	**Date:**
Prof. Chris Drakeley<br>*Name*	______________________	__________________

**Site Principal Investigator:**	**Signature:**	**Date:**
Prof. Umberto D'Alessandro<br>*Name*	______________________	__________________

Protocol amendment(s)

Amendment #: 2

**Chief Investigator:**	**Signature:**	**Date:**
Prof. Chris Drakeley<br>Name		

**Site Principal Investigator:**	**Signature:**	**Date:**
Prof. Umberto D'Alessandro<br>Name		

Table of contents

Page
Signature page 2
Protocol amendment(s) 3
Key roles 6
List of abbreviations 7
Protocol summary 8
1 Background information and rationale 10
1.1 Background information 10
1.2 Rationale 12
2 Study objectives 13
2.1 Study objectives 13
2.2 Study endpoints 14
3 Study design 14
3.1 Type of study and design 14
3.2 Randomisation and blinding procedures 15
3.2.1 Randomisation 15
3.2.2 Dose escalation protocol 16
3.3 Investigational products 16
3.3.1 Description of products 16
3.3.2 Formulation, packaging and labelling 17
3.3.3 Product storage and stability 17
3.3.4 Dosage, preparation and administration of investigational products 17
3.3.5 Concomitant medications/treatments 17
4 Selection and withdrawal of participants 17
4.1 Selection of participants 17
4.2 Eligibility of participants 18
4.2.1 Inclusion criteria 18
4.2.2 Exclusion criteria 18
4.3 Withdrawal of participants 18
5 Study procedures and evaluations 19
5.1 Study schedule 19
5.1.1 Screening 19
5.1.2 Enrollment (Baseline) 19
5.1.3 Follow-up 19
5.2 Study evaluations 21
5.2.1 Clinical evaluations 21
5.2.2 Laboratory evaluations 21
5.2.3 Pharmacological analysis 23
6 Safety considerations 24
6.1 Methods and timing for assessing, recording, and analysing safety parameters 24
6.1.1 Adverse events 24
6.1.2 Management of haemolysis 25
6.1.3 Serious adverse events (SAEs) 25

6.2 Reporting procedures 26
6.3 Safety oversight 26
6.4 Assessment of causality 27
7 Discontinuation criteria 28
7.1 Participant's premature termination 28
7.2 Study discontinuation 28
8 Statistical considerations 28
8.1 Data analysis plan 29
9 Data handling and record keeping 29
9.1 Data management and processing 29
9.2 Source documents and access to source data 29
9.3 Protocol deviations 29
10 Quality control and quality assurance 30
10.1 Study monitoring 30
11 Ethical considerations 30
11.1 General considerations on human subject protection 30
11.1.1 Rationale for participant selection 30
11.1.2 Evaluation of risks and benefits 31
11.2 Informed consent 31
11.3 Participant confidentiality 31
11.4 Future use of stored specimen 32
12 Financing and insurance 32
13 Publication policy 32
14 References 33
15 Schematic of Study Design: 39

Key roles

For questions regarding this protocol, contact professor Umberto D'Alessandro at MRC Unit, The Gambia*, udalessandro@mrc.gm , phone +2204495917.*

**Author(s):**	Umberto D'Alessandro, Joseph Okebe, Teun Bousema, Chris Drakeley
**Sponsor's representative:**	Patricia Henley
**Chief Investigator(s):**	Teun Bousema (PhD), Chris Drakeley (PhD)
**Principal Investigator(s):**	Umberto D'Alessandro (PhD), Joseph Okebe (PhD candidate)
**Sub-Investigator(s):**	Guido Bastiaens (PhD candidate), Muna Affara (PhD)
**Sponsor's Medical Expert:**	TBD
**Trial monitor(s):**	**CTSO, MRCG**
**Local Safety Monitor:**	Dr Magnus Ochoge
**Chair of DMC/DMSB:**	**Prof Roly Gosling**
**Statistician:**	**TBD**
**Clinical Laboratory/ies:**	London School of Hygiene & Tropical Medicine, London UK
**Other institutions/ Collaborators:**	Radboud University medical Centre, Nijmegen, Netherlands
**Local Ethics Committee**	Gambia Government/MRC Joint Ethics Committee,<br>c/o MRC Unit, The Gambia,<br>PO Box 273, Banjul, The Gambia, West Africa

List of abbreviations

ACT	Artemisinin-based Combination Therapy
AE	Adverse Event
AQ	Amodiaquine
CRF	Case Report Form
DHA-PPQ	Dihydroartemisinin-Piperaquine
DMFA	Direct Membrane Feeding Assay
DSMB	Data Safety Monitoring Board
G6PD	Glucose- 6 - phosphate dehydrogenase
GCP	Good Clinical Practice
ICH	International Conference on Harmonization
IEC	Independent Ethics Committee
LDH	Lactate Dehydrogenase
LSHTM	London Schol of Hygiene and Tropical Medicine
MCV	Mean Corpuscular Volume
MDA	Mass Drug Administration
MRC	Medical Research Council; represents Medical Research Council Unit, The Gambia
PCR	Ploymerase Chain Reaction
PD	Protocol Deviation
PI	Principal Investigator
PQ	Primaquine
QT-NASBA	Quantitative Nucleic Acid Sequence Based Amplification
RDW	Red cell distribution width
SAE	Serious Adverse Event
SOP	Standard Operating Procedure
WHO	World Health Organisation

Protocol summary

**Title:**	Evaluation of the safety of primaquine in combination with dihydroartemisinin-piperaquine in G6PD deficient males in The Gambia (SAFEPRIM-II)
**Alias :**	SAFEPRIM- II
**Phase**:	One
**Population:**	G6PD deficient males,
**Number of participants:**	70
**Number of Sites:**	One
**Location of Sites (including satellite sites):**	Villages around the Basse MRC Field station
**Trial Duration:**<br>**- Clinical Phase:**<br>**- Whole trial:**	3 months<br>6 months
**Duration for Participants:**	28 days
**Description of Investigational Products:**	1. Primaquine (PQ) is formulated in the tablet form for oral administration. Each tablet contains 26.3 mg of Primaquine phosphate (equivalent to 15 mg of primaquine base).<br>2. Eurartesim® is formulated in the tablet form and each tablet contains 320 mg piperaquine tetraphosphate (as the tetrahydrate; PPQ) and 40mg dihydroartemisinin (DHA).
**Objectives:**	**Main**<br>To evaluate the tolerability and safety of increasing doses of PQ in combination with DHA-PPQ in G6PD deficient males.<br>**Specific objectives**<br>• Evaluate the safety and tolerability of increasing doses of PQ when administered with DHA-PPQ in G6PD deficient males compared to control groups (G6PD def. + DHA-PPQ only; G6PD normal + 0.25 mg/kg PQ; G6PD normal + 0.40 mg/kg PQ).<br>• Assess prevalence of different G6PD type mutations and safety of PQ in individuals with these different G6PD type mutations<br>• Obtain basic pharmacokinetic parameters for PQ in a G6PD deficient population

**Endpoints:**	1. **Haemoglobin concentration relative to baseline values (safety)**<br>2. Haematology abnormalities during follow-up (safety): haptoglobin, MCV, RDW and leucocytes<br>3. Biochemistry abnormalities during follow-up (safety): bilirubin, LDH, creatinine and potassium<br>4. Frequency and severity of adverse events (safety)
**Description of Study Design:**	The study is an open-label, dose-escalation Phase I trial. A minimum of 70 males ≥10 years will be sequentially enrolled into five treatment groups.<br>Two out of five treatment groups are intervention groups and three are control groups. The two intervention groups consist of 20 individuals each; doses of 0.25 and 0.4 mg/kg PQ + DHA-PPQ will be given, respectively.<br>The three control groups consist of 30 individuals: 10 male participants with G6PD deficiency will receive DHA-PPQ only, 10 males with normal G6PD enzyme function will receive 0.25 mg/kg PQ + DHA-PPQ, and 10 males with normal G6PD enzyme function will receive 0.4 mg/kg PQ + DHA-PPQ.<br>Clinical follow-up of participants and sampling will be done twice daily for the first 4 days (days 0, 1, 2 and 3) and once daily on days 4, 5, 7, 10, 14 and 28. At each time-point laboratory safety parameters are measured, including haematology, biochemistry and urine dipstick for haemoglobinuria/urobilinogen. A similar trial (SafePrim) using a similar protocol is ongoing in Burkina Faso.

1 Background information and rationale

1.1 Background information

A new global effort is underway to step up malaria control and push towards the elimination of malaria as a public health problem (1). Some substantial successes have been achieved in shrinking the global distribution of malaria. In Africa, effective elimination programmes have been initiated in Zanzibar and South Africa. There are now several African countries with a commitment to malaria elimination. This call for elimination has created a drive for the deployment of tools to reduce malaria transmission. One such tool is primaquine (PQ). It is a drug which can efficiently block the transmission of *Plasmodium falciparum* malaria from humans to mosquitoes by clearing the parasite's transmission stages, gametocytes that persist after artemisinin-combination therapy (ACT) (2, 3). A study conducted in Tanzania in symptomatic parasitized children demonstrated a dramatic reduction of gametocyte circulation time from 28.6 days with ACT alone to 6.3 days with ACT-PQ (4). A drawback of using PQ is, however, that is has been shown to cause haemolysis in glucose-6-phosphate dehydrogenase (G6PD) deficient individuals (5-7). G6PD is an essential enzyme linked to the pentose phosphate pathway; in erythrocytes, defence against oxidative stress is dependent on G6PD since they do not contain mitochondria. Its X-linked deficiency is one of world's most common genetic polymorphisms affecting 400 million people worldwide, mostly in *P. falciparum* malaria endemic areas (8).

The WHO has classified different G6PD variants according to the magnitude of the enzyme deficiency and the severity of haemolysis (9). Classes IV and V are of no clinical significance.

- Class I variants have severe enzyme deficiency (less than 10 percent of normal enzyme activity) and have chronic (nonspherocytic) hemolytic anemia.
- Class II variants, such as G6PD Mediterranean, also have severe enzyme deficiency, but there are usually only intermittent episodes of acute haemolysis associated with infection, drugs, or chemicals.
- Class III variants, such as G6PD A-, have mild to moderate enzyme deficiency (10 to 60 percent of normal enzyme activity) with intermittent episodes of acute haemolysis usually associated with infection, drugs, or chemicals.
- Class IV variants have no enzyme deficiency or haemolysis.
- Class V variants have increased enzyme activity.

The Class III A- variant is considered the most common in The Gambia, and Africa as a whole (10, 11).

In 2012, the WHO recommended that "A single 0.25 mg base/kg primaquine dose should be given to all patients with parasitologically-confirmed *P. falciparum* malaria on the first day of treatment in addition to an ACT, except for pregnant women and infants <1 year of age" (from Malaria Policy Advisory Committee Meeting 2012)(12). Before this, a single 0.75 mg/kg dose was considered standard for gametocyte clearance. However, detailed data about the lowest effective PQ dose and robust data regarding the safety of various PQ dosages in glucose-6-phosphate dehydrogenase (G6PD) deficient individuals are lacking (13). This is remarkable, since all parasitologically-confirmed *P. falciparum* malaria patients in malaria elimination areas will now receive a single dose of PQ on the first day of treatment with ACT. This includes G6PD deficient individuals, who can account up to 30% of the population in some malaria-endemic areas in Africa with a mean prevalence of approximately 10% (8, 9). Determining the safety profile of PQ in G6PD deficient individuals has become crucial and high quality clinical studies in G6PD deficient individuals are urgently needed.

Safety data for PQ use in Africa or African Americans are limited. In a large scale MDA conducted by Clyde in 1962, PQ was paired with amodiaquine (AQ) in a hyperendemic area of Tanzania. More than 15,000 subjects were studied in three clusters: weekly administration, fortnightly administration, and monthly administration. Clyde reported no safety data and it is unclear who was excluded from treatment (14). In a series of studies in G6PD deficient African-American volunteers, Alving *et al.* showed that, in three individuals, haemolysis occurred with daily administration of 30mg (approximately 0.5 mg/kg) of PQ. But, after three weeks, the haematocrit levels recovered and lower doses of PQ resulted in less haemolysis. Eight weekly doses of 60 mg and 45 mg were not associated with haemolysis (15, 16). Daily administration of 30mg of PQ to African Americans resulted in significant haemolysis in 1%, compared to no severe haemolysis when 15 mg was administered (17).

In a more recent study, Kenyan school children were randomized to receive 15mg PQ daily or three times a week as a malaria prophylactic. It is not clear whether G6PD deficient individuals were included and haemoglobin (Hb) levels are not reported but the authors note simply that "PQ was remarkably well tolerated in our studies" (18). In nearly 1500 patients studied prospectively in trials of single dose PQ given as a gametocytocide in Africa,

Asia and South-America, no severe haemolysis requiring blood transfusion was reported (3, 12, 19-24).
Based on these previously mentioned observations, the risk of haemolysis with single-dose PQ was regarded as acceptable except in areas where severe G6PD deficiency is prevalent. Shekalaghe *et al.* showed, however, that administration of a single dose of 0.75 mg/kg PQ with sulphadoxine-pyrimethamine (SP) plus artesunate (AS) treatment in 1110 individuals older than 1 year caused moderate anaemia haemolysis in 40% (6/15) of G6PD deficient individuals (A- genotype, WHO Class III variant) with mean change in Hb compared to baseline of -2.5 g/dL, but in only 4.5% (18/399) of non-deficient individuals (mean change in Hb compared to baseline of -0.5 g/dL). There was no clinical compromise due to anaemia in any of the children, except in one child in the PQ arm, whose Hb dropped from 8.3 g/dL to 4.8 g/dL. In all cases haemolysis was transient, recovering by day 14 after treatment (21).

These recent findings illustrate the lack of robust data regarding the safety of PQ administration in G6PD deficient individuals. The current WHO recommendation for PQ (single dose of 0.25 mg base/kg) is based on safety concerns that were reported in most detail by a single study using 0.75mg/kg (21) and efficacy results of lower doses of PQ in historical studies that do not all meet current standards for randomized clinical trials (12, 25, 26). A recent dose-finding trial concluded that a reduced dose of 0.4mg/kg (but not of 0.1mg/kg) is likely to be as efficacious as the 0.75mg/kg dose (27) but there is no robust efficacy data on the newly recommended 0.25mg/kg dose. Presently and trial of DHA-PPQ in combination with three dose of PQ is currently underway in The Gambia (SCC 1321) and targeting asymptomatic, *P. falciparum* infected, G6PD-normal individuals. A similar trial (SafePrim) is underway in Burkina Faso. These trials represent the spectrum of scenarios for implementing PQ and will contribute to better defining the safety and efficacy profile of PQ as an intervention to reduce transmission.

References of literature and data are listed in Section 14.

1.2 Rationale

The data behind the previously recommended dosage for a single dose of PQ, 0.75 mg/kg, dates back to the 1940s and the current recommendation of 0.25 mg/kg PQ is based on limited safety and efficacy data. From a recently concluded study in Uganda (27), there is evidence that 0.4 mg/kg and 0.75 mg/kg PQ are equally efficacious in clearing

submicroscopic gametocytes. In the era of malaria elimination, an efficacious dose of PQ should be given to parasitaemic individuals with normal G6PD activity as well as G6PD deficient individuals, who have an increased risk of PQ-induced haemolysis. Therefore, using a dose-escalation design, the current study will assess safety of PQ when given together with an ACT (in this case DHA-PPQ) in G6PD deficient individuals. The current WHO-recommended dose (0.25 mg/kg) and the lowest efficacious dose (0.4 mg/kg), as defined by the recent trial, will be tested.

The planned study complements an ongoing trial in The Gambia (SCC 1321) evaluating three different PQ dosage regimens, 0.75mg/kg, 0.4mg/kg and 0.2mg/kg, combined with DHA-PPQ, in G6PD-normal asymptomatic infection carriers. The study will recruit those excluded from this trial because they are G6PD deficient irrespective of their infection status.

2 Study objectives

2.1 Study objectives

The main objective of the trial is to evaluate the tolerability and safety of increasing doses of PQ in combination with DHA-PPQ in G6PD deficient males.

Specific objectives and hypothesis to be tested

1. To evaluate the tolerability and safety of increasing doses of PQ when administered with DHA-PPQ in G6PD deficient males compared to control groups (G6PDd + DHA-PPQ only; G6PD normal + DHA-PPQ + 0.25 mg/kg PQ; G6PD normal + DHA-PPQ + 0.40 mg/kg PQ) as measured by:

    a. frequency and magnitude of adverse events

    b. change in Hb per individual and change in mean Hb in and between cohorts and treatment groups

    c. Incidence of severe anaemia (Hb <5g/dL) during follow up

    d. evidence of black urine (haemoglobinuria; dipstick positive)

    e. requirement for blood transfusion

2. To assess prevalence of different G6PD type mutations and safety of PQ in individuals with these different G6PD type mutations

3. To obtain basic pharmacokinetic parameters for PQ in a G6PD deficient population

2.2 Study endpoints

Primary

1. Haemoglobin concentration relative to baseline values (safety)

Secondary

2. Haematology abnormalities during follow-up (safety): haptoglobin, MCV, RDW and leucocytes

3. Biochemistry abnormalities during follow-up (safety): bilirubin, LDH, creatinine and potassium

4. Frequency and severity of adverse events (safety)

3 Study design

3.1 Type of study and design

The study is an open-label, dose-escalation Phase I trial. A minimum of 70 males ≥10 years, irrespective of infection status, will be sequentially enrolled into two cohorts with five treatment groups. Two out of five treatment groups are intervention groups and 3 are control groups. The two intervention groups consist of 20 individuals each; doses of 0.25 and 0.4 mg/kg PQ + DHA-PPQ will be given, respectively. The 3 control groups consist of 30 individuals: 10 male participants with G6PD deficiency will receive DHA-PPQ only, 10 males with normal G6PD enzyme function will receive 0.25 mg/kg PQ + DHA-PPQ, and 10 males with normal G6PD enzyme function will receive 0.4 mg/kg PQ + DHA-PPQ.

The first cohort (50 persons) will consist of:

1. An intervention group with G6PD deficiency receiving 0.25 mg/kg PQ + DHA-PPQ (n=20)

2. A control group with G6PD deficiency receiving DHA-PPQ only (n=10)

3. A control group with normal G6PD enzyme function receiving 0.25 mg/kg PQ + DHA-PPQ (n=10)

4. A control group with normal G6PD enzyme function receiving 0.4 mg/kg PQ + DHA-PPQ (n=10)

The second cohort (20 persons) will consist of:

1. An intervention group with G6PD deficiency receiving 0.4 mg/kg PQ + DHA-PPQ

3.2 Randomisation and blinding procedures

3.2.1 Randomisation

This is an open label study, randomisation procedures are limited to cohort 1. Enrollment will follow a dose-escalation protocol starting from Cohort 1 (0.25mg/kg PQ). Treatment allocation in this cohort will be by computer-generated randomization sequence placed in sealed opaque envelopes. If G6PD deficiency test indicates reduced enzyme function (<30% of normal function (28)), individuals will be enrolled in the intervention group (receiving 0.25 mg/kg PQ + DHA-PPQ) or control group (receiving DHA-PPQ only), or - if the first cohort has been completed – they will be enrolled without randomization in the intervention group receiving 0.4 mg/kg PQ + DHA-PPQ (details in table 2). If G6PD activity is normal, individuals will be enrolled in one of the other two control groups in cohort 1 (0.25 mg/kg PQ or 0.40 mg/kg PQ; details in table below).

Table 1: ***Study cohorts and dose escalation schedule***

Cohort 1	Intervention (n=20)	Control (n=10)	Control (n=10)	Control (n=10)
	0.25 mg/kg PQ + DHA-PPQ, G6PD def.	DHA-PPQ, G6PD def.	0.25 mg/kg PQ+DHA-PPQ, G6PD normal	0.4 mg/kg PQ+DHA-PPQ, G6PD normal
	Randomization = 2:1		Randomization = 1:1	
Cohort 2	Intervention (n=20)			
	0.4 mg/kg PQ + DHA-PPQ, G6PD def.			

3.2.2 Dose escalation protocol

After enrolment, participants will be assigned to the lowest-dose open cohort, with enrolment in the second cohort initiated after tolerability and short-term safety is demonstrated at the preceding lower dose (enrolment to the second cohort accounts for G6PD deficient participants only). Within each cohort, the first two participants of the intervention group are treated and monitored for six days for immediate side-effects and haematological abnormalities before the rest of the participants of that particular intervention group are enrolled and treated. Once safety of these first two participants is confirmed, the next 4 subjects are enrolled and treatment for the next four subjects is initiated on day 2 of the last treated participant of the preceding four subjects. The last two groups for each intervention group comprise five individuals, making a total of 20 (2, 4, 4, 5, and 5). After inclusion of the intervention group of the first cohort (n=20) is completed (follow-up day 14 of last participant in that group), a 10-day safety observation period is installed before enrolment of the intervention group of the second cohort is initiated.

3.3 Investigational products

3.3.1 Description of products

Two interventional products will be used in this trial: Eurartesim® (Dihydroartemisin – Piperaquine) and Primaquine (Primaquine Phosphate).

Eurartesim® is an ACT containing piperaquine tetraphosphate (as the tetrahydrate; PPQ) and dihydroartemisinin (DHA) in 8:1 ratio. It is indicated for the treatment of uncomplicated *P. falciparum* malaria in adults, children and infants ≥6 months old and weighing ≥5 kg.

Primaquine is an 8-aminoquinoline anti-protozoal agent containing primaquine phosphate with potent anti-malarial activity against the exo-erythrocytic stages of *P. vivax* and *P. ovale*, and against the primary exo-erythrocytic and mature gametocyte stages of *P. falciparum*.

3.3.2 Formulation, packaging and labelling

Eurartesim® is formulated as a white oblong biconvex film-coated scored tablet containing 320mg piperaquine tetraphosphate (as the tetrahydrate; PPQ) and 40mg dihydroartemisinin (DHA) and marked on one side with the letters “S” and “T”.

Primaquine is formulated in the tablet form for oral administration. Each tablet contains 26.3 mg of Primaquine phosphate (equivalent to 15 mg of primaquine base) packaged in bottles of 100 with ‘W’ imprinted on one side and P97 on the other.

3.3.3 Product storage and stability

The products will stored between 15 and 25°C in their original packages to protect from light and moisture.

3.3.4 Dosage, preparation and administration of investigational products

All participants will be treated with dihydroartemisinin-piperaquine (Euratesim®; Sigma Tau) administered as 3 tablets (40mg PPQ, 320mg DHA) in a once daily regimen for three days. For all groups except the G6PD deficient group receiving only DHA-PPQ (n=10), the first dose is given together with 0.25 or 0.4 mg/kg PQ.

All treatment is administered under supervision without food. All doses are given at the clinic under supervision of the study nurse. For each dose the study nurse will document whether the treatment has been given or not, the number of tablets given and whether or not the dose was vomited or repeated.

For participants who develop anaemia (Hb <11 g/dL) during the study, we will follow national treatment guidelines.

3.3.5 Concomitant medications/treatments

All drugs outside the interventional products given to any participant during the entire study duration (day 0-28) will be documented on the appropriate section of the participant record stating the name, dosage, date and time of administration of such drug(s).

4 Selection and withdrawal of participants

4.1 Selection of participants

Participants will be drawn from the villages in the catchment area of the Basse health Centre. Males within the required age group will be invited to the health centre for an informed consent and full screening and Hb status.

4.2 Eligibility of participants

Participants must meet all of the inclusion criteria and none of the exclusion criteria to be eligible to participate in the trial.

4.2.1 Inclusion criteria

- Male gender
- Age ≥10 years
- G6PD deficiency by fluorescent Spot test (for intervention groups and control group receiving DHA-PPQ only (N=50)
- G6PD normal activity by fluorescent Spot test for control groups (N=20)
- Informed consent by participant or caregiver (an assent is required for those 12-17 years)

4.2.2 Exclusion criteria

- Enrolled in another clinical trial
- Fever: temperature >37.5°C (axillary) or history of fever in the last 24 hours
- Evidence of severe illness or active infection other than malaria
- Known allergy to study medications
- Hb <11 g/dL
- Antimalarials taken within the last 2 weeks
- PQ taken within the last 4 weeks and blood transfusion within the last 90 days
- Current use of tuberculosis or anti-retroviral medication, sulphonamides, dapsone, nitrofurantoin, nalidixic acid, ciprofloxacin, methylene blue, toluidine blue, phenazopyridine and co-trimoxazole.
- History of severe chronic illness

4.3 Withdrawal of participants

A study participant will be discontinued from participation in the study if any clinically significant adverse event (AE), laboratory abnormality, intercurrent illness, or other medical condition or situation occurs such that continued participation in the study would not be in the best interest of the participant.
Participants are free to withdraw from the study at any time without giving a reason.

5 Study procedures and evaluations

For an overview see Table 2 and annex "Schema of study design".

5.1 Study schedule

5.1.1 Screening

An initial screening of males aged ≥10 years in the villages around study clinic will be conducted by the study team. Villages and individuals will be selected by convenience sampling: anyone available at the time of visit of the trial team and willing to participate. A brief information on the study and the reasons for screening for G6PD will be provided to interested individuals and we will obtain a verbal consent for a fingerprick blood sample for G6PD testing. Results of their G6PD status will be fed back to all volunteers. Due to the structure of trial recruitment (see section 3.3.2), individuals will be informed that they will be contacted at a later date for participation.

5.1.2 Enrollment (Baseline)

During enrolment procedures, participants/caregivers will be informed about the objectives and practical consequences of participation, the possibility of withdrawal from the study, at any time and without any declaration of the reason. They will then be invited to sign the written consent form to participate in the trial as well as a consent for the future use of biological specimens obtained during the course of the study. If the participant or caregiver is unable to write, their fingerprint will be used in substitute for a signature, and a signature from an impartial witness to the informed consent discussion will be obtained. Two copies of the consent form must be signed; a copy will be given to the participant/caregiver and the other retained by the trial team.

5.1.3 Follow-up

After initiation of treatment, participants are asked to return to the clinic on the evening of day 0, twice daily on days 1, 2, and 3, and once daily on days 4, 5, 7, 10, 14 and 28. On days 0, 1 and 2 study drugs are administered once daily. During screening and on each scheduled visit day, participants will have a Hb sample taken by finger prick. Hb concentration is determined by HemoCue photometer [Angelholm, Sweden].

A blood slide will be taken once daily on all days - except day 4 and 5 - to determine the malaria infection status (malaria infection at enrolment is not an exclusion criterion and participants may be infected during follow up). A longer follow-up is not needed since the

study is purely designed to determine safety in the first four weeks after initiation of treatment in G6PD deficient individuals.

An RNA sample for gametocyte detection by quantitative nucleic acid sequence based amplification (QT-NASBA) will be collected on days 0, 2, 3, 7, 10 and 14. Biochemistry (ASAT, ALAT, total Bilurubin, Creatinin) and full blood count (including reticulocytes) will be done for all individuals twice daily on days 0 (enrolment) and 1, and once daily on days 3, 7, 14 and 28. A 1.3ml whole blood sample will also be collected on Day 0 to determine G6PD genotype and mutations in the CYP450 genes (retrospectively) which may influence PQ metabolism and therefore haemolysis. Filter paper blood samples will be collected at screening and on day 14 for quantification of G6PD activity.

Urine dipstick will be done twice daily on days 0, 1, 2 and 3 and once daily on days 4, 5, 7 and 28.

Table 2: Sampling and visit schedule

	Screening	Outpatient visits														Unscheduled
Day of follow-up	0	0		1		2		3		4	5	7	10	14	28	
		M	E	M	E	M	E	M	E							
**<u>CLINICAL:</u>**																
History		X	X	X	X	X	X	X	X	X	X	X	X	X	X	X
Axillary temperature		X	X	X	X	X	X	X	X	X	X	X	X	X	X	X
Physical examination		X	X	X	X	X	X	X	X	X	X	X	X	X		X
Assessment for adverse events		X	X	X	X	X	X	X	X	X	X	X	X	X	X	X
Complete case record form		X	X	X	X	X	X	X	X	X	X	X	X	X	X	X
**<u>TREATMENT:</u>**																
DHA-PPQ<br>PQ		X<br>x		X		X										
**<u>LAB TESTING:</u>**																
Blood smear	X	X		X		X		X				X	X	X	X	X‡
RNA sample (QT-NASBA)		X				X		X				X	X	X		
Haemoglobin (Hemocue®)	X	X	X	X	X	X	X	X	X	X	X	X	X	X	X	X
Biochemistry / full blood count		X	X	X	X			X				X		X	X	X‡
Urine dipstick		X	X	X	X	X	X	X	X	X	X	X			X	X‡
G6PD screen (filter paper)<br>G6PD genotype/ CYP2D6	X<br>X													X		

‡on indication

5.2 Study evaluations

5.2.1 Clinical evaluations

After initiation of treatment, participants are asked to return to the clinic on the evening of day 0, twice daily on days 1, 2, and 3, and once daily on days 4, 5, 7, 10, 14 and 28. On these visits, participants will be examined clinically (except for day 28) and a structured questionnaire is used to determine the occurrence of side effects

5.2.2 Laboratory evaluations

<u>Parasite detection</u>

Blood smears are stained with 10% Giemsa for 10 minutes and then screened for asexual parasites and gametocytes on days 0, 1, 2, 3, 7, 10, 14 and 28 after initiation of treatment. Slides are double-read and read separately for asexual parasites and gametocytes in 100 high power fields.

Parasite detection by Pfs25 QT-NASBA will be done using a Biorad CFX-96 analyser. Nucleic acids are extracted manually from 50µL blood samples using the Boom method (29), using commercial lysis buffers (Severn Biotech). The *Pfs25* QT-NASBA technique is gametocyte specific and has an approximate detection limit of 0.1 gametocytes/µL in the blood volume taken. NucliSens Basic kits are used for amplification in accordance with the manufacturer's instructions. A standard dilution series of mature, *in vitro* cultured NF54 gametocytes (30) is included in each run. Gametocyte detection by *Pfs25* QT-NASBA is done for all samples on days 0, 2, 3, 7, 10 and 14 after initiation of treatment. Day 28 is excluded from this analysis because of a high chance that gametocyte prevalence on this day may be due to re-infections (31).

Haematology and Biochemistry

The HemoCue® produces a point-of-care result and at all time points, this will be used to assess Hb and clinical care based on the result. This is done in order to avoid discrepancies that could arise between HemoCue® and full blood count Hb results. A venous sample will be taken twice daily on days 0 (enrolment) and 1, and once daily on days 3, 7, 14 and 28 for a full blood count (e.g. Hb, white blood cell count, reticulocytes, platelets and haematocrit) and biochemistry (renal and liver function tests). Additional samples may be taken during unscheduled visits for clinical care or investigating an adverse event. A urinalysis will be done where indicated for investigating haemolysis.

G6PD deficiency screening

Initial (pre-enrollment) qualitative screening for G6PD will be performed using the G-6-PD OSMMR 2000 kit [R&D Diagnostics]. A sample will be collected for retrospective determination of G6PD genotype and the presence of mutations in the CYP450 genes which may influence PQ metabolism and hence haemolysis.

For the above investigations, about four mls of venous blood will be required daily on days 0-3 while on days 4, 5, 7, 10, 14 and 28, about 2-3 mls of blood will be collected at each visit. To minimise the pain from repeated needle pricks, an intravenous catether will be inserted during the first 4 days to help with the blood collection and direct draws using a needle will be performed on other visit days.

5.2.3 Pharmacological analysis

Pharmacokinetic evaluations will be obtained on approximately one quarter of the participants in intervention groups with G6PD deficiency, a maximum of 10 G6PD deficient participants

The pharmacokinetic sampling will involve taking a total of 7 venous blood samples of less than 1ml. The total amount sampled, being approximately 7 mL in 3 days. The first sample is just prior to the PQ dose (a baseline sample) and the subsequent six samples are at intervals up to 72 hours after the dose of PQ. The blood samples will be taken at fixed times between 8am to 5pm. The first 5 samples are taken on day 0 and they will be taken through an intravenous catheter, placed when the baseline pharmacokinetic sample is taken. If a catheter is not placed successfully, a butterfly needle may be used. The last two samples (one on day 1 and one on day 2) will be taken by individual blood draws (venipuncture). The participant will be asked to stay in the clinic between sampling times on day 0.

In order to minimize the total number of blood draws per participant, the sampling timeframe has been randomized so that over the total population of participants, a population pharmacokinetic model can be constructed for analysis. Six randomized sample times will be allocated to sequential consenting participants in opaque envelopes. Each sample time is within a window so that there are 5 samples on day 0 and one each on days 1 and 2. Pharmacokinetic samples will be analysed in Professor Niklas Lindegardh's laboratory in Mahidol University, Bangkok, Thailand, where the randomized sampling framework will be generated.

<u>Selection for pharmacokinetic analysis</u>

On day 0, every 4th G6PD deficient adult who was enrolled in an intervention group will be invited to participate in pharmacokinetic sampling. If this study participant declines inclusion in the pharmacokinetic study, the next consecutive participant will be invited. If a participant is included and vomits within 30 minutes of primaquine dose, the next participant will be invited. All participants undergoing pharmacokinetic sampling must satisfy the following criteria in addition to the study's inclusion criteria:

Pharmacokinetic analysis inclusion criteria:

1. Siting of secure blood sampling access feasible (venflon/ butterfly needle) on day 0
2. Willing and able to attend study clinic by 8:00am on days 0-2
3. Willing to stay on study clinic premises between 8am to 5pm on day 0

Study subjects included in the pharmacokinetic analysis will be seen by a study clinician who will select the next available pharmacokinetic opaque envelope. This will contain a sheet with the sampling times for the participant. The clinician will label this sheet with the participant's study number. The clinician will be responsible for adhering to the sampling times.

The study nurse will fix venous access (using a venflon or butterfly) and take the baseline sample. All blood will be taken into heparinised tubes. The subsequent 4 samples will be taken at the allocated sampling times then the fixed venous access will be removed. The participant will be informed as to what time they need to present by on days 1 and 2 for the last two samples. On these days, the sample will be taken by phlebotomy with no fixed access.

6 Safety considerations

6.1 Methods and timing for assessing, recording, and analysing safety parameters

For clinical evaluations, see section 5.2.1

6.1.1 Adverse events

Adverse events are defined as any undesirable medical experience occurring to a subject during the study, whether or not considered related to the trial. An AE can therefore be any unfavourable and unintended sign (including an abnormal laboratory finding), symptom, or disease (new or exacerbated) temporally associated with a study intervention. AEs may include events that occur as a result of protocol-mandated procedures (e.g. venepunctures). All AEs reported spontaneously by the subject or observed by the trial clinicians or their staff will be recorded.

Due to the unique situation in this trial where the intervention is noted to cause some degree of haemolysis, only drops in Hb >5g/dl from baseline will be noted as an AE. Other abnormal laboratory findings that are judged by the trial clinicians to be clinically significant will be also recorded as AEs or serious adverse events (SAEs) if they meet the definition. The principal investigators and trial clinicians, supported by the Local Safety Monitor will exercise their medical and scientific judgement in taking these decisions.

6.1.2 Management of haemolysis

The main risk in this trial is represented by the hematologic toxicity of PQ, which is mainly but not exclusively limited to G6PD deficient individuals. Some individuals enrolled in this trial might carry malaria parasites and asymptomatic malaria infection is expected to precipitate a small degree of haemolysis, due to erythrocyte parasitisation. However, given that the trial involves testing low doses of PQ than those previously associated with haemolysis and *that PQ is given* at a single time-point, we do not expect significant haemolysis to occur at any point during the study. Furthermore, the dose-escalation schedule and halting criteria allow us to conduct this trial safely and will minimise the risk of haemolysis.

However, for the purposes of systematic and responsible safety monitoring, the following detailed protocols have been developed for the management of participants in whom haemolysis is suspected.

Haemolysis will be diagnosed *by* detailing the size of haemoglobin fall (measured by Hemocue®) after PQ treatment and the absolute haemoglobin value. In addition, any participant presenting with or complaining of tea-coloured or black urine will be assessed for haemolysis.

The trial clinician will assess any participant reporting black urine. Black urine is defined as any dark-coloured urine (not orange). A full clinical exam and history will be obtained, a dipstick urine sample will be done for evidence of haematuria and proteinuria and a venous blood sample drawn for assessment of full blood count. A blood film will also be assessed for abnormal red blood cells (schistocytes). The trial clinician, considering the size and rate of Hb drop and signs of clinical compromise, will assess requirement for blood transfusion.

In summary, participants with evidence of mild haemolysis (Hb 8-10g/dl) will be observed and monitored with regular Hb checks during visits. Participants will be started on haematinics. If Hb drops to ≤5g/dl while participant is asymptomatic or >8g/dl but complaining of symptoms such as weakness or tiredness, they will be considered for blood transfusion. Blood for transfusion will be screened for HIV and hepatitis B virus infection.

6.1.3 Serious adverse events (SAEs)

A **serious adverse event** is any AE that is life-threatening or results in death or requires hospitalisation or prolongation of hospitalisation, is a persistent or significant disability/ incapacity or is a congenital anomaly/birth defect
Medical judgment will be exercised in deciding whether reporting is appropriate in other situations, such as important medical events that may not be immediately life-threatening or result in death or hospitalization but may jeopardize the subject or may require medical intervention to prevent one of the other outcomes listed in the above definition. These would also be considered serious. Examples of such events are intensive treatment in an emergency room or at home for allergic bronchospasm, blood dyscrasias or convulsions that do not result in hospitalization.
In this trial, a select number of participants will require whole-day hospital stays as part of their trial procedures. These would not be regarded as SAEs.

-

6.2 Reporting procedures

All SAEs will be reported to the Sponsor, the local safety monitor (LSM) and to the CTSM immediately but not later than within 24 hrs of the site being made aware of the event.

This first report may be verbal or an informal notification by email stating the trial, participant's ID, the event and the reporter. The first report will be followed promptly – (within 2 working days) - with a detailed, written initial report using the MRC Unit's Serious Adverse Event Report form. Other relevant documents (e.g. medical record progress notes, discharge summary, laboratory and diagnostic test results, X-ray findings, autopsy reports, etc) will be attached as appropriate. Follow-up reports will be sent as soon as when new relevant information becomes available. Reporting of SAEs to the Local Ethics Committee and National Regulatory Medical Authority will follow the MRC Gambia Unit's Standard Operating Procedures (SOP-CTS-009).

6.3 Safety oversight

Safety assessments will be performed, and recorded by the trial clinician. All AEs, observed by the trial clinician or by the subject, will be accurately documented in the case report form. For each event the following details will be recorded:

- Description of the event(s)
- Date and time of occurrence
- Duration
- Intensity
- Relationship with the intervention
- Action taken, including treatment
- Outcome

All AEs except fever will be judged for their intensity according to the following scale:

- Mild (grade 1): awareness of symptoms that are easily tolerated and do not interfere with usual daily activity
- Moderate (grade 2): discomfort that interferes with or limits usual daily activity
- Severe (grade 3): disabling, with subsequent inability to perform usual daily activity, resulting in absence or required bed rest
- Life-threatening (grade 4): extreme limitation in activity, significant assistance required; significant medical intervention/therapy required, hospitalization required

For details regarding the grading of symptoms we refer to the NIH Division of Microbiology and Infectious Diseases Adult Toxicity Table:
http://www.niaid.nih.gov/LabsAndResources/resources/DMIDClinRsrch/Documents/dmidadulttox.pdf

For fever, the following scale will be used:

- Mild (grade 1): 37.5 - 38.0°C
- Moderate (grade 2): > 38.0 - 39.0°C
- Severe (grade 3): > 39.0°C

If an AE changes in frequency or intensity during the specified reporting period, the previous description of the AE will be updated.

When an AE/SAE occurs, it is the responsibility of the trial clinician to review all documentation (e.g. hospital progress notes, laboratory, and diagnostics reports) related to the event. The trial clinician will then record all relevant information regarding an AE/SAE on the CRF or SAE Report Form, respectively. Furthermore, the trial clinician will attempt to establish a diagnosis of the event based on signs, symptoms, and/or other clinical information. In such cases, the diagnosis should be documented as the AE/SAE and not the individual signs/symptoms.

6.4 Assessment of causality

The trial clinician and principal investigators will assess the relationship between study procedures and the occurrence of each AE/SAE and will use clinical judgment to determine the relationship. Alternative causes, such as natural history of the underlying diseases, concomitant therapy, other risk factors and the temporal relationship of the event to the study procedures will be considered and investigated. The relationship of the AE with the study procedures will be categorized as:

Definite	An AE that follows a clear cut temporal association with a positive re-challenge test of laboratory confirmation
Probable	An AE that follows a reasonable temporal sequence from the study procedures and cannot be reasonably explained by the known characteristics of the subject's clinical state.
Possible	An AE for which insufficient information exists to indicate a high improbability that the event is related to the study procedures.
Unlikely	An AE that temporal association is improbable, but not impossible; other aetiologies such as concomitant medications or conditions, or participant's known clinical state provide plausible explanations
Not related	An event for which sufficient information exists to indicate that the etiology is unrelated either because of the temporal sequence of events or because of the subject's clinical state or other therapies.

7 Discontinuation criteria

7.1 Participant's premature termination

If an enrolled participant discontinues the study for reasons unrelated to treatment before completing the final visit on day 28, an additional participant will be enrolled in the same group. This process will be repeated until either both cohorts have their follow-up completed or enrolment to a subsequent cohort is suspended because stopping criteria are met.

7.2 Study discontinuation

The following scenarios are considered potential trial stopping criteria:

1. Any drop in Hb below 5 g/dL from Day 0 onwards
2. Any need for blood transfusion
3. Three or more participants have a drop in Hb >4 g/dL from Day 0 onwards
4. Any severe adverse event defined as any untoward medical occurence or effect that at any dose: results in death, is life threatening, requires hospitalization (attributable to the study drug), results in persistent or significant disability or incapacity.

The data safety monitoring board (DSMB) will be notified with 24 hours of the study site becoming aware of any of such this event and will prompt an urgent review to consider whether it is appropriate to stop the trial.

The DSMB may decide to put the study on hold based on adverse events, pending discussion with the principal investigators / trial clinicians / Ethics Committee(s). Following discussion, it may be decided to terminate the study. The study may also be discontinued on the advice of the principal investigators, trial clinician or the Ethics Committee(s).

8 Statistical considerations

For each of the 2 intervention groups (PQ in G6PD deficients) 20 individuals will be enrolled. This number of individuals per group will give us a 88% probability of detecting at least one severe event and ~61% probability of detecting at least 2 severe adverse events if we assume that the probability of a serious adverse event is 10% for each individual. The control groups are included to support the interpretation of haemoglobin concentrations following treatment (i.e. a reference group where a reduction in Hb is not due to primaquine in combination with low G6PD enzyme activity); the size of these control groups was based on expert opinion (WRAIR, Tafenoquine group) and not based on sample size calculations.

8.1 Data analysis plan

The primary safety outcome, risk of haemolysis, defined as the proportion of individuals in each group that has a drop of >2.5 g/dL in haemoglobin levels, will be calculated. Mean (± SD) maximal fall (±) in Hb (haemoglobin, g/dl) from enrolment to day 28 of follow-up, follow-up day of Hb nadir, maximal percentage fall in Hb level compared to enrolment value, and evidence of black urine (urine dipstick positive for Hb) will be determined. The incidence of serious adverse events by sign, symptom, laboratory parameter and relationship to taking study drug will also be determined.

9 Data handling and record keeping

9.1 Data management and processing

A local independent data monitor will undertake data monitoring at site (e.g. source data verification). A data management plan will be developed to guide these activities.

9.2 Source documents and access to source data

The Principal Investigators will maintain appropriate medical and research records for this study in compliance with the principles of good clinical practice and regulatory and institutional requirements for the protection of confidentiality of participants. The study team members will have access to records.

The authorised representatives of the Sponsor, the ethics committee(s) may inspect all documents and records required to be maintained by the investigator, including but not limited to, medical records (office, clinic, or hospital) for the participants in this study. The clinical study site will permit access to such records.

9.3 Protocol deviations

A protocol deviation (PD) is any noncompliance with the clinical trial protocol, good clinical practice (GCP), or other applicable regulatory requirements. The noncompliance may be either on the part of the participant or the investigator including the study team members, and may result in significant added risk to the study participant. As a result of a deviation, corrective actions will be developed and implemented promptly.

If a deviation from, or a change of, the protocol is implemented to eliminate an immediate hazard(s) to trial participant without prior ethics approval, the PI or designee will submit the implemented deviation or change, the reasons for it, and, if appropriate, the proposed protocol amendment(s) as soon as possible to the sponsor for agreement and the relevant ethics committee for review and approval.

The PI or designee will document and explain any deviation from the approved protocol on the CRF, where appropriate, and record and explain any deviation in a protocol deviation form that will be maintained as an essential document.

10 QUALITY CONTROL AND QUALITY ASSURANCE

10.1 Study monitoring

The study will be monitored by a monitor that will be appointed in consultation with the sponsor.

11 Ethical considerations

This study is conducted in accordance with the principles set forth in the ICH Harmonised Tripartite Guideline for Good Clinical Practice and the Declaration of Helsinki in its current version, whichever affords the greater protection to the participants.

11.1 General considerations on human subject protection

11.1.1 Rationale for participant selection

This study includes G6PD deficient individuals who are at an increased risk of haemolytic side-effects of PQ as well as individuals with normal G6PD function. Within the G6PD deficient population, safety of the DHA-PPQ plus PQ combination will be studied thoroughly. This information is essential since all parasitologically-confirmed *P. falciparum* malaria patients in elimination programmes will now receive a single dose of PQ on the first day of treatment with ACT according to new WHO policy guidelines. This includes G6PD deficient individuals who account for up to 30% of the population in some malaria-endemic areas (8, 9).

11.1.2 Evaluation of risks and benefits

The individual participant will benefit from treatment if they are found to have a malaria infection. The treatment given, DHA-PPQ plus PQ, is recommended by the WHO for elimination settings although the PQ component provides no immediate benefit to the individual. At a population level, treatment with PQ can reduce malaria transmission (12) and lead to a lower risk of infection experienced by individual community members (32). This study is an essential requirement before transmission-reducing interventions with community benefit can be implemented.

The current study will restrict enrolment to males with normal Hb values. Careful monitoring during 28 days will minimize the risk of participation for individuals. Clinical staff will receive training on standardised protocols for the detection and management of haemolysis. Blood sampling will be restricted to the absolute minimum to answer the study questions, but may be increased in some individuals to monitor safety parameters.

11.2 Informed consent

Participants and their carers will be informed about the objectives and practical consequences of participation in the study, the possibility of withdrawal from the study, at any time and without any declaration of the reason. They will then be invited to sign the written consent (assent) form to participate in this clinical trial; participants/carers will also be invited to sign consent for the future use of biological specimens obtained during the course of the study. If the participant/carer is unable to write, their fingerprint will be used in substitute for a signature, and a signature from an impartial witness to the informed consent discussion will be obtained. Two copies of the consent form will be signed.

Participants will be given a copy of the signed consent and the study information sheet, containing contact names and telephone numbers to use if they have further questions regarding the study or follow up procedures.

11.3 Participant confidentiality

All participant records are to be used only for the purpose of this research project. Names will not appear on labels on laboratory specimens or in any report resulting from the study.

Identifying information will be kept in a metal cabinet that is locked and only accessible to the study clinician or his clinical representative in case he is absent. Paper forms are only accessible to senior research staff (i.e. clinician, nurse and scientific personnel); names and addresses will be removed from digital files which will be password protected. All materials

collected in this study will be labelled with a study identification number that cannot be directly linked to identifying information.

11.4 Future use of stored specimen

Stored samples will be used to determine G6PD genotype and mutations in the CYP450 genes retrospectively, which may influence PQ metabolism and therefore haemolysis. DNA samples may be archived for future sequencing of the G6PD and CYP genes.

12 Financing and insurance

The trial Sponsor is responsible for financing trial activities and will provide indemnity for participants according to its indemnity statement.

13 Publication policy

Study findings and protocol will be published in leading international scientific journals and will be freely available on-line.
A summary report will be submitted to the Sponsor at the end of the study.

14 References

1. World Health Organization. Malaria elimination: a field manual for low and moderate endemic countries. 2007.
2. Shekalaghe S, Drakeley C, Gosling R, Ndaro A, van Meegeren M, Enevold A, et al. Primaquine clears submicroscopic Plasmodium falciparum gametocytes that persist after treatment with sulphadoxine-pyrimethamine and artesunate. PloS one. 2007;2(10):e1023. PubMed PMID: 17925871. Pubmed Central PMCID: 1995753. Epub 2007/10/11. eng.
3. Smithuis F, Kyaw MK, Phe O, Win T, Aung PP, Oo AP, et al. Effectiveness of five artemisinin combination regimens with or without primaquine in uncomplicated falciparum malaria: an open-label randomised trial. The Lancet infectious diseases. 2010 Oct;10(10):673-81. PubMed PMID: 20832366. Pubmed Central PMCID: 2947715. Epub 2010/09/14. eng.
4. Bousema T, Okell L, Shekalaghe S, Griffin JT, Omar S, Sawa P, et al. Revisiting the circulation time of Plasmodium falciparum gametocytes: molecular detection methods to estimate the duration of gametocyte carriage and the effect of gametocytocidal drugs. Malaria journal. 2010;9:136. PubMed PMID: 20497536. Pubmed Central PMCID: 2881938. Epub 2010/05/26. eng.
5. Abeyaratne KP, Halpe NL. Sensitivity to primaquine in Ceylonese children due to deficiency of erythrocytic glucose-6-phosphate dehydrogenase. The Ceylon medical journal. 1968 Sep;13(3):134-8. PubMed PMID: 5721557. Epub 1968/09/01. eng.
6. Reeve PA, Toaliu H, Kaneko A, Hall JJ, Ganczakowski M. Acute intravascular haemolysis in Vanuatu following a single dose of primaquine in individuals with glucose-6-phosphate dehydrogenase deficiency. The Journal of tropical medicine and hygiene. 1992 Oct;95(5):349-51. PubMed PMID: 1404560. Epub 1992/10/01. eng.
7. Khoo KK. The treatment of malaria in glucose-6-phosphate dehydrogenase deficient patients in Sabah. Annals of tropical medicine and parasitology. 1981 Dec;75(6):591-5. PubMed PMID: 7325735. Epub 1981/12/01. eng.
8. Cappellini MD, Fiorelli G. Glucose-6-phosphate dehydrogenase deficiency. Lancet. 2008 Jan 5;371(9606):64-74. PubMed PMID: 18177777. Epub 2008/01/08. eng.
9. Beutler E. G6PD deficiency. Blood. 1994 Dec 1;84(11):3613-36. PubMed PMID: 7949118. Epub 1994/12/01. eng.
10. Tiono AB, Dicko A, Ndububa DA, Agbenyega T, Pitmang S, Awobusuyi J, et al. Chlorproguanil-dapsone-artesunate versus chlorproguanil-dapsone: a randomized, double-blind, phase III trial in African children, adolescents, and adults with uncomplicated Plasmodium falciparum malaria. The American journal of tropical medicine and hygiene. 2009 Dec;81(6):969-78. PubMed PMID: 19996424. Epub 2009/12/10. eng.
11. Carter N, Pamba A, Duparc S, Waitumbi JN. Frequency of glucose-6-phosphate dehydrogenase deficiency in malaria patients from six African countries enrolled in two randomized anti-malarial clinical trials. Malaria journal. 2011;10:241. PubMed PMID: 21849081. Pubmed Central PMCID: 3188486. Epub 2011/08/19. eng.

12. White NJ. Primaquine to prevent transmission of falciparum malaria. The Lancet infectious diseases. 2013 Feb;13(2):175-81. PubMed PMID: 23182932. Epub 2012/11/28. eng.
13. Eziefula AC, Gosling R, Hwang J, Hsiang MS, Bousema T, von Seidlein L, et al. Rationale for short course primaquine in Africa to interrupt malaria transmission. Malaria journal. 2012;11:360. PubMed PMID: 23130957. Pubmed Central PMCID: 3502539. Epub 2012/11/08. eng.
14. Clyde DF. Mass administration of an antimalarial drug combining 4-aminoquinoline and 8-aminoquinoline in Tanganyika. Bulletin of the World Health Organization. 1962;27:203-12. PubMed PMID: 14021798. Pubmed Central PMCID: 2555798. Epub 1962/01/01. eng.
15. Brewer GJ, Tarlov AR, Alving AS. Methaemoglobin reduction test: a new, simple, in vitro test for identifying primaquine-sensitivity. Bulletin of the World Health Organization. 1960;22:633-40. PubMed PMID: 13804322. Pubmed Central PMCID: 2555344. Epub 1960/01/01. eng.
16. Alving AS, Johnson CF, Tarlov AR, Brewer GJ, Kellermeyer RW, Carson PE. Mitigation of the haemolytic effect of primaquine and enhancement of its action against exoerythrocytic forms of the Chesson strain of Piasmodium vivax by intermittent regimens of drug administration: a preliminary report. Bulletin of the World Health Organization. 1960;22:621-31. PubMed PMID: 13793053. Pubmed Central PMCID: 2555355. Epub 1960/01/01. eng.
17. Hockwald RS, Arnold J, Clayman CB, Alving AS. Toxicity of primaquine in Negroes. Journal of the American Medical Association. 1952 Aug 23;149(17):1568-70. PubMed PMID: 14945981. Epub 1952/08/23. eng.
18. Weiss WR, Oloo AJ, Johnson A, Koech D, Hoffman SL. Daily primaquine is effective for prophylaxis against falciparum malaria in Kenya: comparison with mefloquine, doxycycline, and chloroquine plus proguanil. The Journal of infectious diseases. 1995 Jun;171(6):1569-75. PubMed PMID: 7769294. Epub 1995/06/01. eng.
19. Vasquez AM, Sanin F, Alvarez LG, Tobon A, Rios A, Blair S. [Therapeutic efficacy of a regimen of artesunate-mefloquine-primaquine treatment for Plasmodium falciparum malaria and treatment effects on gametocytic development]. Biomedica : revista del Instituto Nacional de Salud. 2009 Jun;29(2):307-19. PubMed PMID: 20128355. Epub 2010/02/05. Estudio piloto de la eficacia y de los efectos sobre los gametocitos del esquema artesunato-mefloquina-primaquina para la malaria por Plasmodium falciparum. spa.
20. El-Sayed B, El-Zaki SE, Babiker H, Gadalla N, Ageep T, Mansour F, et al. A randomized open-label trial of artesunate- sulfadoxine-pyrimethamine with or without primaquine for elimination of sub-microscopic P. falciparum parasitaemia and gametocyte carriage in eastern Sudan. PloS one. 2007;2(12):e1311. PubMed PMID: 18074034. Pubmed Central PMCID: 2111052. Epub 2007/12/13. eng.
21. Shekalaghe SA, ter Braak R, Daou M, Kavishe R, van den Bijllaardt W, van den Bosch S, et al. In Tanzania, hemolysis after a single dose of primaquine coadministered with an artemisinin is not restricted to glucose-6-phosphate dehydrogenase-deficient (G6PD A-) individuals. Antimicrobial agents and chemotherapy. 2010 May;54(5):1762-8. PubMed PMID: 20194698. Pubmed Central PMCID: 2863610. Epub 2010/03/03. eng.

22. Giao PT, de Vries PJ, Hung le Q, Binh TQ, Nam NV, Kager PA. CV8, a new combination of dihydroartemisinin, piperaquine, trimethoprim and primaquine, compared with atovaquone-proguanil against falciparum malaria in Vietnam. Tropical medicine & international health : TM & IH. 2004 Feb;9(2):209-16. PubMed PMID: 15040557. Epub 2004/03/26. eng.
23. Kamtekar KD, Gogtay NJ, Dalvi SS, Karnad DR, Chogle AR, Aigal U, et al. A prospective study evaluating the efficacy of a single, 45-mg dose of primaquine, as a gametocytocidal agent, in patients with Plasmodium falciparum malaria in Mumbai, India. Annals of tropical medicine and parasitology. 2004 Jul;98(5):453-8. PubMed PMID: 15257794. Epub 2004/07/20. eng.
24. Suputtamongkol Y, Chindarat S, Silpasakorn S, Chaikachonpatd S, Lim K, Chanthapakajee K, et al. The efficacy of combined mefloquine-artesunate versus mefloquine-primaquine on subsequent development of Plasmodium falciparum gametocytemia. The American journal of tropical medicine and hygiene. 2003 May;68(5):620-3. PubMed PMID: 12812357. Epub 2003/06/19. eng.
25. White NJ. Primaquine to prevent transmission of falciparum malaria. The Lancet infectious diseases. 2012 Nov 22. PubMed PMID: 23182932. Epub 2012/11/28. Eng.
26. Eziefula AC, Staedke SG, Yeung S, Webb E, Kamya M, White NJ, et al. Study protocol for a randomised controlled double-blinded trial of the dose-dependent efficacy and safety of primaquine for clearance of gametocytes in children with uncomplicated falciparum malaria in Uganda. BMJ open. 2013;3(3). PubMed PMID: 23535703. Pubmed Central PMCID: 3612795. Epub 2013/03/29. eng.
27. Eziefula AC, Bousema T, Yeung S, Kamya M, Owaraganise A, Gabagaya G, et al. Single dose primaquine for clearance of Plasmodium falciparum gametocytes in children with uncomplicated malaria in Uganda: a randomised, controlled, double-blind, dose-ranging trial. The Lancet infectious diseases. 2014 Feb;14(2):130-9. PubMed PMID: 24239324. Epub 2013/11/19. eng.
28. Beutler E, Mitchell M. Special modifications of the fluorescent screening method for glucose-6-phosphate dehydrogenase deficiency. Blood. 1968 Nov;32(5):816-8. PubMed PMID: 4386875. Epub 1968/11/01. eng.
29. Boom R, Sol C, Wertheim-van Dillen P. Rapid purification of ribosomal RNAs from neutral agarose gels. Nucleic acids research. 1990 Apr 25;18(8):2195. PubMed PMID: 2336409. Pubmed Central PMCID: 330721.
30. Schneider P, Schoone G, Schallig H, Verhage D, Telgt D, Eling W, et al. Quantification of Plasmodium falciparum gametocytes in differential stages of development by quantitative nucleic acid sequence-based amplification. Molecular and biochemical parasitology. 2004 Sep;137(1):35-41. PubMed PMID: 15279949. Epub 2004/07/29. eng.
31. Shekalaghe S, Drakeley C, Gosling R, Ndaro A, van Meegeren M, Enevold A, et al. Primaquine Clears Submicroscopic Plasmodium falciparum Gametocytes that Persist after Treatment with Sulphadoxine-Pyrimethamine and Artesunate. PLoSONE. 2007;2(10):e1023. PubMed PMID: 802.
32. Okell LC, Griffin J, Kleinschmidt I, Hollingsworth TD, Churcher T, White M, et al. The potential contribution of mass treatment to the control of Plasmodium falciparum malaria. PloS one. 2011;6(5):e20179.

Supplements, appendices and other documents

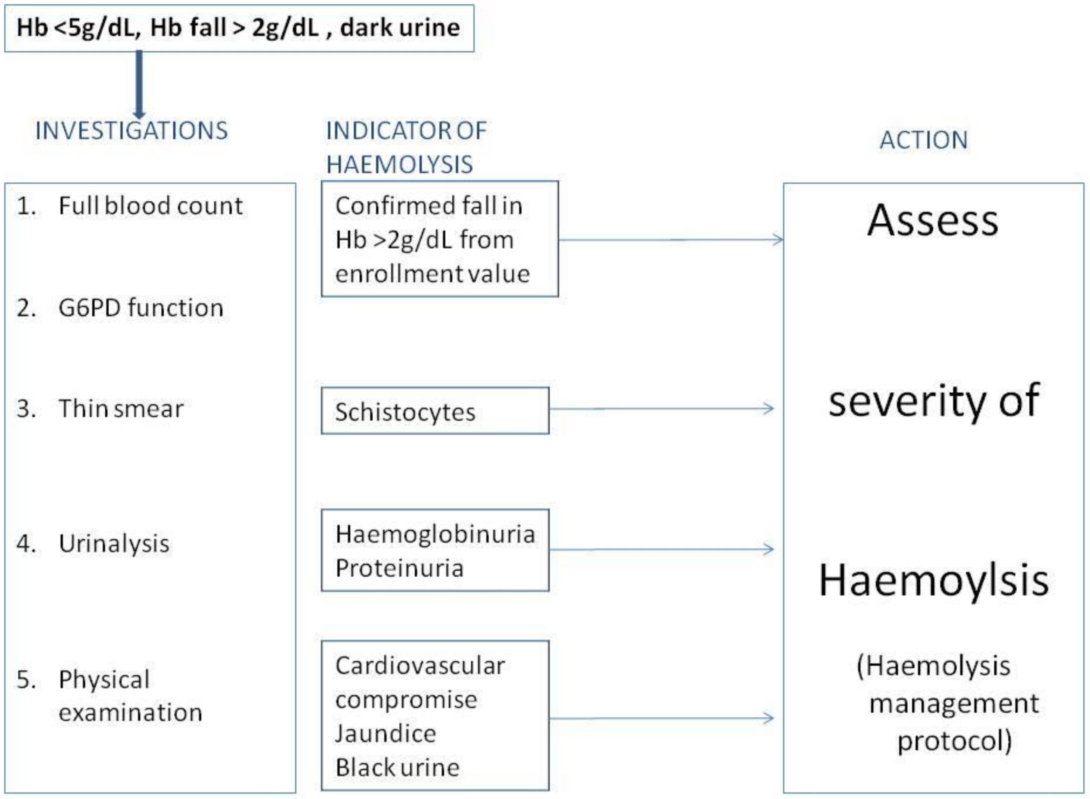

Figure 1: Flowchart for investigating haemolysis

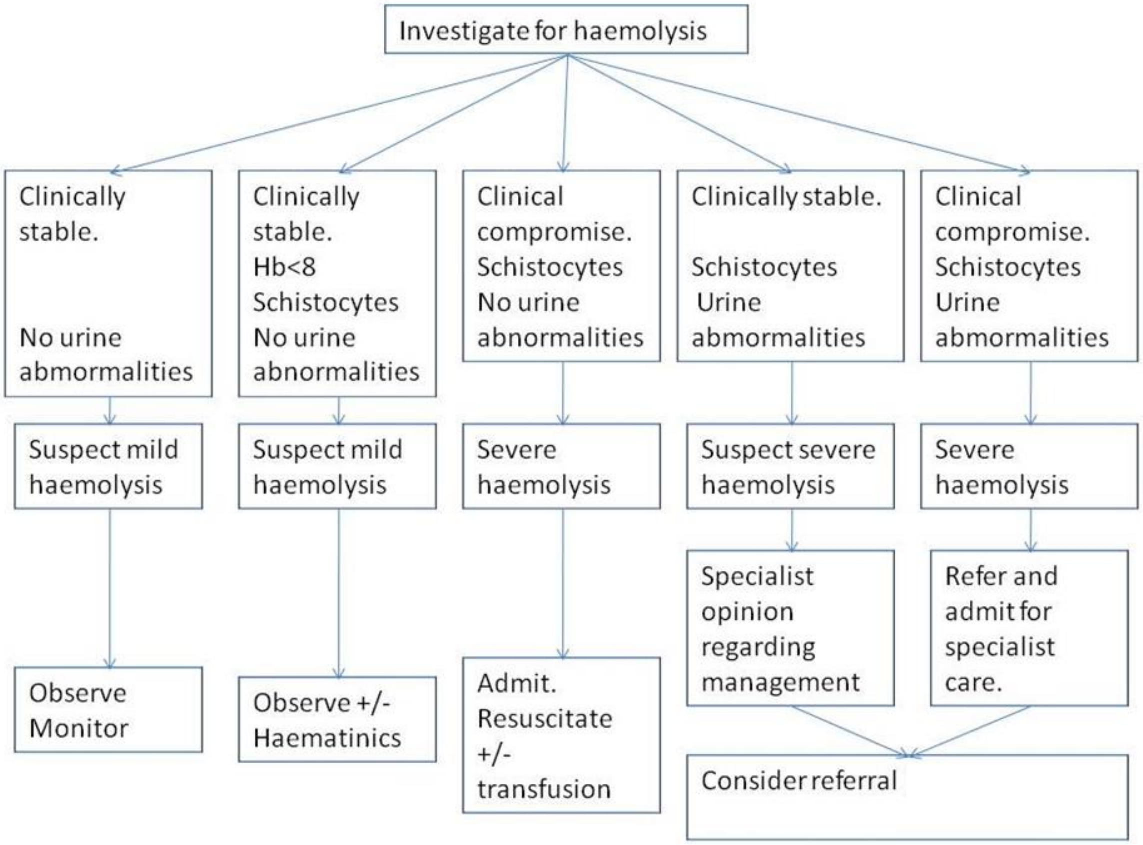

Figure 2: Management of haemolysis

15 SCHEMATIC OF STUDY DESIGN:

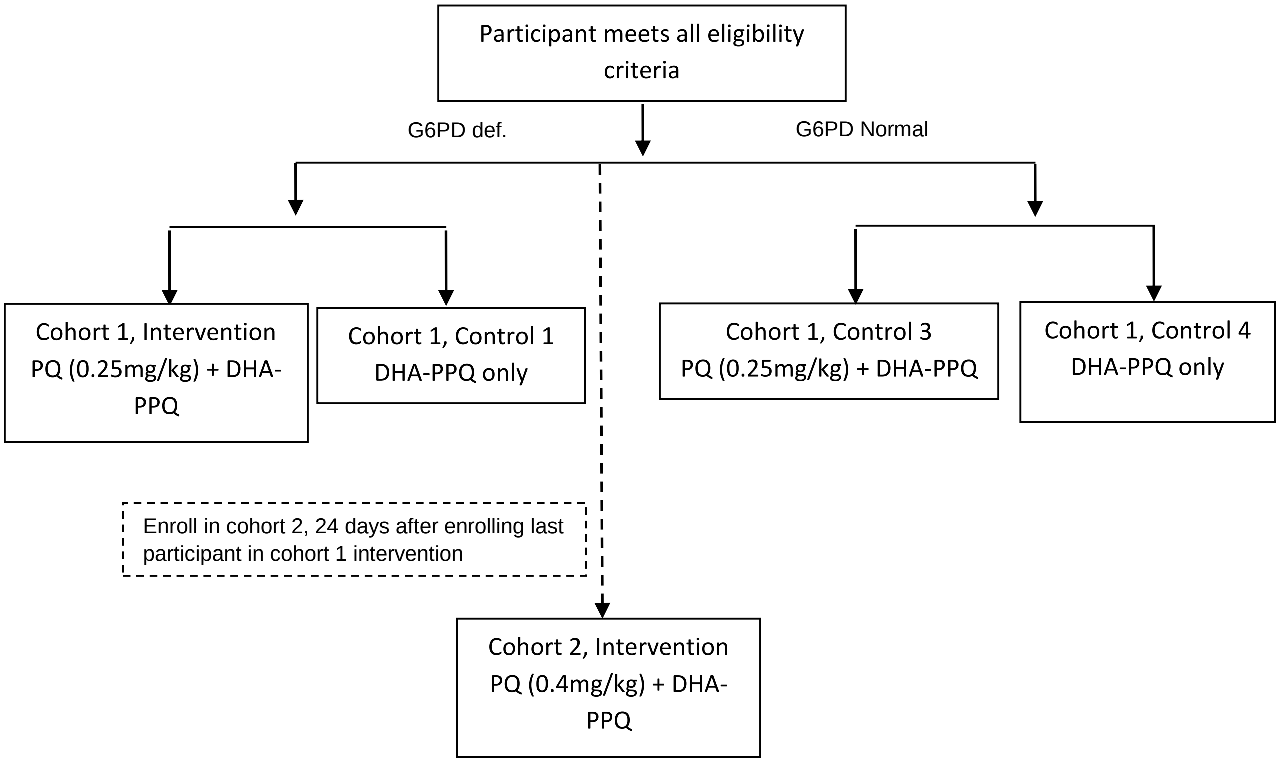

Supplement: S2 File — (PDF) [file pone.0190272.s007.pdf]
